# Shoot and root single cell sequencing reveals tissue- and daytime-specific transcriptome profiles

**DOI:** 10.1093/plphys/kiab537

**Published:** 2021-11-19

**Authors:** Federico Apelt, Eleni Mavrothalassiti, Saurabh Gupta, Frank Machin, Justyna Jadwiga Olas, Maria Grazia Annunziata, Dana Schindelasch, Friedrich Kragler

**Affiliations:** 1 Max Planck Institute of Molecular Plant Physiology, Wissenschaftspark Golm, Am Mühlenberg 1, 14476 Potsdam, Germany; 2 University of Potsdam, Institute of Biochemistry and Biology, Department of Molecular Biology, Karl-Liebknecht-Strasse 24-25, Haus 20, 14476 Potsdam, Germany

## Abstract

Although several large-scale single-cell RNA sequencing (scRNAseq) studies addressing the root of Arabidopsis (*Arabidopsis thaliana*) have been published, there is still need for a de novo reference map for both root and especially above-ground cell types. As the plants’ transcriptome substantially changes throughout the day, shaped by the circadian clock, we performed scRNAseq on both Arabidopsis root and above-ground tissues at defined times of the day. For the root scRNAseq analysis, we used tissue-specific reporter lines grown on plates and harvested at the end of the day (ED). In addition, we submitted above-ground tissues from plants grown on soil at ED and end of the night to scRNAseq, which allowed us to identify common cell types/markers between root and shoot and uncover transcriptome changes to above-ground tissues depending on the time of the day. The dataset was also exploited beyond the traditional scRNAseq analysis to investigate non-annotated and di-cistronic transcripts. We experimentally confirmed the predicted presence of some of these transcripts and also addressed the potential function of a previously unidentified marker gene for dividing cells. In summary, this work provides insights into the spatial control of gene expression from nearly 70,000 cells of Arabidopsis for below- and whole above-ground tissue at single-cell resolution at defined time points.

## Teaser

Analysis of Arabidopsis single cell transcriptome of roots (at end of day) and above ground tissues (at the end of day and the end of night) revealed marker genes, cell type differences, and similarities.

## Introduction

Bulk collection of tissue-specific cell populations by fluorescence-activated cell sorting (FACS) has provided valuable insights of gene expression within, for example Arabidopsis (*Arabidopsis thaliana*) root tissues, but this approach masks information about cellular heterogeneity within a given tissue (Birnbaum et al., 2003; Brady et al., 2007). Nowadays, high-throughput single-cell RNA sequencing (scRNAseq) is widely used technologies to study transcriptomes at single-cell resolution by dissecting cellular heterogeneity. scRNAseq techniques are more challenging to apply to plants, mainly due to the size-variable plant cell populations ranging from 10 micrometer to several hundred micrometers and the necessary digestion of cell walls (protoplasting) that substantially changes their transcriptome as seen with single-cell sequencing after FACS (Birnbaum et al., 2003).

However, recent studies have successfully implemented scRNAseq methods for plant tissues. In particular, dissecting gene expression of root and shoot cell types provides insights into tissue-specific gene activity and cellular phase changes, that is dividing versus maturing cells, into cellular metabolism, and into developmental and regulatory pathways. In detail, the root of Arabidopsis with its relatively few and distinct cell types has served as an ideal tissue for scRNAseq methods such as Drop-seq and 10× Genomics (Denyer et al., 2019; Jean-Baptiste et al., 2019; Ryu et al., 2019; Shulse et al., 2019; Turco et al., 2019; Zhang et al., 2019; Wendrich et al., 2020). Recently, the method was expanded to above-ground tissues, producing single-cell transcriptomic maps of the leaf vasculature and the shoot apices of Arabidopsis (Kim et al., 2021; Zhang et al., 2021).

Nonetheless, there is still need for a de novo reference map for both root and especially for the less well-defined cell types of the aerial (above-ground) parts of Arabidopsis at clearly defined diel time points of harvest and growth conditions. Most available scRNAseq studies do not indicate an exact time point of harvest although it is well established that the transcriptome changes significantly depending on the circadian clock especially between the end of the day (ED) and the end of the night (EN). We address these aspects and provide insights into the spatial control of gene expression at single-cell resolution from nearly 70,000 cells of Arabidopsis for below- and, for the first time, whole above-ground tissues at ED and EN time points. This approach allows us to answer questions, such as, what common cell types and corresponding markers can be found between root and shoot, or how does the single-cell transcriptome change in certain tissues depending on the time of the diel cycle?

## Results

To profile both Arabidopsis root and above-ground cells in single-cell resolution and to generate the necessary scRNAseq library, we customized the Drop-seq protocol (Macosko et al., 2015) for plant cells (Supplemental Methods S1, S2 and Supplemental Figure S1).

**Figure 1:**
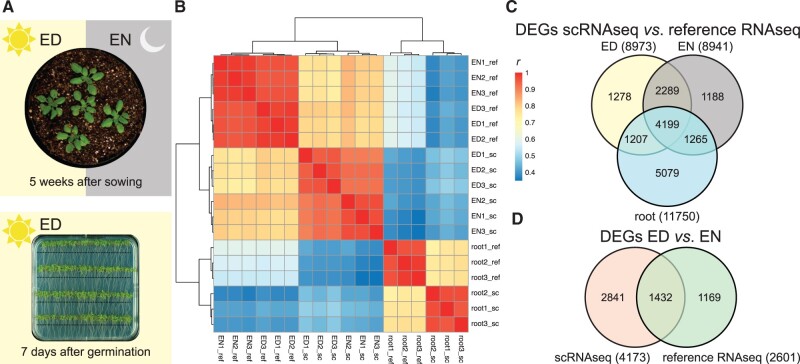
Experimental overview with clustering and DEGs. A, Roots were harvested at the ED from 7-d-old plants approx. 1 cm below the hypocotyl (indicated by the black line); the above-ground tissue was harvested at the ED and EN from 5-week-old plants. For the single-cell samples, the tissue was harvested 75 min before the ED (or EN) time point and used for protoplast preparation. For the reference RNAseq libraries, the tissue was harvested 15 min before the ED (or EN) time point and shock frozen in liquid nitrogen. B, Hierarchical clustering of the sequenced scRNAseq (sc) and reference RNAseq (ref) libraries using Pearson’s correlation coefficient (*r*) as distance measure. C, Venn diagram of DEGs between scRNAseq and reference RNAseq for the different timepoints and tissues. D, Venn diagram of DEGs between ED and EN for scRNAseq and reference RNAseq. DEGs are defined as |log_2_FC| ≥ 1 and FDR of 0.05.

For root single-cell analysis, we used three tissue-specific reporter lines grown 7 d under neutral day (ND) conditions (12/12-h photoperiod) on 0.5× MS plates supplied with 0.6% w/v sucrose. The used reporter lines were expressing the *H2B-Venus* fluorescent marker under tissue-specific promoters marking the phloem pole pericycle (PPP; line V161), the xylem pole pericycle (XPP; line V171), and post-meristem differentiated cells (line V311) (Machin et al., 2019). For each reporter line, the roots were harvested 1 cm below the hypocotyl (Figure  1, A), 75 min before the ED for protoplasting followed by scRNAseq (Drop-seq) co-encapsulation at the ED time point. We also collected all tissues of 5-weeks-old non-flowering *A.* *thaliana* Col-0 plants grown under ND conditions on soil at ED and at EN (Figure  1, A) above the hypocotyl, which includes 8–10 true rosette leaves, petioles, and shoot apical meristem (SAM). For both, roots and above-ground tissues, we collected three replicates for each time point and corresponding reference RNA libraries of the same plant material at the same time point to investigate the effect of protoplasting and scRNAseq protocol (Supplemental Methods S3). The cDNA libraries for the reference samples were produced with the same protocol as the single-cell libraries, that is a barcode-like primer (Supplemental Table S1) was used that resembles the barcoded-beads.

Prior to deep sequencing, the quality of the cDNA libraries was confirmed by RT-PCR (Supplemental Methods S4 and Supplemental Figure S2). Presence of *ACTIN2* (*ACT2*, AT3G18780) verified successful RNA capture and cDNA production, and presence of *SUCROSE-PROTON SYMPORTER 2* (*SUC2*, AT1G22710), a well-known phloem companion cell marker gene, confirmed protoplasting of vascular phloem cells in all three single-cell libraries. Therefore, we concluded that our scRNAseq libraries contain not only transcripts from the most abundant or easily protoplasted cells, but also transcripts from cells present in deeper layers and with a more rigid cell wall. Rare cell types like the root QC indicated by *WUSCHEL RELATED HOMEOBOX 5* (*WOX5*, AT3G11260) (Sarkar et al., 2007) were not detected in the single-cell libraries. This was expected because not all cell types are efficiently protoplasted and because only a small fraction of the protoplasts is successfully barcoded further reducing the probability of rare cells to be represented in the cDNA libraries. Finally, the detection of *H2B-Venus* transcripts in the root libraries validated the presence of the cell types of interest: PPP, XPP, and post meristem/differentiating cells.

Also, the above-ground tissue cDNA libraries were confirmed using *ACT2*, *KNOTTED-LIKE FROM ARABIDOPSIS THALIANA* (*KNAT1*, AT4G08150), and *SUC2* transcript presence as markers for successful cDNA production and the presence of companion cells, vascular cambium/meristem, and stomata cells. In addition, to confirm that the light treatment and time of harvest reflected ED and EN, two transcripts: *LUX ARRHYTHMO* (*LUX*, AT3G46640), peaking at the ED, and *LATE ELONGATED HYPOCOTYL* (*LHY*, AT1G01060), peaking around EN were used to validate that the libraries reflect the expected diurnal transcriptomic profiles (Supplemental Figure S2, B).

The quality controlled scRNAseq and reference RNAseq libraries were submitted to deep sequencing (Supplemental Tables S2, S3) and established algorithms for unsupervised clustering (Stuart et al., 2019) were applied to characterize the identities of the cell types represented in each cluster via highly specific known and previously unidentified marker genes. In general, we could identify the main root, shoot, and leaf tissues. We also searched for common markers in root and shoot tissues, which were analyzed in more detail and experimentally confirmed using RNA in situ hybridization (see below). Furthermore, we investigated the tissue-specific differences of ED and EN marker transcripts, di-cistronic transcripts, and of non-annotated transcripts.

### Differential gene expression of single-cell and reference tissue transcriptomes

In order to compare the scRNAseq and reference RNAseq libraries, we performed principal component analysis (PCA), correlation-based clustering, and differential gene expression analysis. The root and shoot libraries are separated among PC1 (32.93% of variance) and scRNAseq and reference RNAseq libraries are separated among PC2 (29.34% of variance) (Supplemental Figure S3, A). Similarly, root and shoot libraries build the two main clusters that are subdivided into scRNAseq and reference RNAseq (Figure  1, B and Supplemental Figure S3, B). As expected, the clustering indicates that the tissue type (root versus shoot) along with the growth condition (plates and soil, respectively) has the biggest influence on the transcriptome, followed by the method (scRNAseq versus reference RNAseq) and the time point of harvest (ED versus EN). The correlation among replicates is high (*r* > 0.9), thus, we obtained a good reproducibility of the samples (Supplemental Figure S3, B). Also, the correlation between gene expression of scRNAseq libraries and their corresponding reference RNAseq libraries is generally high (*r* = 0.77–0.82; Supplemental Figure S3, C). However, for the genes previously described to be induced (Birnbaum et al., 2003; 346 genes) or differentially expressed upon protoplasting roots (Denyer et al., 2019; 6,063 genes) and shoots (Kim et al., 2021; 8,845 genes), the correlation is lower in the respective tissues (*r* = 0.48, 0.46, and 0.67, respectively; Supplemental Figure S3, D).

Next, we tested for differential expression (DE) of genes between scRNAseq libraries and their corresponding reference RNAseq libraries (|log_2_FC| > 1; Figure  1, C and Supplemental Table S4). For the root, we found 11,750 differentially expressed genes (DEGs) (6,121 up- and 5,629 down-regulated), which largely overlap with the published 6,063 genes previously shown to be DE upon root protoplasting (Denyer et al., 2019; overlap: 3,646; Supplemental Figure S4, A). Within the shoot libraries, we found nearly 9,000 DEGs for both time points (ED 8,973 DEGs with 5,201 up- and 3,772 down-regulated genes; EN 8,941 DEGs with 4,884 up- and 4,057 down-regulated genes) with an overlap of 6,488 genes. These genes also largely overlap with the published 8,845 genes shown to be DE upon shoot protoplasting (Kim et al., 2021; overlap: 2,920; Supplemental Figure S4, A). Furthermore, the DEGs found in root (11,750) and shoot at ED and EN (6,488) also largely overlap (4,199). For analysis of MapMan annotation of these protoplast-induced (up-regulated) genes, see Supplemental Text S1. Thus, a substantial proportion of genes is affected by the experimental procedure necessary for single-cell sequencing that is largely reproducible across different tissues, time points, and studies.

Lastly, we also checked for DEGs between ED and EN time points in the shoot for scRNAseq (4,273 genes) and reference RNAseq libraries (2,601 genes) with an overlap of 1,432 genes (Figure  1, D). As expected, this list contains typical diurnal clock-related genes such as *LHY*, *CCA1*, and *GLYCINE RICH PROTEIN 7* (*GRP7*, AT2G21660) among the most significantly changed genes. This is also reflected by GO enrichment analysis, where “circadian rhythm” (fold enrichment: 8.91; *P*-value: 4.05*E*−18) and “response to light intensity” (fold enrichment: 5.23; *P*-value: 1.34*E*−07) are among the most enriched categories (Supplemental Table S5).

### Clustering scRNAseq libraries

To facilitate the identification of cell types, we used Seurat to separate the scRNAseq data into clusters for root and ED and EN shoot datasets. Prior to clustering, we filtered out the data of cells with too few reads, with large amounts of plastidial transcripts, or with unspecific gene-expression that are likely to be noise (Supplemental Figure S5 and Supplemental Methods S5). For root, we obtained 19,153 cells from three replicates. For shoot, we obtained 18,313 and 31,665 cells from the three ED and EN replicates, respectively (Supplemental Table S2). The average number of transcripts/genes per cell in root, ED, and EN was 1,994/822, 816/422, and 793/377, respectively (Supplemental Tables S6–S9).

Note that while the information content per cell seems lower than in other published scRNAseq datasets, one must consider two things: Firstly, most studies use a different method, that is 10× Chromium which provides better read coverage per cell. Secondly, these numbers are highly cutoff-dependent. For example, by increasing the minimum number of transcripts/genes per cell in root from 500/200 to 2,000/1,000, the average number of transcripts/genes per cell rises from 1,994/822 to 4,386/2,040. However, instead of nearly 20,000 cells, only <3,000 cells with high depth would remain for clustering. As the main purpose of this study was the discovery of potentially rare cell-types and transcripts, we decided for a lower cutoff, which results in a larger number of analyzed cells and subsequently more clusters to be analyzed further.

For root, the analysis resulted in 35 clusters, whereas for the individual ED and EN datasets, we obtained 37 and 25 clusters, respectively. We also combined the scRNAseq data from ED and EN to obtain a pooled ED/EN dataset, for which the number of clusters was 16. Finally, we confirmed that cells from the same replicates are well distributed across all clusters (Supplemental Figure S6).

In order to evaluate the robustness of each clustering, we randomly subsetted 80% of our library reads (100 times) and repeated the computational pipeline. We then checked what proportion of cells (0–100%) that previously formed a cluster are still co-occurring in a single cluster when using the subsetted reads for clustering (Supplemental Figure S7). The average co-occurrence was used to score the robustness for each cluster. This analysis revealed that majority of clusters is highly robust with >50% co-occurrence.

Next, the most significantly enriched genes for each cluster were identified (Supplemental Tables S10–S13), in order to annotate cell types/developmental stages to clusters by comparison with tissue-type-specific transcriptome data from the ATH1 database (Schmid et al., 2005) and by the presence of known marker genes.

### Cluster identification of above-ground tissue scRNAseq data

For this study, whole above-ground tissue harvested at two different time points (ED and EN) was submitted to scRNAseq. Thus, the transcriptome data represent a mixture of leaves of different developmental and physiological stages and shoot apices. The cluster annotation was first performed for the individual ED and EN and then for the combination of the ED and EN datasets.

Unlike the Arabidopsis root tissues, different cell types of leaves at different developmental stages are less well understood. There is also a lack of high-resolution tissue-specific transcript data and of available transgenic marker lines. Because of this, the shoot-derived clusters were annotated in a broader fashion than the root-derived clusters.

**Figure 2:**
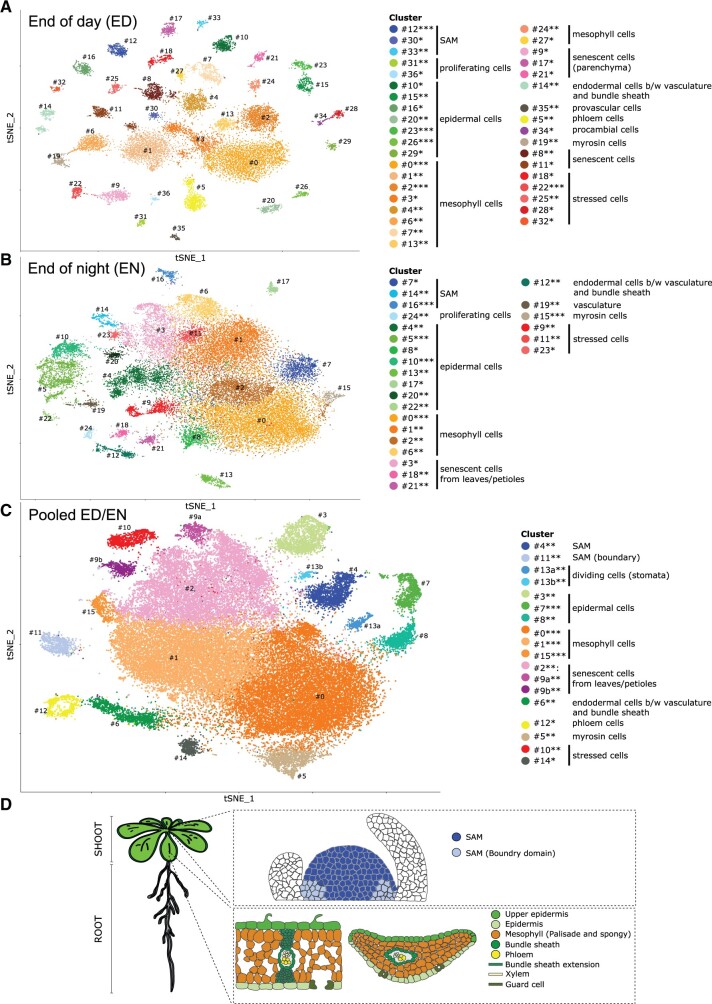
Single-cell transcriptome of *A. thaliana* above-ground tissue. A, *t*-SNE projection plot of 37 clusters identified from 18,313 cells in Col-0 rosette tissues harvested at the ED (*n* = 3 replicates). B, *t*-SNE visualization of 25 clusters identified from 31,665 cells in Col-0 rosette tissues harvested at the EN (*n* = 3 replicates). C, *t*-SNE projection plot showing 16 main clusters identified from 49,978 pooled cells in Col-0 rosette tissues harvested ED and EN (*n* = 6 replicates). Each dot represents the transcriptome from one cell. Cells represented by the same colors correspond to the same cluster. Cells identified in different clusters but belonging to the same tissue types are represented with similar colors. The cluster robustness was scored based on cell co-occurrence in subsampled data 75%–100% (***), 50%–75% (**), 25%–50% (*) (see “Materials and methods”; Supplemental Figure S7). D, Schematic representation of rosette cell types of Arabidopsis plants. Upper panel represents longitudinal section through the meristem, whereas lower panel shows longitudinal and cross section of the leaf.

First, we separately clustered the cells from ED and EN samples (Figure  2, A and B; for detailed description of the individual cluster, see Supplemental Texts S2, S3). In summary, we detected all main tissues within the clustering, however, the strong presence of photosynthesis-related transcripts dominates the dataset. Therefore, slight differences in types of photosynthetically active cells seem to be lost as the cells cluster together due to the strong expression of these genes.

In order to ascertain tissue-type/cluster identification, we pooled the datasets from both time points (Figure  2, C) by normalizing the consistent differences between ED and EN (see “Materials and methods”). Thus, each cluster consists of a well-mixed number of cells from ED and EN (Supplemental Figure S6, D). The information how the cells map between the individual clustering and the pooled ED/EN clustering (Supplemental Table S14) also supported the annotation in the pooled ED/EN dataset, which forms 16 clusters based on 49,978 cells with specific marker genes (Supplemental Table S13) that facilitate further dissection of rosette anatomy at the cell type level.

We identified clusters representing the main tissues of the shoot (Figure  2), that is epidermal cells, mesophyll cells, SAM, phloem cells, and myrosin cells. In addition, we found clusters annotated as senescent leaf cells, dividing cells, and stressed cells (for detailed information, see Supplemental Text S4 and Supplemental Figure S8).

Notably, we do not find stomata as a unique cluster in our dataset, instead key transcripts known to specifically regulate stomatal development are found distributed across several clusters. We therefore concluded that the dominant photosynthesis expression pattern prevents these cells from clustering as a specific cell type.

The MapMan functional category “Carbohydrate metabolism” provided a good example for ED and EN differential gene enrichment, although this category did not fall into the highly enriched ones. In the subcategory “starch metabolism biosynthesis” created for the pooled shoot ED+EN, a clear enrichment was found for the cluster 15 (and 1), representing mesophyll cells where most of the starch is synthesized (Supplemental Figure S9). To further investigate the potential functional differences between ED and EN, we display the datasets of both time points separately (Supplemental Figure S9). Notably, starch metabolism biosynthesis subcategory was enriched for the EN, while starch metabolism degradation was enriched at ED.

### Tissue-specific differences in ED versus EN

Notably we see a change of cluster distribution in the clustering between ED and EN. For example, at ED 10 mesophyll clusters and at EN only 4 mesophyll clusters appear to be present (Figure  2, A and B). To gain insights into their differences, we performed MapMan category enrichment analysis across the clusters of ED, EN, and pooled ED/EN datasets (Supplemental Figure S10). At ED, 6 out of 10 mesophyll clusters are enriched for genes related to photosynthesis (including cell-rich 0, 2, 4, and the clusters 7, 13, 24). At EN, two out of four mesophyll clusters show reduced photosynthesis gene expression (including cell-rich clusters 0 and 2). This is also reflected in the pooled ED/EN clustering, where cluster 1, which is enriched for photosynthesis-related genes, contains ED clusters 0 and 2 cells from EN cluster 1 that are also enriched in photosynthesis gene expression (Supplemental Table S14).

Furthermore, the mesophyll clusters show distinct metabolic features in such that sucrose–phosphate synthase encoding transcripts of *SUCROSE PHOSPHATE SYNTHASE 4F* (*SPS4F/SPSC*, AT4G10120) and *SUCROSE PHOSPHATE SYNTHASE 2F* (*SPS2F/SPSA2*, AT5G11110) (Volkert et al., 2014) was low at ED in all mesophyll clusters and even absent in mesophyll cluster 27. In contrast, these transcripts are relatively highly expressed in all EN mesophyll clusters. This indicates that the ED mesophyll cluster 27 contains starving cells with low photosynthetic net production which is in line with the absence of transcripts for the chloroplastic antiporter *CA*(*2+*)/*H*(*+*) *ANTIPORTER1* (*CCHA1*, AT1G64150) necessary for the assembly of the PSII core subunits and the oxygen-evolving complex (Wang et al., 2016). Another example of distinct sugar metabolism was found in the ED mesophyll cluster 7 with the highest expression levels of all four starch synthases (*SS1*, AT5G24300, *SS2*, AT1G74020, *SS3*, AT1G74020, and *SS4*, AT4G18240) genes relative to the other ED mesophyll clusters. Sucrose production seemed to be high in clusters 4 and 7 marked by relative high expression of *SUCROSE SYNTHASE 1* (*SUS1*, AT5G20830) and *SUS2* (AT5G49190) with the latter not detected in in clusters 2, 3, 6, 13, 24, and 27.

**Figure 3:**
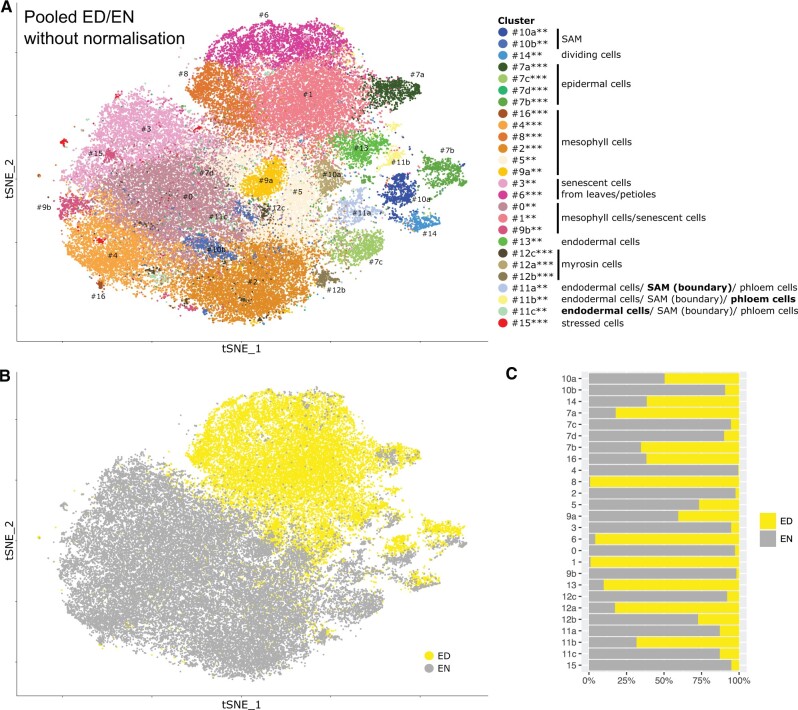
Singe-cell transcriptome of *A. thaliana* above-ground tissue without batch normalization. A, *t*-SNE projection plot showing 17 main clusters identified from 49,978 pooled cells in Col-0 rosette tissues harvested ED and EN (*n *=* *6 replicates). The cells are the same as shown in panel C, however, this clustering was performed without batch normalization, that is without using Harmony (see “Materials and methods”). Each dot represents the transcriptome from one cell. Cells represented by the same colors correspond to the same cluster. Cells identified in different clusters but belonging to the same tissue types are represented with similar colors. The cluster robustness was scored based on cell co-occurrence in subsampled data 75%–100% (***), 50%–75% (**), 25%–50% (*) (see “Materials and methods”; Supplemental Figure S7). B, Same *t*-SNE projection plot as in A, but cells of the same color correspond to the same time point of harvest (ED or EN). C, Fraction of cells belonging to ED or EN for each cluster.

To further characterize circadian rhythm (ED or EN)-derived changes at the tissue level, we analyzed the datasets regarding complexity. Complexity is here defined as the variation in response, such that a tissue exhibiting a uniform response to a stimulus would be considered less complex than a tissue responding heterogeneously. For this analysis, the clustering of the shoot scRNAseq dataset was repeated without the previously described normalization used to create the pooled ED/EN dataset (Figure  3, A and B and Supplemental Table S15). For each cell, we mapped the new cluster assignment to the existing annotations of the pooled ED/EN clustering, which was sufficient to annotate nearly all clusters (Supplemental Table S16). Thus, we could investigate which cells cluster based on their tissue identity or time of harvest. Clusters of dividing cells (#14), epidermis (#7), SAM (#10), and partly myrosin cells (#12) have a mixture of ED and EN cells (Figure  3, C) and, thus, display no strong difference depending on the time of the day. A clear ED versus EN separation was observed for clusters of mesophyll cells, senescent cells, and partly myrosin cells. Some clusters merged mesophyll and senescent cells (#0, #1, and #9b); however, the clusters strictly separate into cells from ED or EN.

Importantly, this approach allowed us to investigate tissue-specific markers depending on the time of the day. In order to find ED- or EN-specific markers, we analyzed ED and EN cells separately for (i) clusters with a mixture of ED and EN cells and (ii) clusters with same annotation but split for ED and EN cells (Supplemental Table S17). For example, in the mixed clusters of dividing cells, SAM, and epidermis, we found several cell-cycle-related genes (G1/G2 phase) to be markers in either ED or EN cells (but not both). Interestingly, these genes were not differentially expressed between ED and EN cells on the global scale. Thus, we identified a circadian response of genes related to cell growth such as *GRP7* that would have been undetected without looking separately at the clusters and time points.

### Cluster annotation of root scRNAseq data

In this study, protoplasts were made from approximately 2–3-cm long roots and, thus, included the root apical meristem (RAM) and mature regions of the root with different stages of lateral root initiation sites (Supplemental Figure S1, A). The root dataset was generated at ED using marker lines for tissues of interest: PPP, XPP, and post-meristem differentiated cells (Machin et al., 2019). Since there are several published scRNAseq datasets of roots, we provide only a summary of the clusters and their annotation (for detailed information, see Supplemental Text S5).

**Figure 4:**
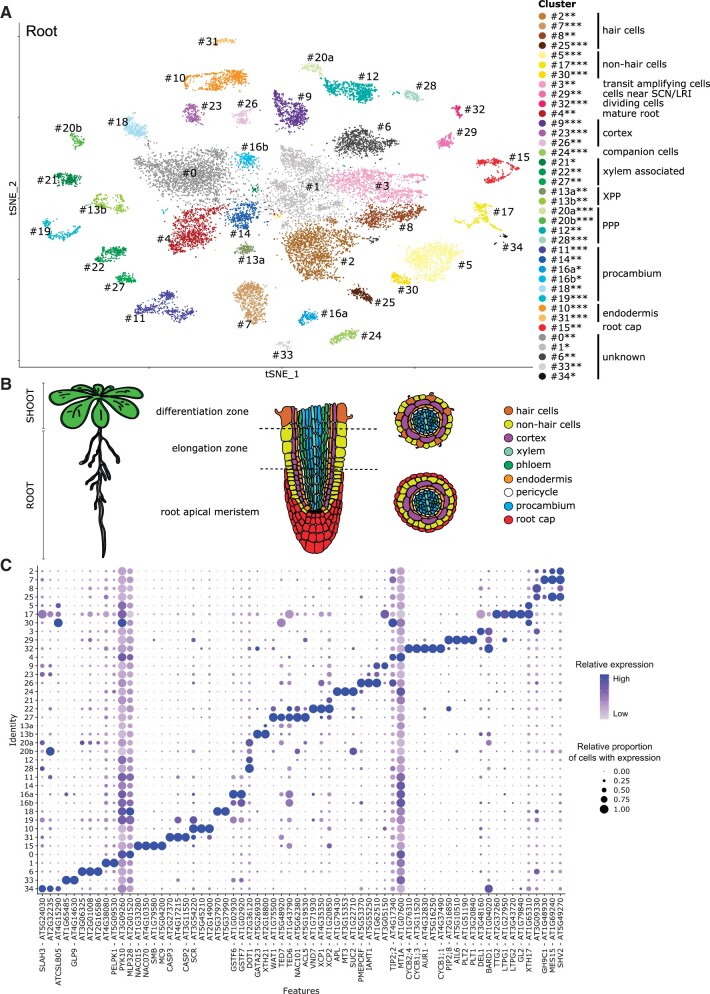
Singe-cell transcriptome of *A. thaliana* roots. A, *t*-SNE projection plot of 35 main clusters identified from 19,153 cells in Col-0 root tissues (*n *=* *3 replicates). Each dot represents the transcriptome from one cell. Cells represented by the same colors correspond to the same cluster. Cells identified in different clusters but belonging to the same tissue types are represented with similar colors. The cluster robustness was scored based on cell co-occurrence in subsampled data 75%–100% (***), 50%–75% (**), 25%–50% (*) (see “Materials and methods”; Supplemental Figure S7). B, Schematic representation of the root cell types in Arabidopsis plants. Middle panel represents longitudinal section through the root, whereas the right panel shows cross section through the differentiation (upper) and meristematic (bottom) zone of the root. C, Dot plot representing transcript accumulation of known and non-annotated marker genes for each cluster. The color scale denotes the relative expression (average per cell). The dot size denotes the relative proportion of cells with expression. Color scale and dot size are scaled to the minimum/maximum expression level and proportion of cells with expression, respectively, of the transcript within the clusters; for the absolute percentages, see Supplemental Table S20. SCN, stem cell niche; LRI, lateral root initials; XPP, xylem pole pericyle; PPP, phloem pole pericyle.

In short, we identified clusters representing the main tissues of the primary root (Figure  4) including tissue subdivisions as in the phloem and xylem poles of the pericycle. In addition, we identified different developmental stages of the vasculature and distinct cell types such as the epidermis, endodermis, and root cap. Further, we found clusters that are specified by dominant expression patterns possessed by cells at particular developmental stages. For example, we were able to identify a cluster for the cells nearest to the stem cell niche and lateral root initials indicated by the high levels of *PLETHORA 2* (*PLT2*, AT1G51190) transcripts (cluster 29) (Shimotohno et al., 2018) and *GATA TRANSCRIPTION FACTOR 23* (*GATA23*, AT5G26930) (XPP cluster 13b) (De Rybel et al., 2010), respectively. Cells neighboring emerging lateral roots were marked by *ORESARA 1* (*ORE1*, AT5G39610) transcript (procambium annotated clusters 11 and 19) which is a programmed cell death/autophagy marker specifically expressed in the cells surrounding the emerging lateral root (Escamez et al., 2020). Also, we detected cells undergoing post-meristem differentiation indicated by the used V311 marker line (Cluster 16b) (Supplemental Figure S11, D).

One interesting aspect was that among the different root cell types, the root cap (cluster 15) shows significant enrichment of several MapMan functional categories related to secondary metabolism (Supplemental Figure S12) pinpointing a particular involvement of the root cap in the interaction with and protection against the surrounding environment (see Supplemental Text S6 for details).

### Common traits in root and shoot

A closer look at those highly enriched MapMan categories unveiled specific tissue and/or time of the day gene responses which could be also considered a validation of the experimental procedure used. For example, the category “chromatin organization” was highly enriched in the root cluster 3, as well as in the shoot ED/EN cluster 4, comprising amplifying cells of RAM and SAM, respectively (Supplemental Figure S13). Both chromatin organization categories of the histones H2A and H3 were highly enriched in those cell types. It is well known that H2A and H3 histones form the nucleosomes (Zhang et al., 2015) during DNA replication in order to maintain the proper chromatin organization. We can hypothesize that in RAM and SAM, where stem cells reside and greater DNA synthesis occurs, there is a high demand for histone synthesis and deposition to avoid developmental defects (Hashimura and Ueguchi, 2011).

We also investigated potential overlaps in root and shoot clusters by calculating the Jaccard index, that is, the ratio of intersection and union, for the markers of all cluster pairs (Supplemental Figure S14). While most clusters do show minor overlap, cluster 32 (root) and cluster 13a (pooled ED/EN) share 82 marker genes (Jaccard-Score 0.31). Both clusters were assigned to cells that undergo division and, thus, we assumed that those genes might be involved in the regulation of the cell cycle. Indeed, 32 out of these 82 genes can be found in the most enriched GO category “cell cycle” (GO:0007049; adj. *P*-value: 7.22*E*−26). Here, the uncharacterized gene “*AT5G16250*” showed the highest FC (i.e. 10.001) in cluster 32 (root) and was more closely investigated (see below).

### Dividing cell marker AT5G16250

**Figure 5:**
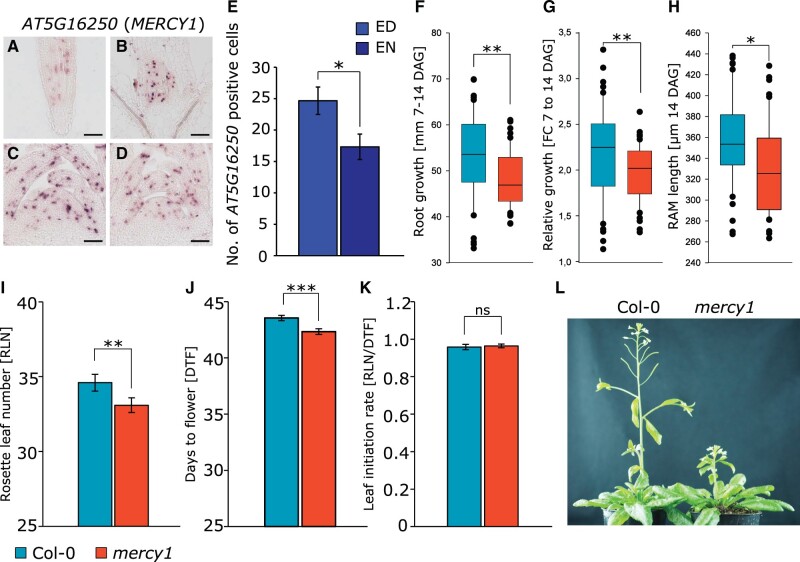
Physiological characterization of dividing cell marker AT5G16250 (*MERCY1*). A–D, RNA in situ hybridization using *AT5G16250* as probe on longitudinal sections through (A) root and (B) SAMs of Col-0 plants harvested at the (C) ED and (D) night (EN). Scale bars equal 100 µm. E, Number of *AT5G16250-*positive cells at the SAM of Col-0 plants at the ED and EN (*n* > 3). F, Absolute root growth, G, Relative root growth, H, RAM length analyzed from 7 till 14 DAG in Col-0 (*n* > 50) and *mercy1* (*n* > 50) mutant plants grown on plates. I–J, Flowering time determined as (I) RLN and (J) DTF. K, LIR. L, Flowering phenotype of Col-0 (*n* > 20) and *mercy1* (*n* > 20) mutant plants. Error bars indicate sd. Statistical difference was calculated using Student’s *t* test (*P < 0.05; **P < 0.01; ***P < 0.001; ns, not significant).

The root and shoot cell cycle clusters are marked by the *AT5G16250* transcript predicted to encode a transmembrane protein. Thus, to confirm its specific expression, we checked the *AT5G16250* expression at different cell cycle stages using CycleBase 3.0 (Santos et al., 2015; Supplemental Figure S15). Indeed, *AT5G16250* expression peaked at the G1 phase of the cell cycle, suggesting that it is involved in the regulation of the cell cycle. Because of this, we named *AT5G16250 MERISTEM CELL CYCLE 1* (*MERCY1*). To confirm the prediction that *MERCY1* is a cell cycle marker involved in the regulation of the cell cycle, we first analyzed the expression of the gene in planta by RNA in situ hybridization on longitudinal sections through root tip and SAM of wild-type plants (Supplemental Methods S6 and Figure  5, A and B).

We found a *MERCY1-*specific signal in the root meristematic zone and the SAM including young leaf primordia. The signal showed a patchy distribution typical for cell cycle regulators (Yang et al., 2017). Moreover, RNA in situ hybridization with cell cycle-specific markers, for example M/G1-phase marker *CYCLIN B1;1* (*CYCB1;1*, AT4G37490) and S-phase marker *HISTONE H4* (*HIS4*, AT2G28740) at the SAM of Col-0 wild-type and *mercy1* mutant plants indicate that knockout of *MERCY1* gene affects the progression of the cell cycle (Supplemental Figure S15). According to our scRNAseq data, *MERCY1* expression was lower at EN than at ED. Also, it is suggested that expression of cell cycle regulators at the SAM is under control of the circadian clock (Masri et al., 2015). Indeed, RNA in situ analysis revealed an increase in the number of *MERCY1*-expressing cells at the meristems harvested at ED compared with those collected at EN (Figure  5, C–E). This observation is in line with our finding in the pooled clustering without ED/EN normalization (Supplemental Tables S16, S17) where expression in dividing ED cells is higher than in EN cells.

We next asked whether *mercy1* KO mutants (SALK_031814C) show a growth phenotype (Supplemental Methods S7). The root analysis revealed that both absolute root growth as well as relative root growth were significantly reduced in the *mercy1* mutant compared with Col-0 plants (Figure  5, F and G). Also, the RAM length was significantly shorter in *mercy1* (*P *<* *0.05) seedlings (Figure  5, H). Next, we made use of a 3D phenotyping system (Apelt et al., 2015) to monitor shoot growth behavior of plants on soil (Supplemental Figure S16). However, we did not find any significant differences between wild-type and *mercy1* regarding the 3D rosette area, relative expansion growth rate (RER), or hyponasty during the vegetative growth phase. Although the final bolt height of *mercy1* plants was substantially shorter compared with Col-0 plants (Figure  5, L), we found that *mercy1* flowered significantly earlier compared with Col-0 based on the RLN (*P *<* *0.01) and DTF (*P *<* *0.001) (Figure  5, I and J). We also observed that the rate of new leaf formation was not affected in *mercy1* in relation to wild-type plants (Figure  5, K).

Taken together, while its exact role is unknown our results indicate that *MERCY1* is involved in meristematic cell division in a regulatory capacity with pleiotropic effects on flowering time, bolting, and root formation.

### Tissue-specific presence of graft-mobile and mono- versus di-cistronic transcripts

**Figure 6:**
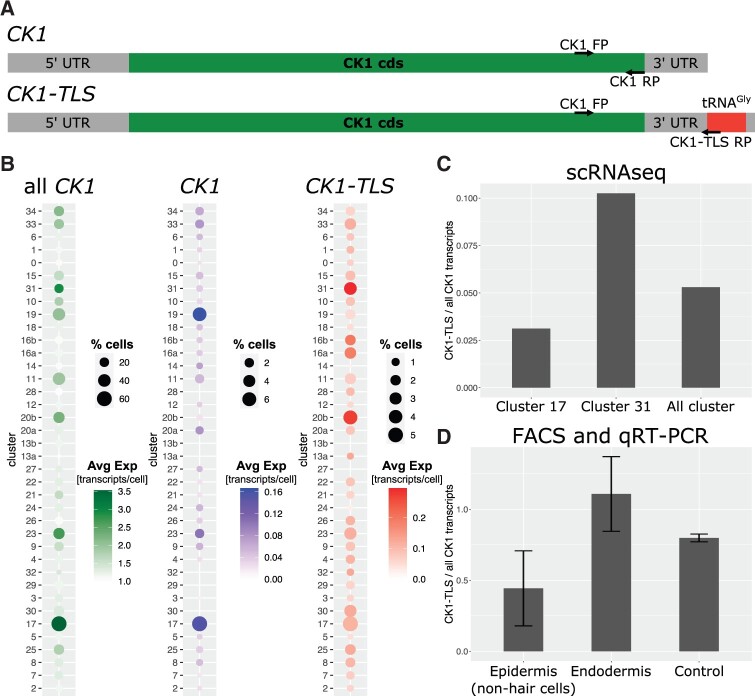
Tissue-specific presence of mono- and di-cistronic *CK1* transcripts. A, Schematic representation of both *CK1* transcripts, that is with and without *tRNA^Gly^* in the 3′UTR named *CK1-TLS* and *CK1*, respectively. Arrows indicate primers (FP, forward primer; RP, reverse primer) used to detect all *CK1* transcripts and specifically *CK1-TLS* transcripts via RT-qPCR (Supplemental Table 1). B, Dot plot of average *CK1* expression and percentage of cells with expression of all *CK1* transcripts, mono-cistronic *CK1* and di-cistronic *CK1-TLS* in root clusters (see Figure  4). Cells with *CK1* expression are considered for calculating the average expression. C, Ratio of *CK1-TLS* compared with all *CK1* transcripts in clusters 17, 31, and all clusters. D, Ratio of *CK1-TLS* compared with all *CK1* transcripts in epidermal cells, endodermal cells, and control cells estimated via FACS and RT-qPCR with specific primers (see “Materials and methods”). Error bars indicate sd calculated with error propagation from *n* = 3 biological samples (each with three technical replicates) for epidermis and endodermis. Control refers to pooled protoplasts without fluorescence signal from all samples (see Supplemental Figure S20).

We also addressed the presence of graft-mobile protein encoding transcripts (Thieme et al., 2015) and their distribution within the clusters. The mobility and distribution of transcripts suggest that they are mainly produced in phloem-associated tissues and allocated via the phloem from shoot to root and unloaded in the protophloem region/phloem pericycle root cells (Zhang et al., 2016, Yang et al., 2019). In line, these graft-mobile transcripts are mainly detected in the developing phloem (20b) and procambium (11 and 19) clusters of roots (Supplemental Figure S17). However, our experimental setup does not allow to distinguish between cells that produce and cells that receive a mobile transcript. Nevertheless, di-cistronic mRNA–tRNA transcripts are significantly enriched among graft-mobile mRNAs (Zhang et al., 2016). To address this class of mRNAs further we identified a total of 1,665 di-cistronic transcripts (comprised of 3,200 unique genes) of which most were derived from adjacent loci (Thimmapuram et al., 2005) using our and publicly available RNAseq datasets (see “Materials and methods”; Supplemental Table S18). Among the clusters with the highest enrichment of di-cistronic transcripts were root clusters annotated as procambium (11/14) and companion cells (24) and shoot clusters (pooled ED/EN) annotated as phloem (12). A total of 10 mRNA–tRNA di-cistronic transcripts were detected in our single-cell samples of which three were detected in all nine scRNAseq samples. One of these is *CHOLINE KINASE1* (*CK1*, AT1G71697) previously shown to be a graft-mobile transcript depending on the presence of the di-cistronic tRNA-like sequence (TLS) (Thieme et al., 2015; Zhang et al., 2016; Figure  6, A). To confirm these predictions, we validated the presence of *CK1* (non-mobile) versus *CK1-TLS* (mobile) by accurately quantifying the two cistronic forms (see “Materials and methods”).

In the root, we found a significant enrichment of di-cistronic *CK1-TLS* transcript compared with mono-cistronic *CK1*, in the procambium cluster 16a (*P*-value 0.031), PPP cluster 20b (*P*-value 0.044) and endodermis cluster 31 (*P*-value 0.027) (Figure  6, B). On average approximately 5% of all *CK1* transcripts were identified as *CK1-TLS* (Supplemental Table S19), whereas this number was twice as high in the endodermis cluster 31 (approx. 10%) and lower in the non-root hair cluster 17 (approx. 3%) (Figure  6, C). Similarly, in the above-ground tissues, *CK1-TLS* was found significantly enriched in mesophyll (cluster 0 for both ED and EN), in epidermis (cluster 23 for ED; clusters 4, 5, 20, and 22 for EN; *P*-value 1*E*−4), and in senescent cells (cluster 8 for ED and cluster 18 for EN) (Supplemental Figure S18).

To confirm this bioinformatic analysis, we measured and compared the transcript levels of *CK1* and *CK1-TLS* in the cell types where the differences were predicted to be high, that is we selected clusters 17 (non-hair epidermis, *CK1* enriched) and 31 (endodermis, *CK1-TLS* enriched). We used the endodermis and non-hair cell marker lines described in Machin et al. (2019) to isolate protoplasts and used FACS to generate relative pure cell type-specific RNA samples for RT-qPCR assays using primers specific for *CK1* and *CK1-TLS* (Figure  6, A;Supplemental Table S1 and Supplemental Methods S8). As a control, cells from the remaining tissues (i.e. the non-fluorescent cells) were collected separately. To avoid a systematic error caused by the difference in size of the PCR products, we created a standard curve using PCR templates of *CK1* (331 bp) and *CK1-TLS* (392 bp) in different ratios and at different concentrations (Supplemental Figure S19). After this evaluation, we submitted the FACS RNA samples to RT-qPCR assays (three technical replicates per biological sample), which confirmed the different ratios of *CK1-TLS:CK1* found by scRNAseq in the two tissues/clusters (Figure  6, D and Supplemental Figure S20). Note that the absolute ratios of *CK1-TLS*:*CK1* were different to those found in the bioinformatic analysis. This deviation seems to be a result of the bioinformatic approach in which we excluded reads that did not cover unique *CK1*-*TLS* di-cistronic sequences, which most likely underestimates the true quantity of *CK1-TLS* mRNA. Despite this, the relative *CK1-TLS:CK1* ratios were similar to those predicted by the analysis of the scRNAseq dataset, in that *CK1-TLS* was found to be enriched compared with *CK1* in the endodermis and was found in smaller quantities than *CK1* in the non-hair epidermis.

### Non-annotated transcripts with tissue-specific expression

**Figure 7:**
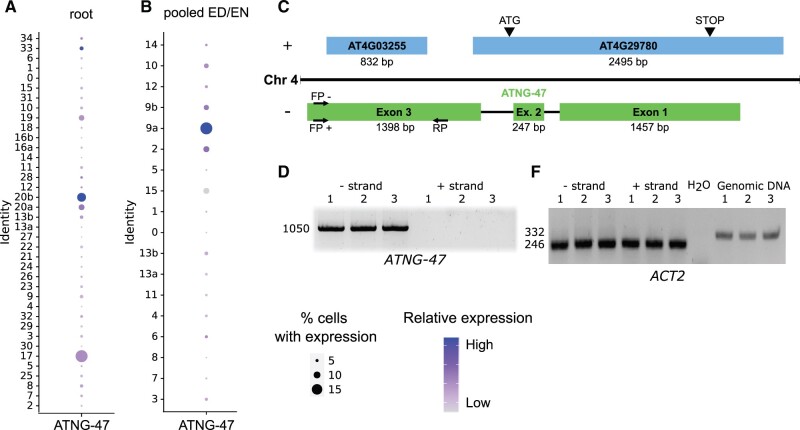
Non-annotated locus (ATNG-47) with tissue-specific expression. A, Dot plot of expression of *ATNG-47* transcript in root (see Figure  4) and B, rosette (pooled ED/EN) clusters (see Figure  2). The color scale denotes the relative expression (average per cell). The dot size is scaled to the proportion of cells per cluster with *ATNG-47* expression. C, Schematic representation of *ATNG-47* transcript located on chromosome 4 on the negative strand, partially overlapping with *AT4G29780* and *AT4G03255* on the positive strand. Arrows indicate strand-specific primers (FP, forward primer; RP, reverse primer) used to detect *ATNG-47*. D, ATNG-47 amplification (1,050 bp region in exon 3) using negative strand-specific cDNA library. E, *ACTIN2* (*ACT2)* primers spanning an intron so that cDNA PCR results in a 246 bp band and gDNA results in a 332 bp band. 246 bp band in the cDNA samples confirms gDNA absence in cDNA libraries.

The recent scRNAseq data release focus on annotated RNAs and analyzed their functionality as marker transcripts. This potentially misses the opportunity to discover rare or unidentified non-annotated transcripts with a cell type-specific function. Thus, we re-annotated the Arabidopsis transcriptome to expand the existing annotations to include 9,421 non-annotated transcripts (see “Materials and methods” for details). Out of these, 9,228 transcripts were predicted as additional isoforms of the existing genes. The remaining 193 predicted transcripts were present in previously non-annotated regions covering 125 non-annotated genes (Supplemental Table S3). Based on coding potential (Kang et al., 2017), 45 of these transcripts were predicted as protein-coding and 148 as non-coding. In this scRNAseq analysis, we found four non-annotated loci (*ATNG-18*, *ATNG-47*, *ATNG-98*, and *ATNG-99*) that were also markers for both root and shoot clusters (Supplemental Tables S10, S13). From these, *ATNG-47* was the main marker transcript for root PPP; (cluster 20b) and EN/ED shoot (senescent cells/parenchyma; clusters 2 and 9a) (Figure  7, A and B). *ATNG-47* is an antisense transcript partially overlapping with the *AT4G29780* and *AT4G03255* of unknown function (Figure  7, C). To confirm this bioinformatic prediction, two cDNAs were produced by using positive and negative strand-specific *ATNG47* RT*-*primers for cDNA synthesis (Figure  7, D and Supplemental Methods S8). To confirm that the detected amplicon was not produced by a genomic DNA contamination, we used *ACTIN2*-specific primers (Figure  7, E). These controls and the strand-specific RT-PCR assays confirmed the antisense nature and expression of the *ATNG-47* transcript (Figure  7, D). In summary, this reveals that non-annotated and tissue-specific transcripts can be identified by scRNAseq that cannot be identified by conventional transcriptomic approaches which are potentially interesting for further studies.

## Discussion

To implement Drop-seq as a reliable scRNAseq method, we had to adopt established root and shoot protoplasting protocols to produce debris-free single-cell suspensions at ED and EN timepoints. These changes allowed us to successfully produce high-resolution single-cell transcriptomic maps of nearly 70,000 Arabidopsis cells for (i) roots of 7-d-old seedlings at ED and for (ii) above-ground tissue of 5-week-old plants at two distinct times of the day (ED and EN). We produced reference RNAseq libraries from the respective tissue by using the same cDNA protocol and RT primer employed for the scRNAseq library production. We observed a high reproducibility across replicates (*r* > 0.9) and a high correlation between scRNAseq libraries and corresponding reference RNAseq libraries (*r* approx. 0.8), which is comparable to recently published datasets (Denyer et al., 2019; Shulse et al., 2019). However, we find a large proportion of DEGs between scRNAseq and reference RNAseq datasets, which were reproducible across tissues and time points, and which were also identified in other studies using protoplasts (Birnbaum et al., 2003; Denyer et al., 2019; Kim et al., 2021). The fact that the aforementioned independent studies also report similarly extensive and overlapping transcriptomic differences between protoplasted and reference tissue underlines that this issue should be taken into consideration when working with scRNAseq. This is highlighted by the fact that the harvesting time point of shoot cells (ED or EN) is less distant to each other than the reference RNAseq to scRNAseq indicating the importance of creating and analyzing suitable reference libraries.

As proposed by Efroni and Birnbaum (2016), the new single-cell RNAseq technologies provide a more unbiased approach to the existing single-cell profiling methods, for example cell sorting (FACS), that use markers with clear anatomical specificity. In our study, we show that certain cell types are characterized by markers that do not show a distinct spatio-temporal expression but are rather related to developmental stage/cell cycle phase.

An important point is that the full potential of scRNAseq is still being explored. Recent publications have already highlighted the value of this method for studying mutant phenotypes (Ryu et al., 2019), environmental effects like heat shock (Jean-Baptiste et al., 2019), and mobile RNAs (Saplaoura and Kragler, 2016). In this study, we address the effect of the time of the day on the transcriptomes of different cell types and further investigate the cell-specific presence of alternative transcript versions (mono- and di-cistronic).

Among the various single-cell studies of Arabidopsis, from similar numbers of cells analyzed, different numbers of clusters have been identified ranging from as few as 8 to as many as 24 (Zhang et al., 2019) depending on the method (10× Chromium or Drop-seq) and especially the parameters applied for the downstream analysis. Our approach has resulted in the separation of 35 clusters from the primary root representing different developmental stages of cells as well as some highly specific cell types such as procambium, endodermis, cortex, hair cells, non-hair cells, xylem and phloem pericycle cells, and CCs. However, it is important to mention that our average number of transcripts/genes per cell are lower than in other published studies due to lower applied minimum cutoffs to increase the number of cells analyzed (see above). The cell types that were not identified in our dataset are the QC and columella cells. Our study addresses the transcription profile of whole above-ground tissue of Arabidopsis plants grown on soil harvested at two different times of the day (ED and EN). Kim et al. (2021) focused on the vascular cells of the leaves and Zhang et al. (2021) on the shoot apex. Despite the enrichment of vascular cells reported by Kim et al. (2021), 11 out of 19 clusters were classified as mesophyll cells. This is in agreement with our cluster annotations according to which 5 of 16 clusters of the ED/EN pooled analysis were mesophyll or senescent cells. Overall, the complexity in the combined dataset (16 clusters) seemed to be lower than in the individual datasets (ED: 37, EN: 25) represented by a lower number of clusters. This is a result of merging two scRNAseq datasets whose global differences are dominated by the time point of harvest, which leads to a loss of complexity within tissues. For example, at ED 10 different clusters are annotated as mesophyll cells, while only four at EN and three in the pooled ED/EN clustering. This is not surprising when considering that mesophyll cells are photosynthetically active and, thus, a higher complexity in their transcriptome is expected at ED. Nevertheless, the cluster robustness analysis revealed that the clusters in the individual ED and EN clustering are not as robust as in the combined ED/EN clustering. Analyzing the enrichment of MapMan functional categories within the clusters also enabled us to perform deeper functional discoveries, that is we found starch metabolism-related genes enriched within mesophyll cells. The starch metabolism has been extensively studied in Arabidopsis where it has a linear pattern of starch synthesis in the light, peaking toward the ED and starch degradation in the night with little starch left at the EN (Stitt and Zeeman, 2012; Smith and Zeeman, 2020). From published experiments performed on whole Arabidopsis rosettes, transcripts encoding many of the enzymes involved in starch metabolism were shown to undergo large diel fluctuations, peaking at the ED independently if they encode starch synthesis or starch degradation enzymes (Smith et al., 2004; Smith and Zeeman, 2020). Our study using single cells provides details on the differential transcript enrichment at ED and EN. Our finding of a higher enrichment of starch biosynthesis transcripts at EN compared with ED could be interpreted as a diurnal anticipation step of mesophyll cells for the upcoming light period that will require starch synthesis enzymes. The greater enrichment of starch degradation genes at ED compared with EN is in line with starch degradation that starts working at ED.

For above-ground tissues, an additional clustering approach of pooled ED/EN cells without normalizing for the global ED versus EN differences enabled us to address cell type-specific transcriptome changes, which is a valuable resource for further investigations.

We also showed that scRNAseq can be used to uncover the function of non-characterized gene/transcripts of which *MERCY1* (AT5G16250) was experimentally confirmed regarding its role in growth and development. *MERCY1* was identified as a cell-cycle-related marker transcript in both shoot and root based on its specific presence in clusters identified as dividing cells. It is well-established that plant growth and development depends on the interplay between meristematic cell division and post-meristem cell expansion (Green, 1976). In line, primary root growth and onset of bolting are affected but not cell expansion-driven growth of rosettes in *mercy1* mutants.

Clusters with unknown identity were reported for the root and shoot in our and in most published scRNAseq datasets. This underlines the fact that knowledge of the cell types transcriptomic profiles is still incomplete. Some of such non-annotated clusters might also be a product of technical or computational artifacts or both, such as the presence of two different cells in one droplet barcoded by one bead or RNA leakage during droplet formation. Finally, a recent review raised the issue that existing scRNAseq bioinformatic pipelines lack functions to address the large cell-size variability and whether this can cause bias in the quantification of transcriptional activity (Shaw et al., 2020). Irrespective of these potential pitfalls, unknown clusters, as well as clusters with interesting transcriptomic profiles, can lead to the identification of new cell types and facilitate the in-depth characterization of neglected cell types, respectively. One such particularly interesting example is that found with cluster 10 of the pooled ED and EN cells (#25 in the ED and #9 in the EN cells). The presence of this specific cluster(s) characterized by transcripts encoding proteins involved in plant defense presents an opportunity to further study the identity and function of this predicted cell type. On the other hand, it is possible that certain parenchyma cells differentiate into specialized cells (idioblasts) that secrete defense-related proteins like the PRs (pathogenesis-related proteins) into the apoplast (Uknes et al., 1992). The study of development and mechanism of differentiation of such predicted functions of cell type sub-populations will expand our understanding of the defense mechanism(s) of plants in a cell type-specific context.

High-quality genome annotations are crucial for extracting meaningful insights into the biological aspects of the species of interest. Here, we re-annotated Arabidopsis genome to include non-annotated isoforms and loci based on transcript-based evidence, and predicted di-cistronic transcripts at high resolution. Based on this, we identified a non-annotated transcript (*ATNG-47*) preferentially expressed in distinct cell types suggesting the potential importance of such loci in the Arabidopsis genome. Furthermore, we exploited the power of scRNAseq to go beyond the “traditional” scRNAseq analysis and look at the individual transcripts from the sequence level. Using this approach, we investigated the distribution of di-cistronic transcripts across the cell types and separately quantified the mono- and di-cistronic (*CK1-TLS*) versions of the *CK1* transcript. We experimentally confirmed the enrichment of *CK1-TLS* within the endodermis via FACS and RT-qPCR. Beside the endodermis, the graft-mobile *CK1-TLS* (Thieme et al., 2015) was also enriched in PPP, which is in line with the hypothesis that PPP cells are the primary location of unloading of signaling molecules (Ross-Elliott et al., 2017; Yang et al., 2019) from the phloem and may act as a gateway that controls the transit of mobile RNAs and restricts movement to specific molecules only.

## Conclusions

In conclusion, we have created a high-resolution single-cell transcriptomic map of Arabidopsis root at ED and above-ground tissues at ED and EN and identified tissues-specific markers for both time points. We found that depending on the time of the day, single-cell transcriptome changes occur in distinct tissues to variant degree. Interestingly, we observed that the most similar tissue type between root and above-ground tissue is dividing cells. This observation led us to investigate a previously uncharacterized marker of that cluster (*MERCY1*) and demonstrate its role in meristematic growth. Furthermore, we identified non-annotated transcripts that seem to serve as tissue-specific markers and analyzed the differential presence of mono- and di-cistronic transcripts within tissues.

## Materials and methods

### Plant material, growth condition, and protoplast isolation for primary roots

For the root samples, three Arabidopsis (*A.* *thaliana*) estradiol-inducible fluorescent marker lines (Columbia-0, Col-0 background) with characterized expression patterns were used to produce scRNAseq libraries of the root using the Drop-seq protocol. Lines V161 and V171 express *H2B-Venus* in the PPP and the XPP under the promoter of *AT5G09760* and *AT3G29635*, respectively. Line V311 expresses *H2B-Venus* in differentiated cells under the promoter of *TCP DOMAIN PROTEIN 7* (*TCP7*, AT5G23280) (Machin et al., 2019). The plants were grown on tissue cultures and the entire root was harvested 7 d after germination (DAG) and used for protoplast preparation. All three lines were grown and harvested independently at the same timepoint (ED) on different days (*n* = 5 plates with four rows of vertically grown seedlings). Each line comprises an independent experiment and were considered replicates (Supplemental Figure S1; for details, see Supplemental Methods S1).

### Plant material, growth condition, and protoplast isolation for above-ground tissue

Col-0 seeds were sown on soil (Stender; www.stender.de) mixed with vermiculite (1:1) soaked with tap water, supplemented with boron (1.8 mg L^−1^) and the fungicide Previcur (1.5 mL L^−1^; Bayer; www.bayer.de) and germinated in 12-h light/dark photoperiod (NDs) at 20°C in controlled environmental chambers (160 μE m^−2^ s^−1^). After 1 week, the seedlings were transferred to an 8-/16-h light/dark photoperiod (short days, SDs) at 20°C/16°C for 1 week. Then plants were transferred into individual pots (five plants per pot) and grown under ND (12-h light/12-h dark) photoperiod with 22°C/18°C temperature (Percival E-36 L chamber, CLF Plant Climatics, Wertingen, Germany). Four weeks after transfer, all above-ground tissue was used for protoplast preparation (Supplemental Figure S1; for details, see Supplemental Methods S1). For each timepoint (ED or EN), three biological replicates were harvested (*n* = 40 plants) and submitted to scRNAseq. Each of the three library pairs ED1/EN1, ED2/EN2, and ED3/EN3 was harvested within 24 h from the same batch of plants (Supplemental Figure S1; for details, see Supplemental Methods S1).

### ScRNAseq with Drop-seq

A modified Drop-seq scRNAseq protocol was used based on the description provided by Macosko et al. (2015) using single beads linked with unique barcoded poly-T oligonucleotides that are loaded with individual protoplasts into droplets (Supplemental Figure S1; for details, see Supplemental Methods S2). For an overview of the sequenced libraries, see Supplemental Table S2.

### Reference tissue RNAseq

The reference tissue cDNA libraries were produced from total RNA extracted from roots (one plate for each genotype) and above-ground tissue from soil grown plants (20 plants) harvested at the same time points as for the scRNAseq libraries and, to avoid a potential preparation bias introduced by PCR amplification/tagmentation, the reference tissue cDNA libraries were prepared in the same way as the scRNAseq cDNA libraries (Supplemental Methods S3). For an overview of the sequenced libraries, see Supplemental Table S2.

### RNA in situ hybridization

Root and shoot tissues of 7-d-old wild-type Col-0 plants, grown in ND conditions, were harvested at the ED. Fixation, embedding, sectioning, and RNA in situ hybridization were carried out as previously described (Olas et al., 2021) (for details, see Supplemental Methods S6).

### Growth and morphology analysis

For root growth analysis, sterilized seeds were germinated in vertical positioned 10% w/v agar 0.5× MS 0.6% w/v sucrose plates in 16-h light/dark photoperiod (long days, LDs). At 7 DAG, the seedlings were transferred to fresh 10% w/v agar 0.5× MS 0.6% w/v sucrose plates (for details, see Supplemental Methods S7).

For shoot growth analysis, we used an established 3D phenotyping system (Apelt et al., 2015) for imaging size, RER, and leaf angle (hyponasty). Plants were grown as previously described (Apelt et al., 2015). The flowering time was defined as “days to flower” (DTF) corresponding to the day on which the main stem has bolted 0.5 cm, and as “rosette leaf number” (RLN). The leaf initiation rate (LIR) was determined by calculating the ratio of the RLN and DTF. At least 20 genetically identical replicate plants were used to determine the growth behavior and flowering time of each genotype. Student’s *t* test was used to test the significance of flowering time differences.

### FACS of root protoplasts

Seedlings of fluorescent marker lines V141 and V101 marking the endodermis and non-hair cell epidermis, respectively (Machin et al., 2019), were grown and protoplasts harvested as described above. Fluorescent cells were sorted using a BD FACS Aria II machine using a 100-µm nozzle. 15,000 Venus+ cells were sorted from each line, and 15,000 unlabeled cells were also collected as a control. Protoplasts were sorted into 1× TE buffer with RNAse inhibitor and immediately frozen in liquid nitrogen.

Total RNA was extracted using phenol:chloroform:isoamyl alcohol and then pelleted with 5-M LiCl mixed to a final volume of 2.5 M and linear acrylamide to a final concentration of 20 µg/mL. cDNA was subsequently made by reverse transcription with Reverse Transcriptase (Promega) using poly-T primers and used for RT-qPCR.

### RT-qPCR of *CK1*/*CK1-TLS* and non-annotated transcript

For details, see Supplemental Methods S8.

### Computational analysis

#### Annotation of Arabidopsis reference genome and alignment

A comprehensive Arabidopsis reference genome annotation was constructed by updating the Araport11 Arabidopsis genome annotations (Cheng et al., 2017) with annotations from RepTAS (Liu et al., 2012) and miRBase (Kozomara et al., 2019). Further, non-annotated loci and transcripts were identified using publicly available high-depth RNAseq datasets from NCBI SRA (SRX853394, SRX853395; Thieme et al., 2015; DRX014481, DRX014482; Ito et al., 2015; Supplemental Table S3). The scRNAseq and reference RNAseq samples were processed using the Drop-seq computational pipeline (release 2.3.0; https://github.com/broadinstitute/Drop-seq/). The final alignment files were used for quantifying expression of all genes using htseq (version 0.12.4). The raw counts were further normalized using DESeq2 (for details, see Supplemental Methods S9).

#### DE, PCA, and enrichment analysis

The DE analysis was performed using DESeq2 with a significant threshold (FDR) of 0.05 and |log_2_FC| ≥ 1. The detailed pairwise comparisons for DE are listed in Supplemental Table S5. PCA was performed using “prcomp” function in R. The Pearson’s correlation between Drop-seq and respective tissue samples was calculated by comparing the replicate-averaged log_2_ DESeq normalized expression using “cor” function in R. The correlation values were plotted using ggplot2. GO enrichment analysis was performed using a binomial test and Bonferroni corrected *P*-value < 0.05 was considered significant. GO annotations were obtained from PANTHER database (Thomas et al., 2003). We downloaded MapMan4 annotations from Mercator4 (Schwacke et al., 2019) and performed enrichment test using hypergeometric test in R with *P*-value threshold of 0.05.

#### Clustering, cluster robustness, and marker identification for single-cell samples

The digital expression matrices obtained from Drop-seq pipeline were analyzed using Seurat v3.0.2 (Stuart et al., 2019) and Harmony v0.99.9 (Korsunsky et al., 2018) using custom parameters. Cluster robustness (0–100%) was scored for each clustering from 100 random subsets of 80% of the library reads and describes what proportion cells that previously formed a cluster are still co-occurring in a single cluster when using subsampled data for clustering while retaining the variable genes from the original clustering. Cluster markers were calculated using FindAllMarkers function of Seurat (fold change ≥ 0.25, minimum cell percentage ≥ 5%, and adjusted *P*-value < 0.05) (for details, see Supplemental Methods S5).

#### Prediction of di-cistronic transcripts

The alignments from the four samples were used to predict di-cistronic transcripts. A read was considered to originate from two genes if the alignment coordinates overlap with two annotated genes (Araport11 annotations only). A gene pair was considered to encode a di-cistronic transcript if it was supported by ≥100 reads. These gene pairs were further filtered to retain pairs if both genes are protein-coding and present on the same strand; or at least one gene is non-coding.

#### scRNAseq analysis of CK1 and CK1-TLS

The mono-cistronic *CK1* (*CK1-mono*) and di-cistronic *CK1* (*CK1-TLS*) were quantified from the single-cell RNAseq data with custom-made scripts. A transcript was annotated as *CK1-mono* if no reads from transcript overlap the tRNA region (AT1G71700) and the reads end at 1 bp before the start of the tRNA, and a transcript was annotated as *CK1-TLS* if at least one read from the transcript aligned to the tRNA region. The dot plots were plotted using ggplot2 package in R. Clusters were considered to be enriched in *CK1-TLS* if *CK1-TLS* was identified as a marker for the cluster (two-tailed Wilcoxon rank sum test; *P*-value < 0.05) and *CK1*-*mono* was not.

## Data availability

The sequencing data sets are available at the NCBI Sequencing Read Archive (SRA), BioProject ID PRJNA742744.

## Accession numbers

Sequence data from this article can be found in the GenBank/EMBL data libraries under accession numbers: MERCY1 (AT5G16250) and CK1 (AT1G71697).

## Supplemental data

**Supplemental Figure S1.** Outline of the experimental workflow.

**Supplemental Figure S2.** RT-PCR and normalized expression of marker genes in the single-cell and reference tissues of root and above-ground samples.

**Supplemental Figure S3.** PCA, clustering, and correlations.

**Supplemental Figure S4.** DEGs affected by scRNAseq compared with published data sets.

**Supplemental Figure S5.** Single-cell transcriptome of root and above-ground samples prior filtering.

**Supplemental Figure S6.** *t*-SNE projection with cell colors for replicates.

**Supplemental Figure S7.** Analysis of cluster robustness.

**Supplemental Figure S8.** Enrichment of cluster marker genes in the MapMan functional categories.

**Supplemental Figure S9.** Above-ground cluster (pooled ED/EN) enrichment for MapMan categories related to starch metabolism.

**Supplemental Figure S10.** Enrichment of MapMan level 1 categories in above-ground samples.

**Supplemental Figure S11.** Marker genes in above-ground clusters.

**Supplemental Figure S12.** Root cap enriched MapMan categories related to secondary metabolism.

**Supplemental Figure S13.** MapMan category “chromatin organization” enriched in root and above-ground clusters related to RAM and SAM, respectively.

**Supplemental Figure S14.** Overlap of markers between root and above-ground (pooled ED/EN) clusters.

**Supplemental Figure S15.** Characterization of molecular function of *AT5G16250* (*MERCY1*).

**Supplemental Figure S16.** Analyzing growth behavior of *mercy1*.

**Supplemental Figure S17.** Enrichment of graft-mobile annotated transcripts and di-cistronic transcripts in root and shoot (pooled ED/EN) clusters.

**Supplemental Figure S18.** Dot plots representing transcript accumulation of *CK1*, mono-cistronic *CK1* and *CK1-TLS* transcripts for each cluster in above-ground clusterings.

**Supplemental Figure S19.** RT-qPCR of standard curves created using purified PCR products of *CK1* and *CK1-TLS*.

**Supplemental Figure S20.** FACS of tissue-specific protoplasts and RT-qPCR of isolated cDNA.

**Supplemental Table S1**. List of primer sequences used.

**Supplemental Table S2.** Summary of bulk and single-cell libraries used in the study.

**Supplemental Table S3.** List of all predicted non-annotated transcripts and loci.

**Supplemental Table S4.** List of differentially expressed genes.

**Supplemental Table S5.** GO enrichment analysis of DEGs between ED and EN.

**Supplemental Table S6.** Summary of clusters for root samples.

**Supplemental Table S7.** Summary of clusters for end of day samples.

**Supplemental Table S8.** Summary of clusters for end of night samples.

**Supplemental Table S9.** Summary of clusters for pooled end of day + end of night samples.

**Supplemental Table S10.** List of markers identified for root clusters.

**Supplemental Table S11.** List of markers identified for end of day clusters.

**Supplemental Table S12.** List of markers identified for end of night clusters.

**Supplemental Table S13.** List of markers identified for pooled end of day + end of night clusters.

**Supplemental Table S14.** Comparison of individual end of day and end of night clusters with pooled end of day + end of night clusters.

**Supplemental Table S15.** List of markers identified for pooled end of day + end of night clusters without normalization.

**Supplemental Table S16.** Comparison of pooled end of day + end of night clusters with and without normalization.

**Supplemental Table S17.** List of markers identified between specific clusters for pooled end of day + end of night clusters without normalization.

**Supplemental Table S18.** List of all predicted di-cistronic transcript pairs.

**Supplemental Table S19.** Summary of *CK1* and *CK1-TLS* transcripts identified in the root clusters.

**Supplemental Table S20.** Maximum percentage of cells for the dot plots for all clusters.

**Supplemental Methods S1.** Protoplast isolation.

**Supplemental Methods S2.** ScRNAseq with Drop-seq.

**Supplemental Methods S3.** Reference tissue RNAseq.

**Supplemental Methods S4.** Quality control PCRs.

**Supplemental Methods S5.** Clustering and marker identification for single-cell samples.

**Supplemental Methods S6.** RNA in situ hybridization.

**Supplemental Methods S7.** Growth and morphology analysis.

**Supplemental Methods S8.** RT-qPCR of *CK1*/*CK1-TLS* and non-annotated transcript.

**Supplemental Methods S9.** Annotation of Arabidopsis reference genome and alignment.

**Supplemental Text S1**. MapMan annotation of protoplast-induced genes.

**Supplemental Text S2**. ED cluster annotation.

**Supplemental Text S3.** EN cluster annotation.

**Supplemental Text S4.** ED/EN cluster annotation.

**Supplemental Text S5**. Root cluster annotation.

**Supplemental Text S6**. Root cap and secondary metabolism.

## Supplementary Material

kiab537_Supplementary_Data

## Notes

Senior author.

These authors contributed equally to this work (F.A., E.M.).

E.M. performed the Drop-seq and PCR experiments. F.A. and S.G. analyzed the single-cell transcriptomics data. S.G. performed the differential expression, di-cistronic, and the non-annotated transcript analyses. F.M. analyzed the cluster identity, performed root growth analyses, and FACS/RT-qPCR experiments supported by D.S. J.J.O. performed RNA in situ hybridizations. F.A. performed the shoot growth analysis, and generated the figures and wrote the manuscript with F.K. and contributions from all authors. M.G.A. assisted with the above-ground harvesting and interpretation of the results. F.K. conceived the idea, suggested the experiments and analyses of data, and supervised the project. All authors read the manuscript and expressed their consent to the manuscript.

The author responsible for distribution of materials integral to the findings presented in this article in accordance with the policy described in the Instructions for Authors (https://academic.oup.com/plphys/pages/general-instructions) is: Friedrich Kragler (kragler@mpimp-golm.mpg.de).
