# Supplementary material for: Shoot and root single cell sequencing reveals tissue- and daytime-specific transcriptome profiles
Source: Plant Physiol. 2021 Nov 19;188(2):861–78. doi: 10.1093/plphys/kiab537 (PMC8825464; doi:10.1093/plphys/kiab537)
Supplement: kiab537_Supplementary_Data [file kiab537_supplementary_data.zip › scManuscript_SupplementalData.pdf]

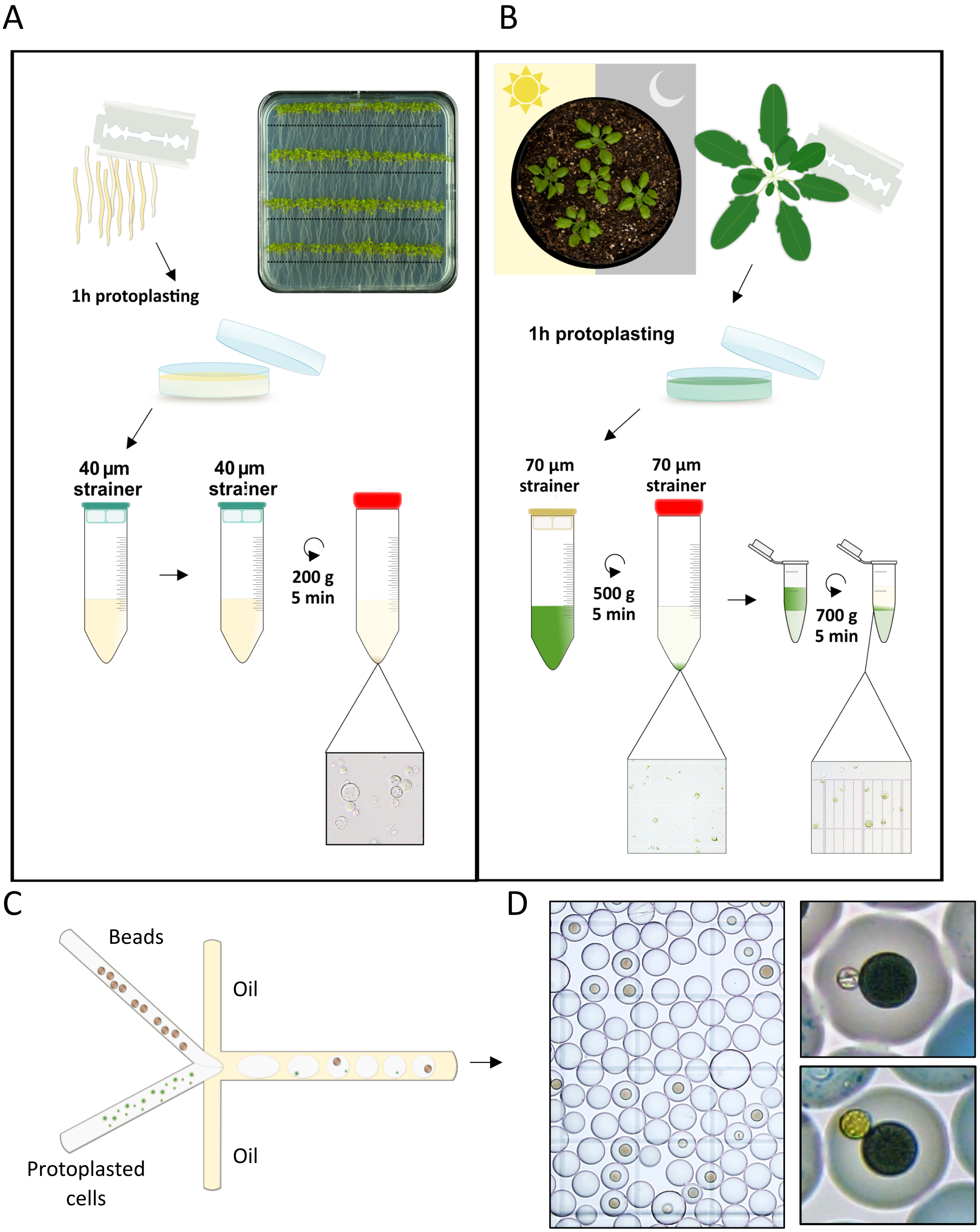

Supplemental Figure S1. Outline of the experimental workflow. A, The plant root tissue is harvested approximately 1 cm below the hypocotyl (indicate by the black line) and protoplasted for 1 h. The root protoplasts were collected at the end of the day (ED), filtered through a 40 µm cell strainer twice, and centrifuged at 200 g for 5 min. **B**, The rosette protoplasts are collected at the ED and end of the night (EN), filtered through a 70 µm cell strainer, centrifuged at 500 g for 5 min and purified with a second centrifugation at 700 g for 5 min over a 13.3% Ficoll-400 cushion. **C**, The protoplasts and barcoded beads flow through a microfluidics chip and, **D**, are co-encapsulated in aqueous droplets with an oil surface; grid size = 250 µm.

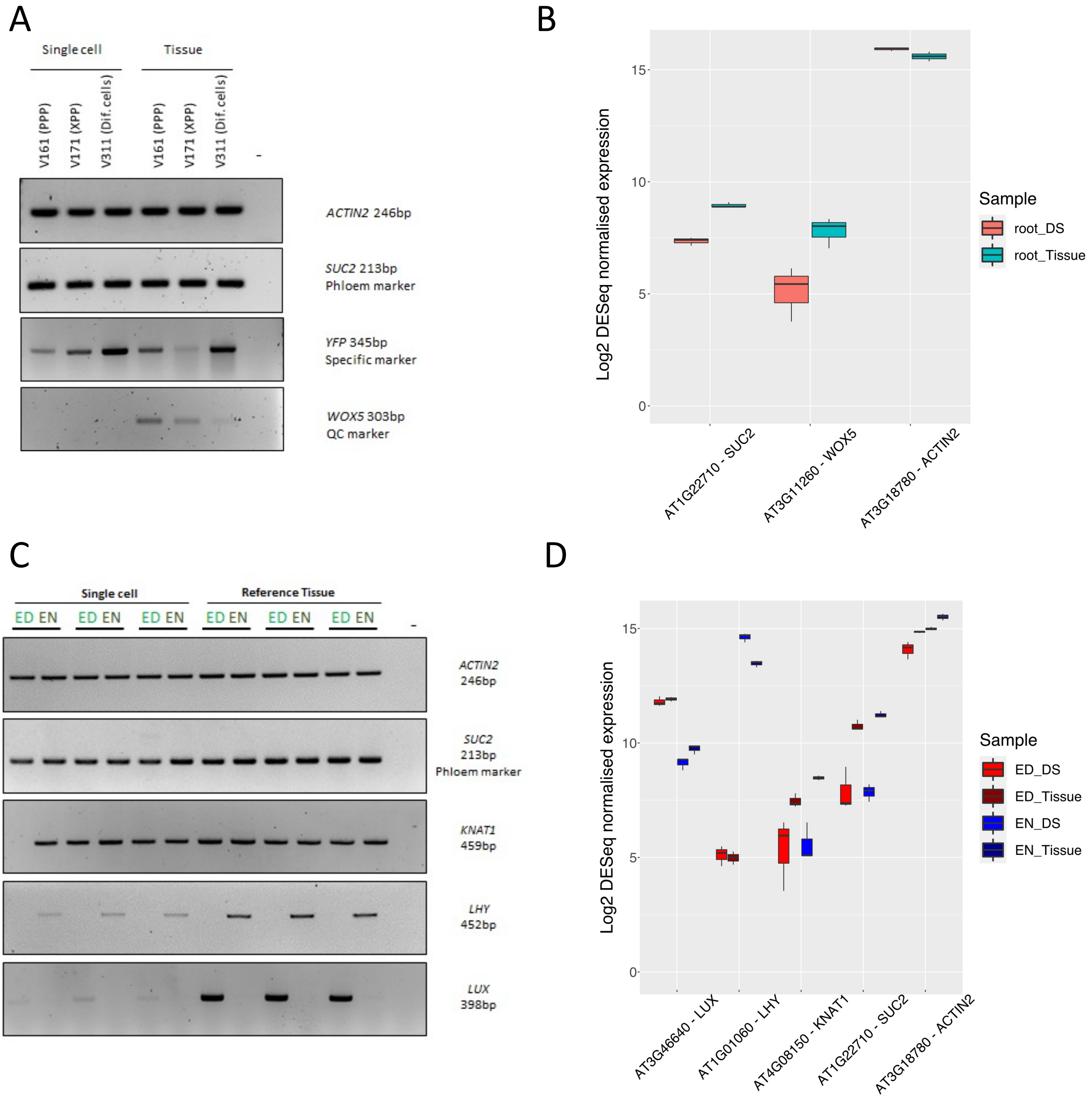

Supplemental Figure S2. RT-PCR and normalized expression of marker genes in the single-cell and reference tissues of root and above-ground samples. A, The presence of transcripts of interest was tested by PCR amplification for 35 cycles, 1 µl of the cDNA libraries of the single-cell (first three lanes), as well as the reference tissue samples. The last lane is water as negative control. Note that all PCR amplicons had the expected size and the *ACTIN2* primers do not amplify the 332 bp gDNA band. **B**, Normalized expression (Log_2) of *SUCROSE-PROTON SYMPORTER 2 (SUC2), WUSCHEL RELATED HOMEOBOX 5 (WOX5),* and *ACTIN2* analyzed in root samples using scRNAseq (DS; red; n=3 libraries) and reference RNAseq (tissue; green; n=3 libraries). **C**, The presence of transcripts of interest was tested by PCR amplification of the cDNA libraries of the single-cell as well as the reference tissue samples of end of day (ED) and night (EN) periods. *ACTIN2*, *SUC2*, and *KNOTTED1-LIKE HOMEBOX (KNAT1)* were amplified for 35 cycles and *LATE ELONGATED HYPOCOTYL* (*LHY*) and *LUX* were amplified for 25 cycles. **D**, Normalized expression (Log_2) of *LUX, LHY, KNAT1, SUC2*, and *ACTIN2* analyzed in shoot samples harvested at the end of the day (ED) and night (EN) periods using DropSeq (DS; n=3 libraries at ED and n=3 libraries at EN) and reference (tissue; n=3 libraries at ED and n=3 libraries at EN).

Boxplots (B,D): center line, median; box limits, upper and lower quartiles; whiskers, 1.5x interquartile range.

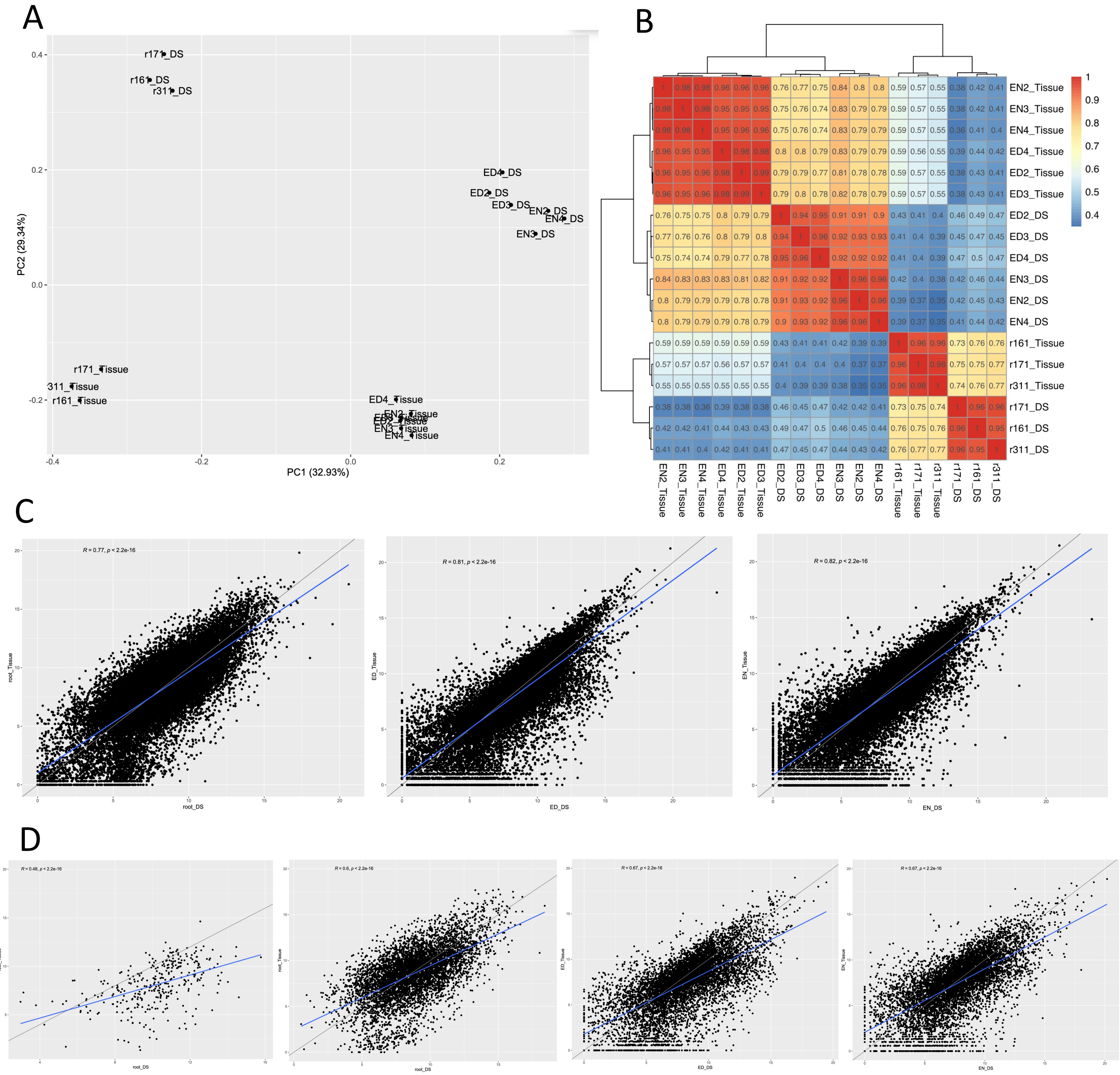

Supplemental Figure S3. PCA, clustering and correlations. A, Principal component analyis (PCA) of the sequenced scRNAseq and reference RNAseq libraries. **B**, Hierarchical clustering with detailed correlation values of the sequenced scRNAseq and reference RNAseq libraries using Pearson's correlation coefficient (r) as distance measure. **C**, Correlation between scRNAseq and reference RNAseq libraries for root (left), rosette at the end of the day (ED; middle) and end of the night (EN; right). **D**, Correlation between scRNAseq and reference RNAseq for genes previously described to be protoplast induced (Birnbaum *et al.,* 2013, 346 genes, first panel) or differentially expressed upon protoplasting roots in roots (Denyer *et al.,* 2019, 6063 genes, second panel) and shoots (Kim *et al.,* 2020, 8845 genes) at ED (third panel) and EN (fourth panel).

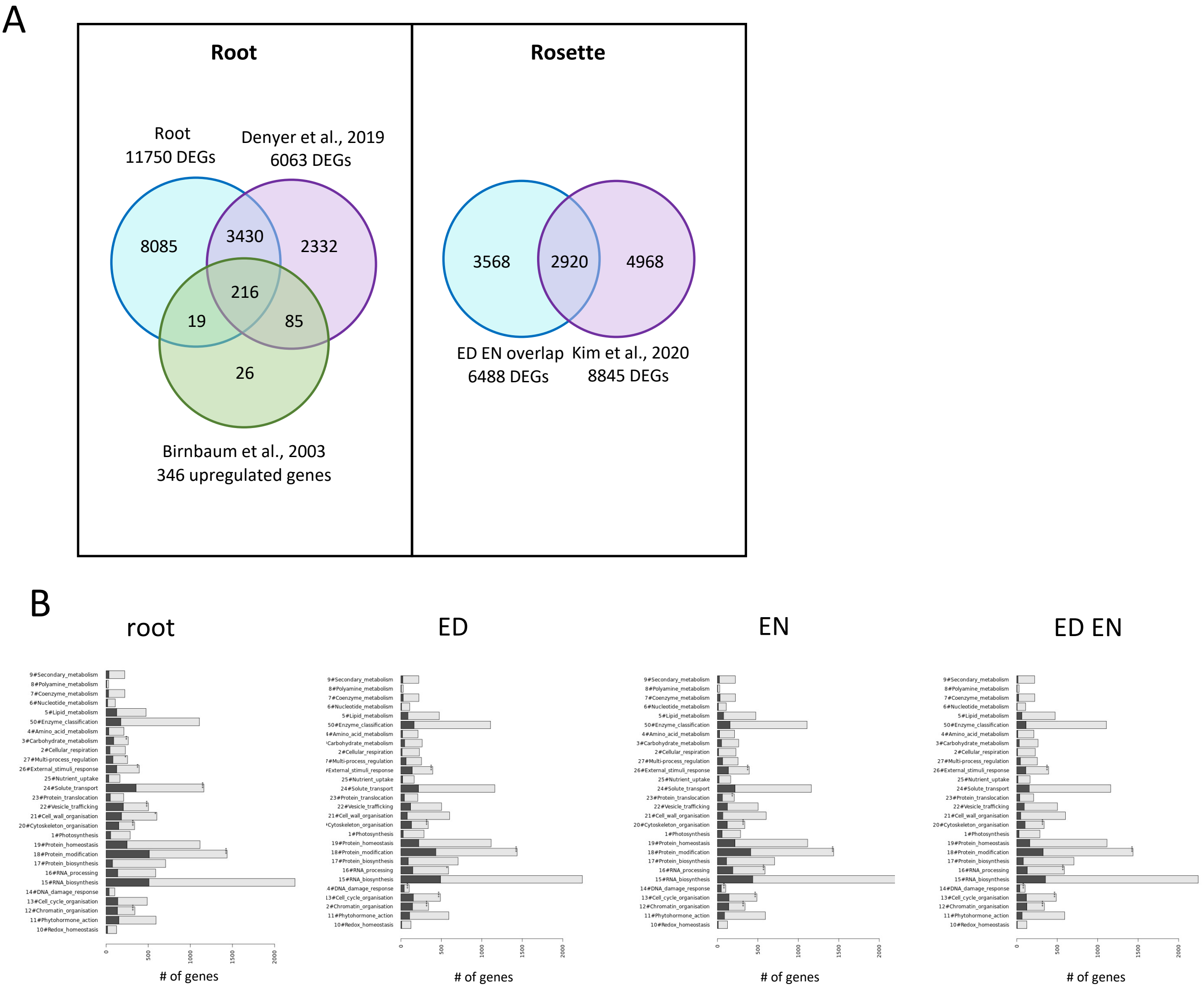

Supplemental Figure S4. DEGs affected by scRNAseq compared to published data-sets. A, Venn Diagram of DEGs between scRNAseq and reference RNAseq in our root samples (left panel) overlap significantly with the DEGs identified in root protoplasts by Denyer *et al.,* 2019 (*P*-value 0). Both sets of DEGs contain the majority of the protoplast-upregulated genes found by Birnbaum *et al.,* 2003. The rosette DEGs between scRNAseq and reference RNAseq (right panel) common between end of the day (ED) and end of the night (EN) (6488) overlap significant with the published set of leaf DEGs by Kim *et al.,* 2020 (*P*-value < 9.094e-293). **B**, Enrichment of protoplast-induced (up-regulated) genes in the MapMan functional categories. Stars indicate significant enrichment using hyper-geometric testing (**P*≤ 0.05; ***P*≤ 0.01; ****P*≤ 0.001).

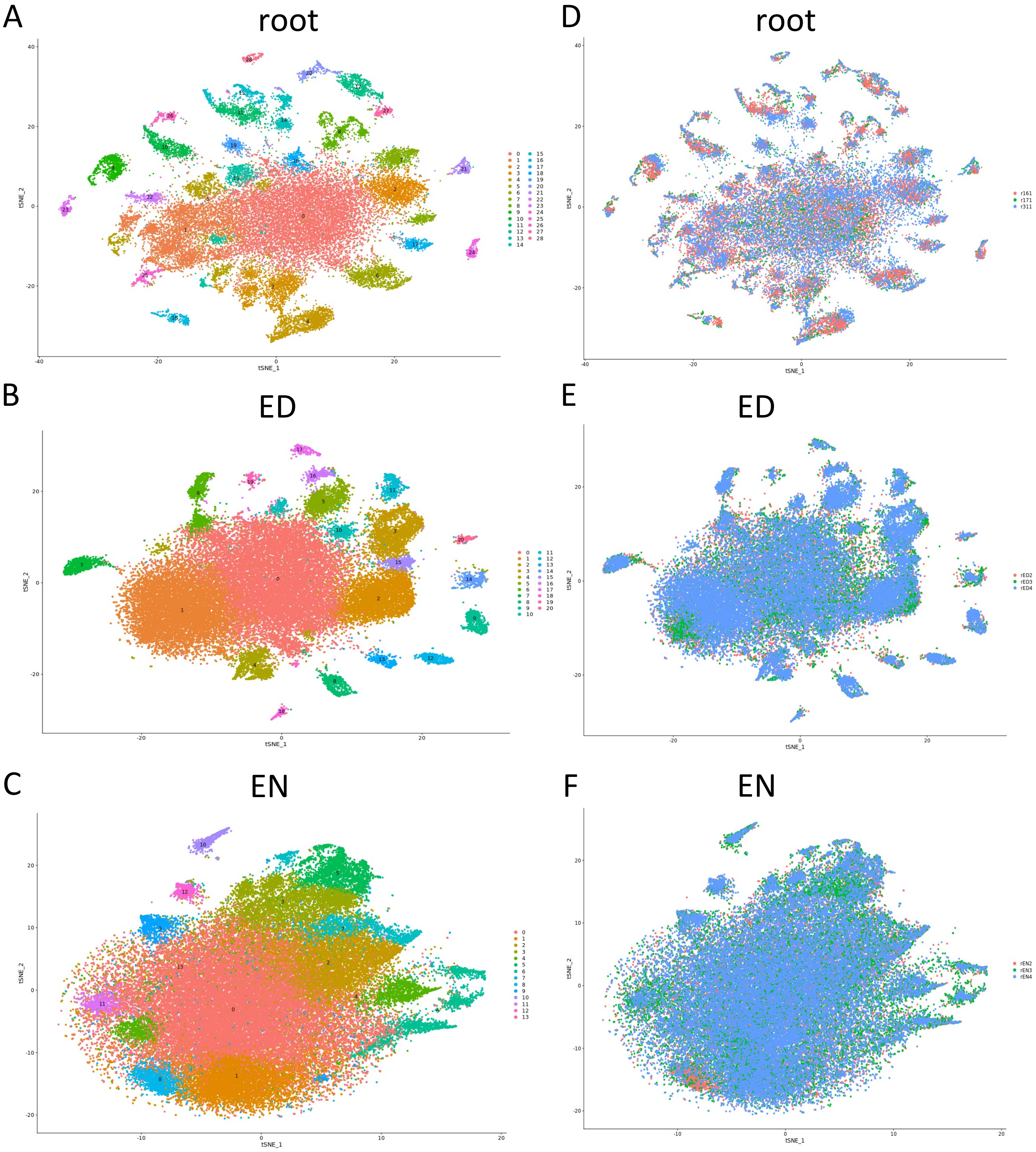

Supplemental Figure S5. Single-cell transcriptome of root and above-ground samples prior filtering. A-C, *t*-SNE projection plots of clusters identified from cells harvested from Col-0 root (A), above-ground at the end of the day (ED) (B), above-ground at the end of the night (EN) (C) tissue. Each dot represents the transcriptome from one cell. Cells represented by the same colors correspond to the same cluster. **D-F**, Same *t*-SNE projection plot as in A-C, but the cells color corresponds to the replicates.

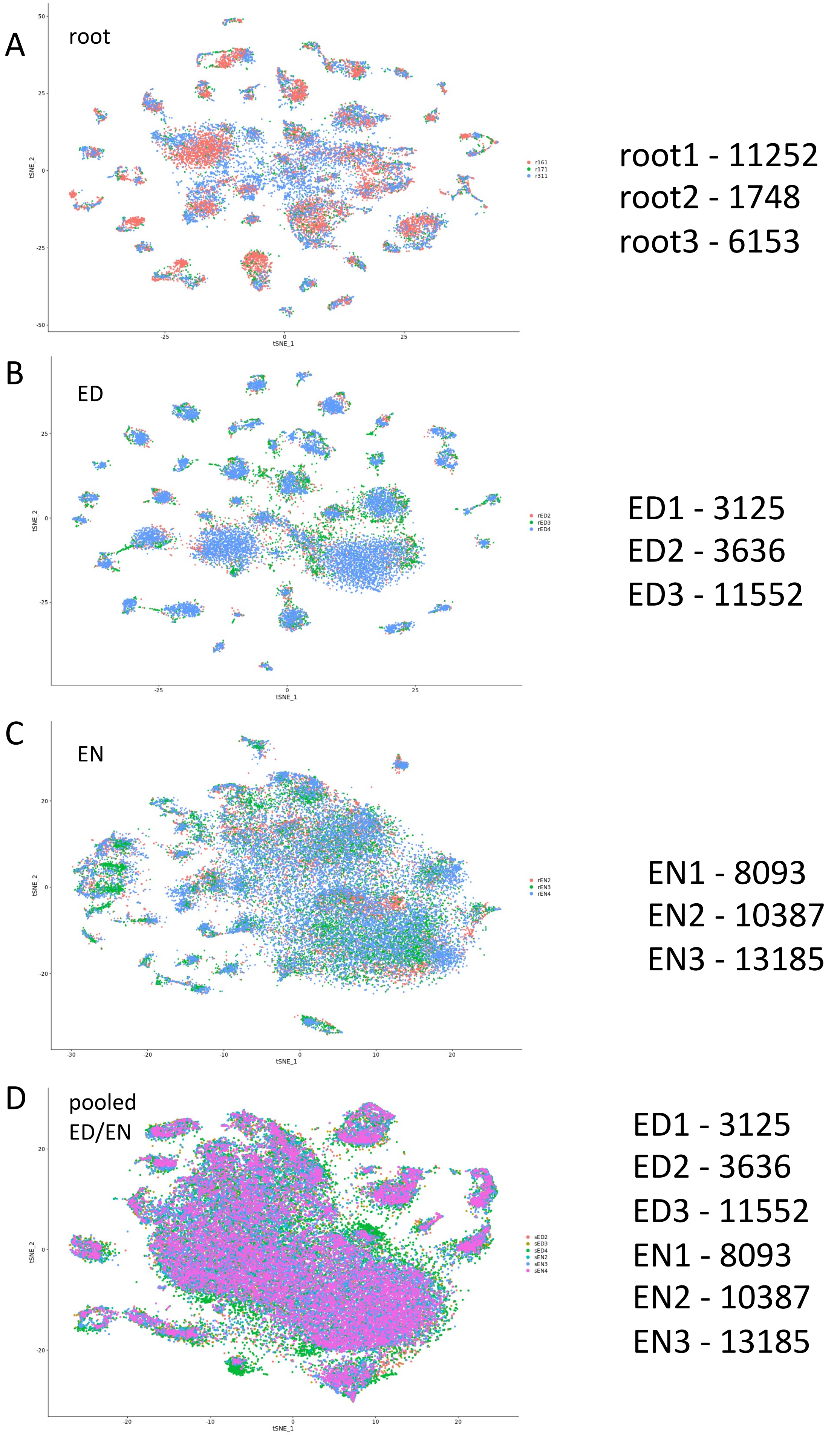

Supplemental Figure S6. *t*-SNE projection with cell colors for replicates. A-D, Same *t*-SNE projection plots as in Figure 2A-C (above-ground tissue) and 4A (root), but the color of the cells corresponds to the replicates. Number of cells for each replicate is given on the right. Abbreviations: end of the day (ED); end of the night (EN).

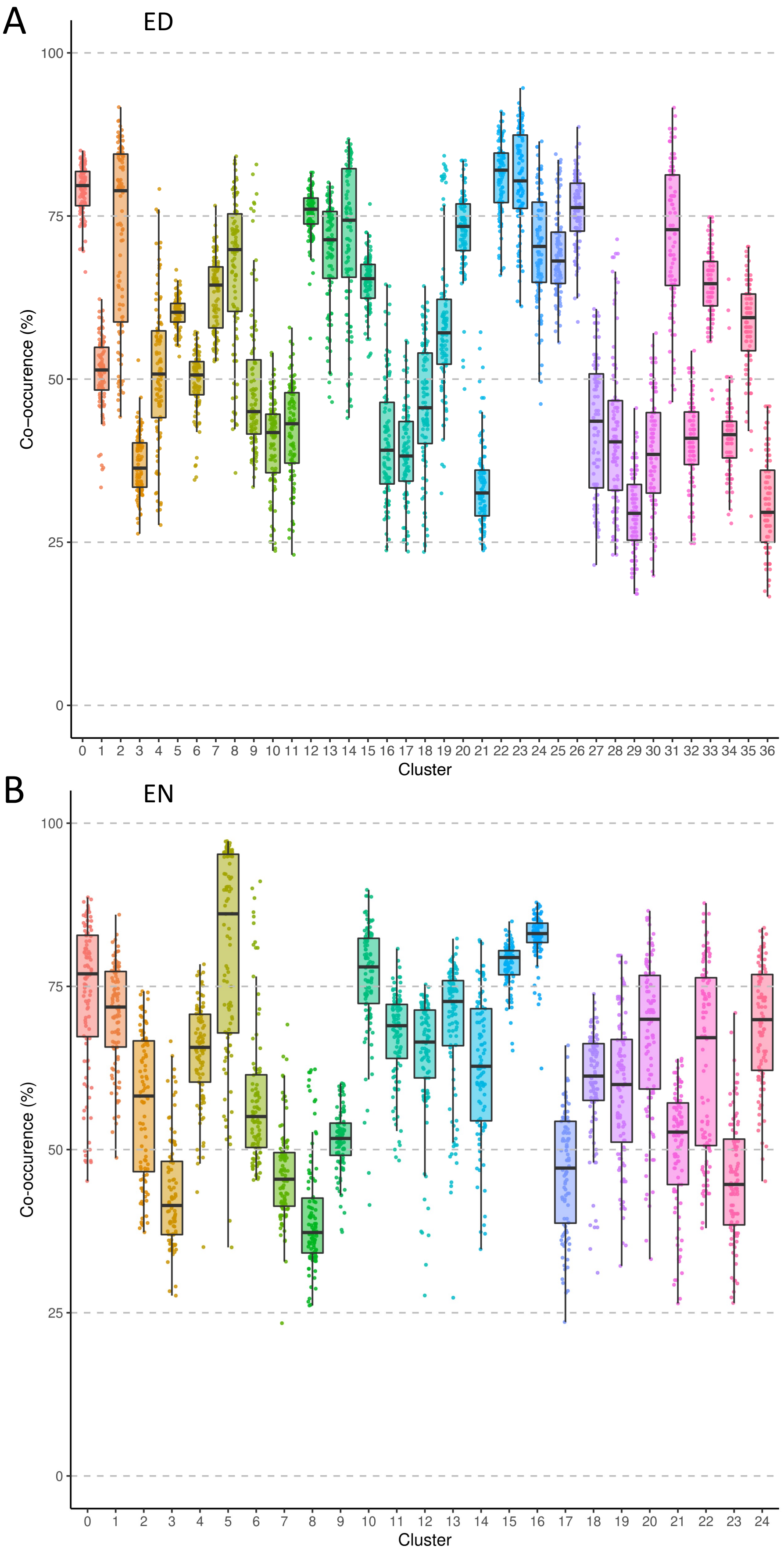

Supplemental Figure S7. Analysis of cluster robustness. Box plots representing the cluster robustness from 100 random subsets of 80% of the library reads. Percentage describes what proportion cells that previously formed a cluster are still co-occurring in a single cluster when using 80% of the library reads for the scRNAseq pipeline and clustering (while retaining the variable genes from the original clustering). The average co-occurrence was used to score the robustness for each cluster in the main Figures: 75-100% (***), 50-75% (**), 25-50% (*). **A**, End of day (ED); **B**, End of night (EN); **C**, pooled ED/EN; **D**, ED/EN without batch normalisation; **E**, root.

Box plots: center line, median; box limits, upper and lower quartiles; whiskers, 1.5x interquartile range.

C

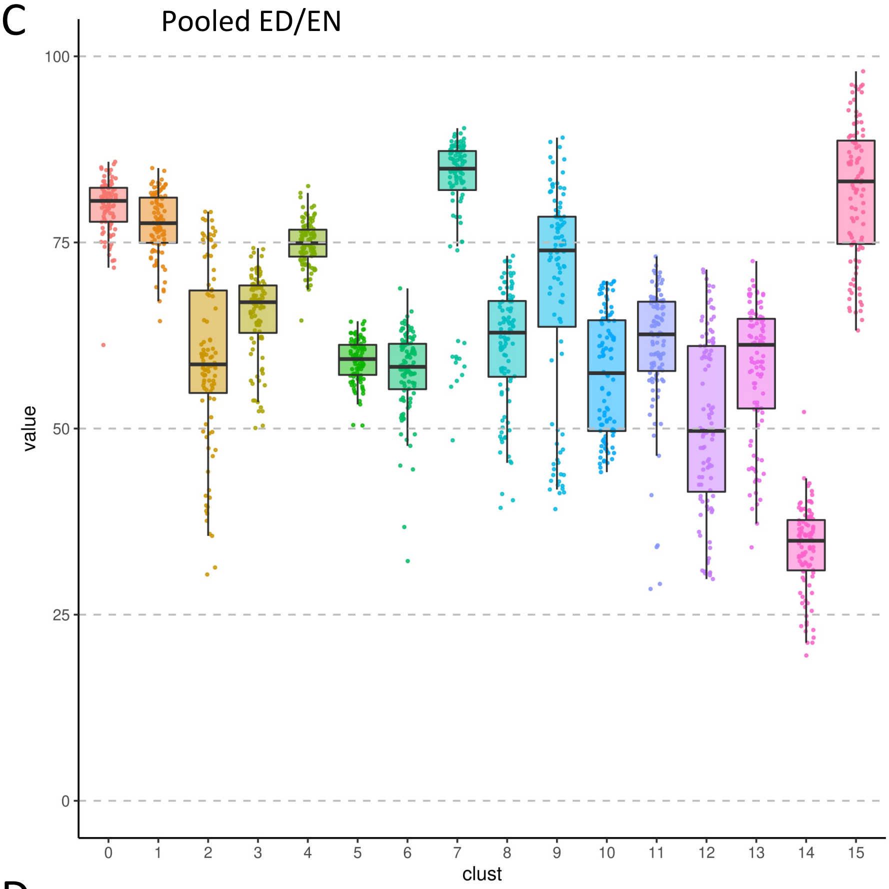

D

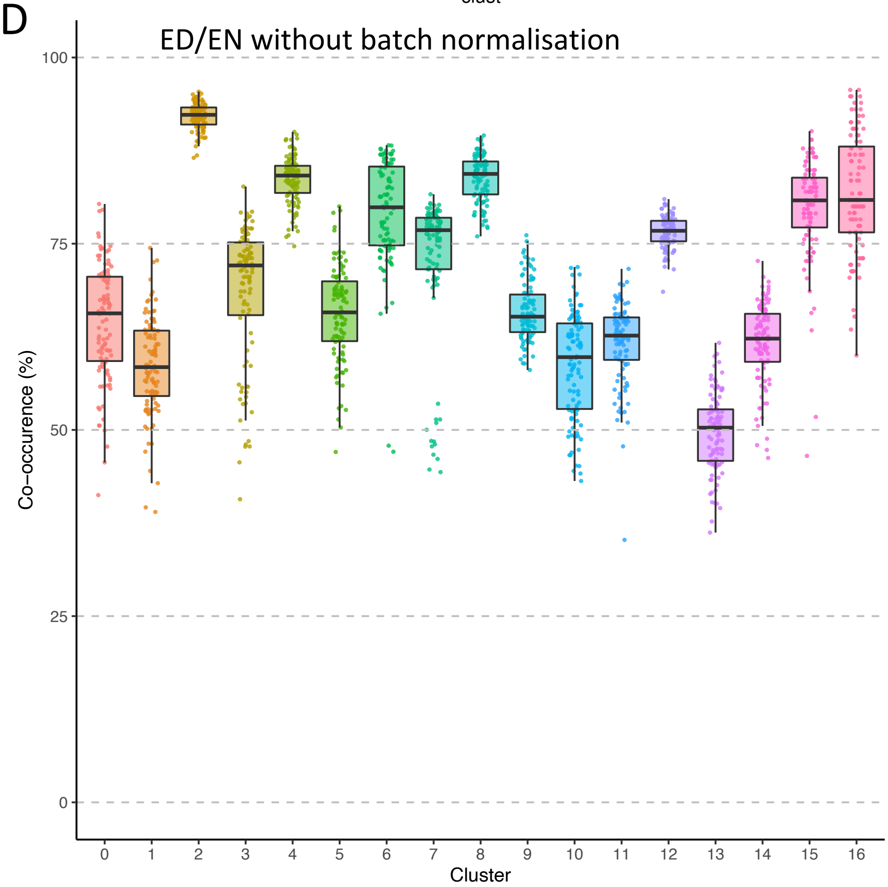

Supplemental Figure S7. Analysis of cluster robustness (continued). Box plots representing the cluster robustness from 100 random subsets of 80% of the library reads. Percentage describes what proportion cells that previously formed a cluster are still co-occurring in a single cluster when using 80% of the library reads for the scRNAseq pipeline and clustering (while retaining the variable genes from the original clustering). The average co-occurrence was used to score the robustness for each cluster in the main Figures: 75-100% (***), 50-75% (**), 25-50% (*). **A**, End of day (ED); **B**, End of night (EN); **C**, pooled ED/EN; **D**, ED/EN without batch normalisation; **E**, root.

Box plots: center line, median; box limits, upper and lower quartiles; whiskers, 1.5x interquartile range.

E

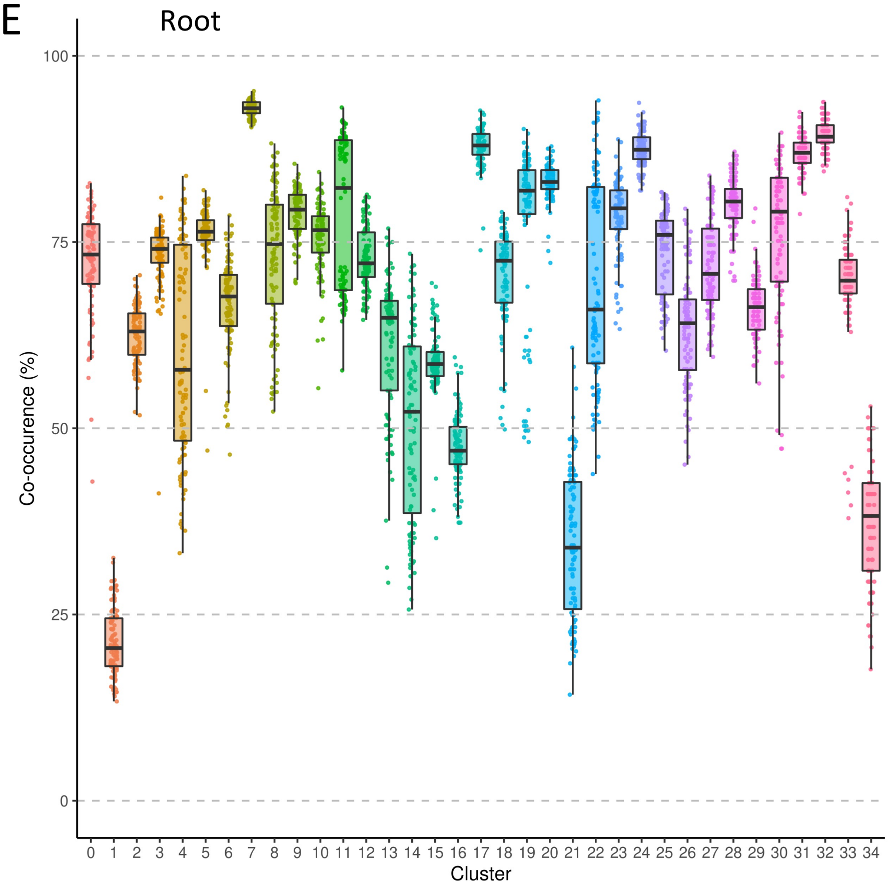

Supplemental Figure S7. Analysis of cluster robustness (continued). Box plots representing the cluster robustness from 100 random subsets of 80% of the library reads. Percentage describes what proportion cells that previously formed a cluster are still co-occurring in a single cluster when using 80% of the library reads for the scRNAseq pipeline and clustering (while retaining the variable genes from the original clustering). The average co-occurrence was used to score the robustness for each cluster in the main Figures: 75-100% (***), 50-75% (**), 25-50% (*). **A**, End of day (ED); **B**, End of night (EN); **C**, pooled ED/EN; **D**, ED/EN without batch normalisation; **E**, root.

Box plots: center line, median; box limits, upper and lower quartiles; whiskers, 1.5x interquartile range.

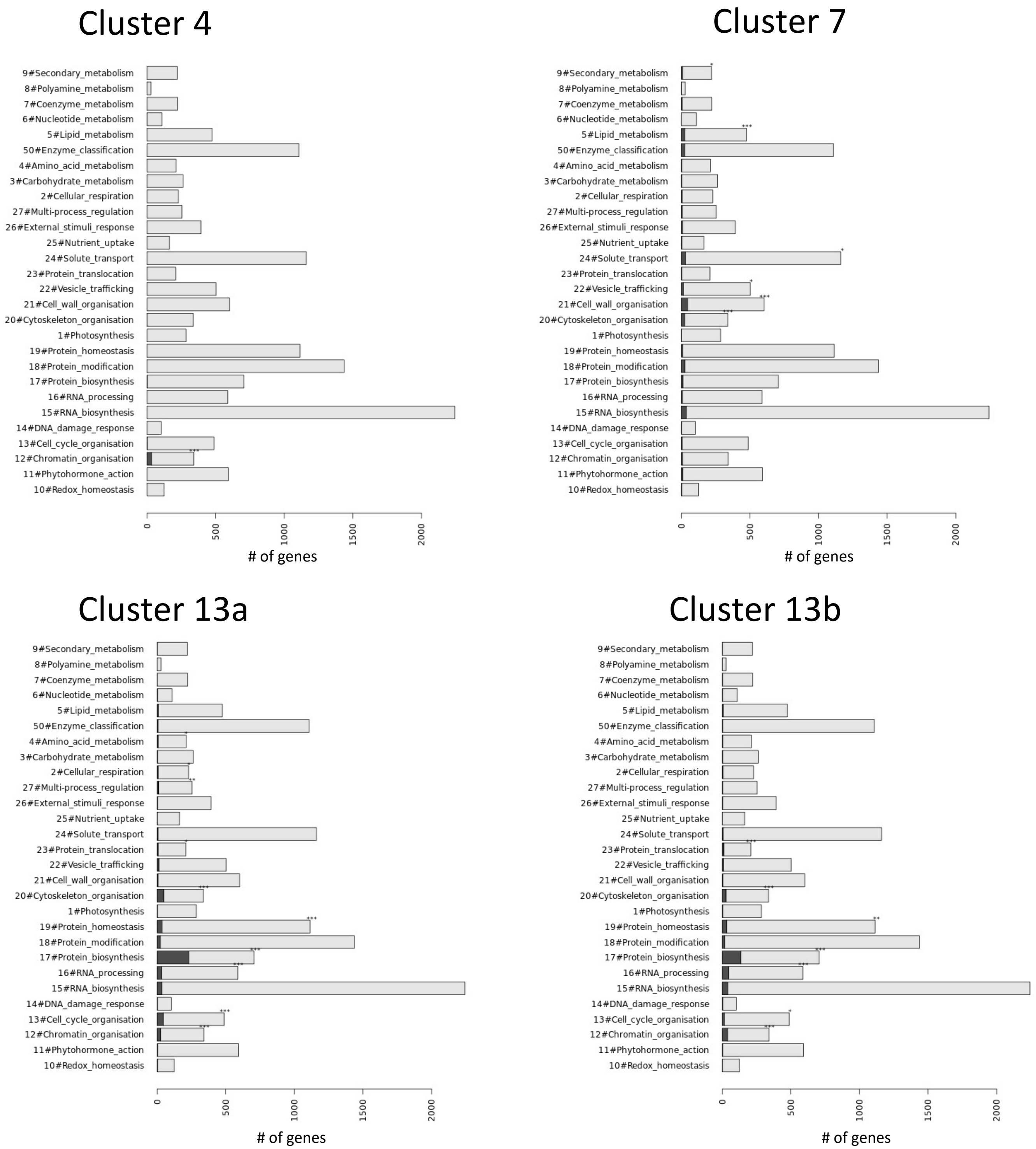

Supplemental Figure S8. Enrichment of cluster marker genes in the MapMan functional categories. Asterisks indicate significant enrichment using hypergeometric testing (**P*≤ 0.05; ***P*≤ 0.01; ****P*≤ 0.001).

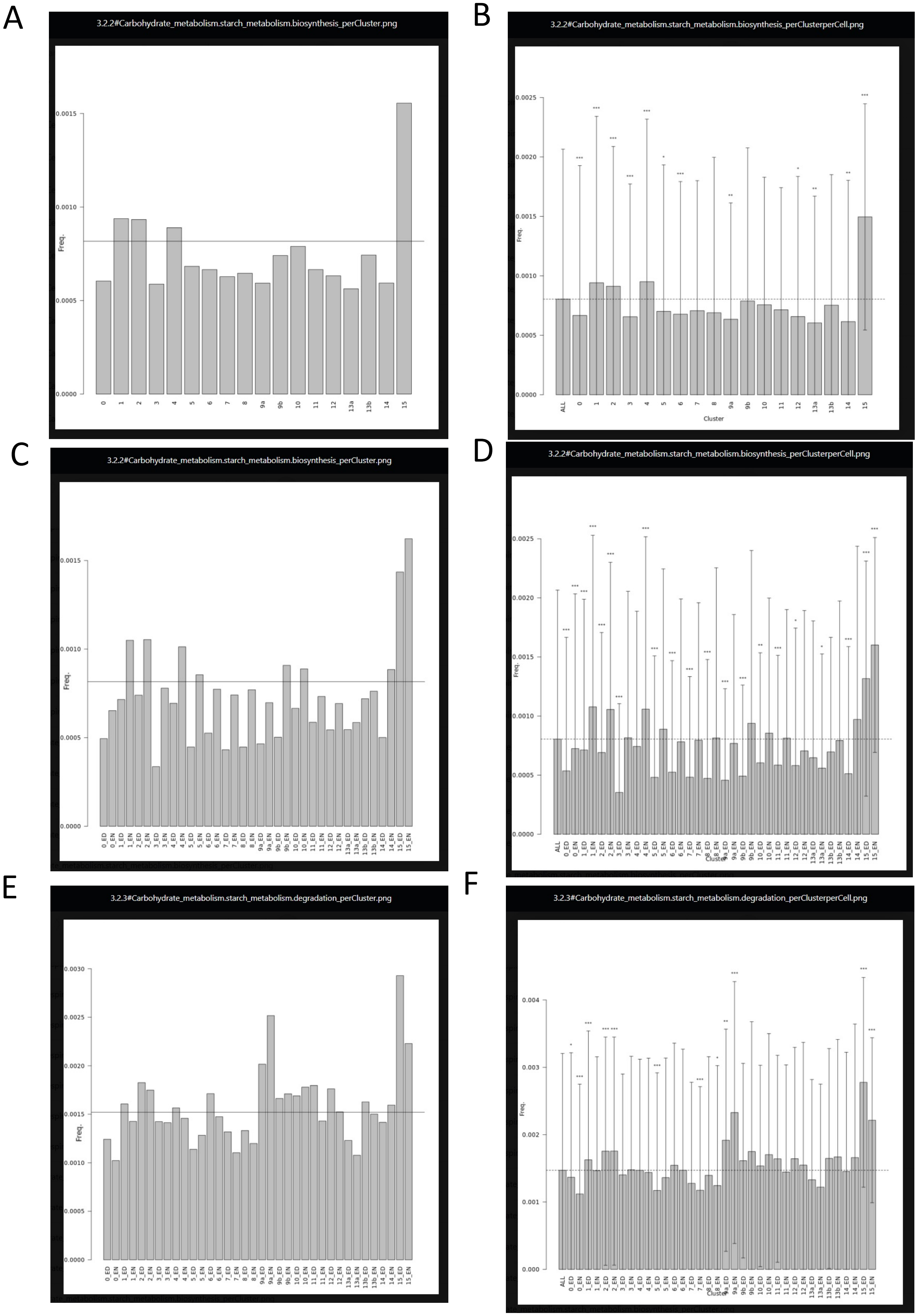

Supplemental Figure S9. Above-ground cluster (pooled ED/EN) enrichment for MapMan categories related to starch metabolism. A-B, Enrichment of MapMan category "starch biosynthesis" in clusters of pooled ED/EN cells. **C-D** Enrichment of MapMan category "starch biosynthesis" in clusters of pooled ED/EN cells split for ED and EN cells. **E-F**, Enrichment of MapMan category "starch degradation" in clusters of pooled ED/EN cells split for ED and EN cells. **A,C,E** shows the relative frequency of reads per cluster assigned to the analyzed MapMan category. **B,C,F** shows the relative frequency of reads per cell per cluster assigned to the analyzed MapMan category. Error bars indicate s.d. Stars indicate significant difference to all cells using Student's *t*-test (**P*≤ 0.05; ***P*≤ 0.01; ****P*≤ 0.001).

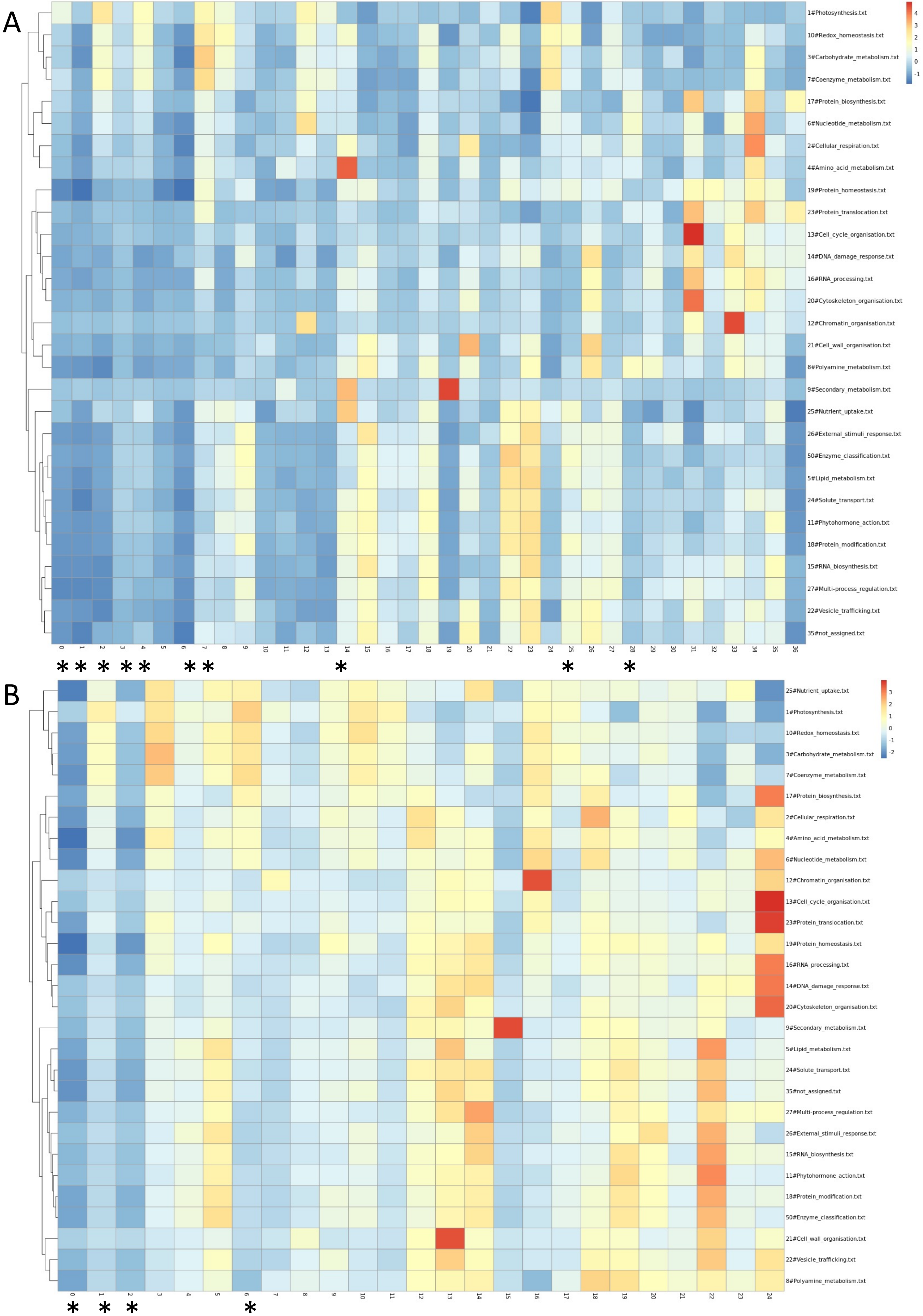

Supplemental Figure S10 Enrichment of MapMan level 1 categories in above-ground samples. Enrichment of MapMan categories in rosette samples (**A**, End of day (ED); **B**, End of night (EN); **C**, pooled ED/EN) plotted as a heatmap. The mesophyll clusters are indicated with asterisks. The color scale denotes the z-score of the transcript abundance for each MapMan category in each cluster.

C

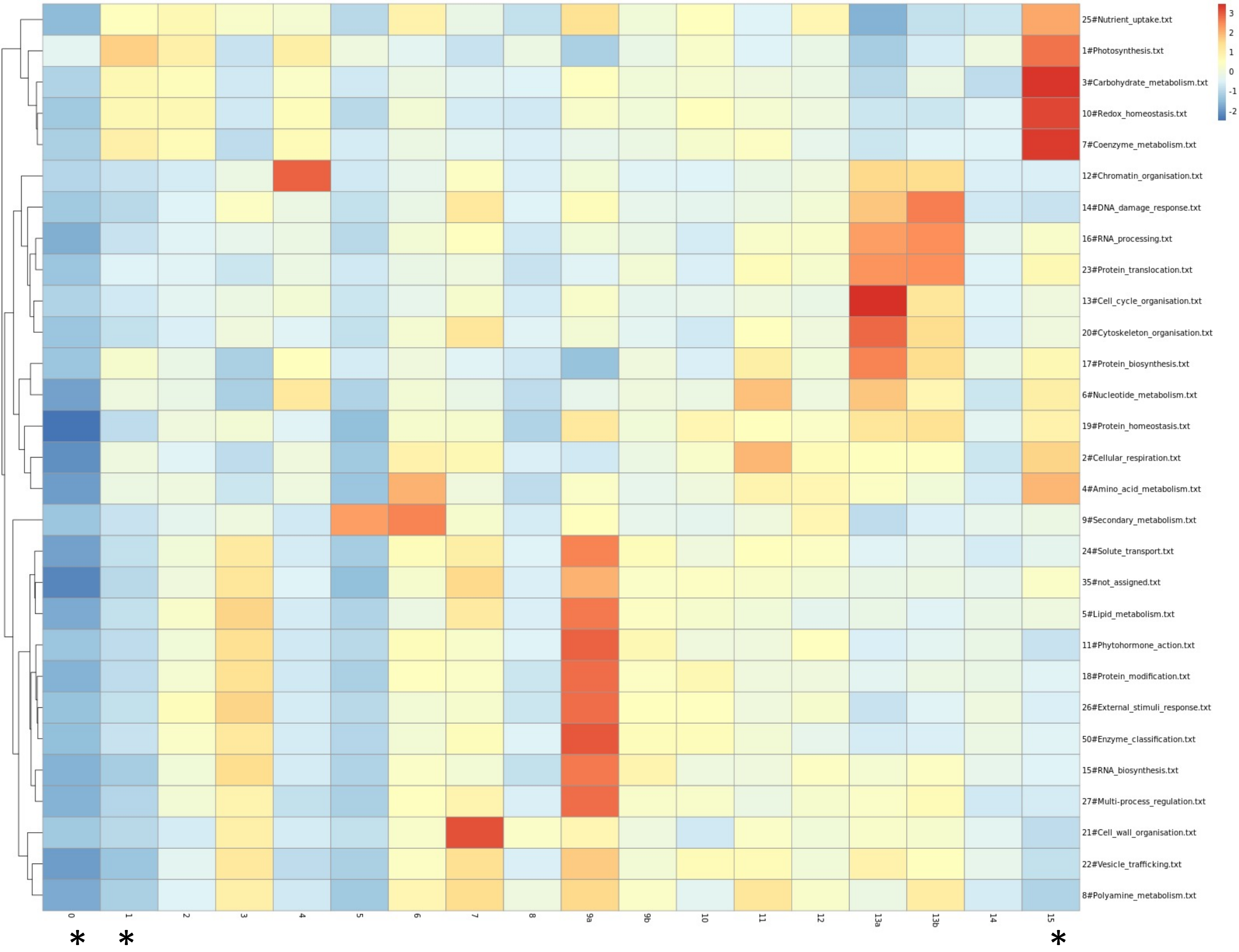

Supplemental Figure S10 (continued). Enrichment of MapMan level 1 categories in above-ground samples. Enrichment of MapMan categories in rosette samples (**A**, End of day (ED); **B**, End of night (EN); **C**, pooled ED/EN) plotted as a heatmap. The mesophyll clusters are indicated with asterisks. The color scale denotes the *z*-score of the transcript abundance for each MapMan category in each cluster.

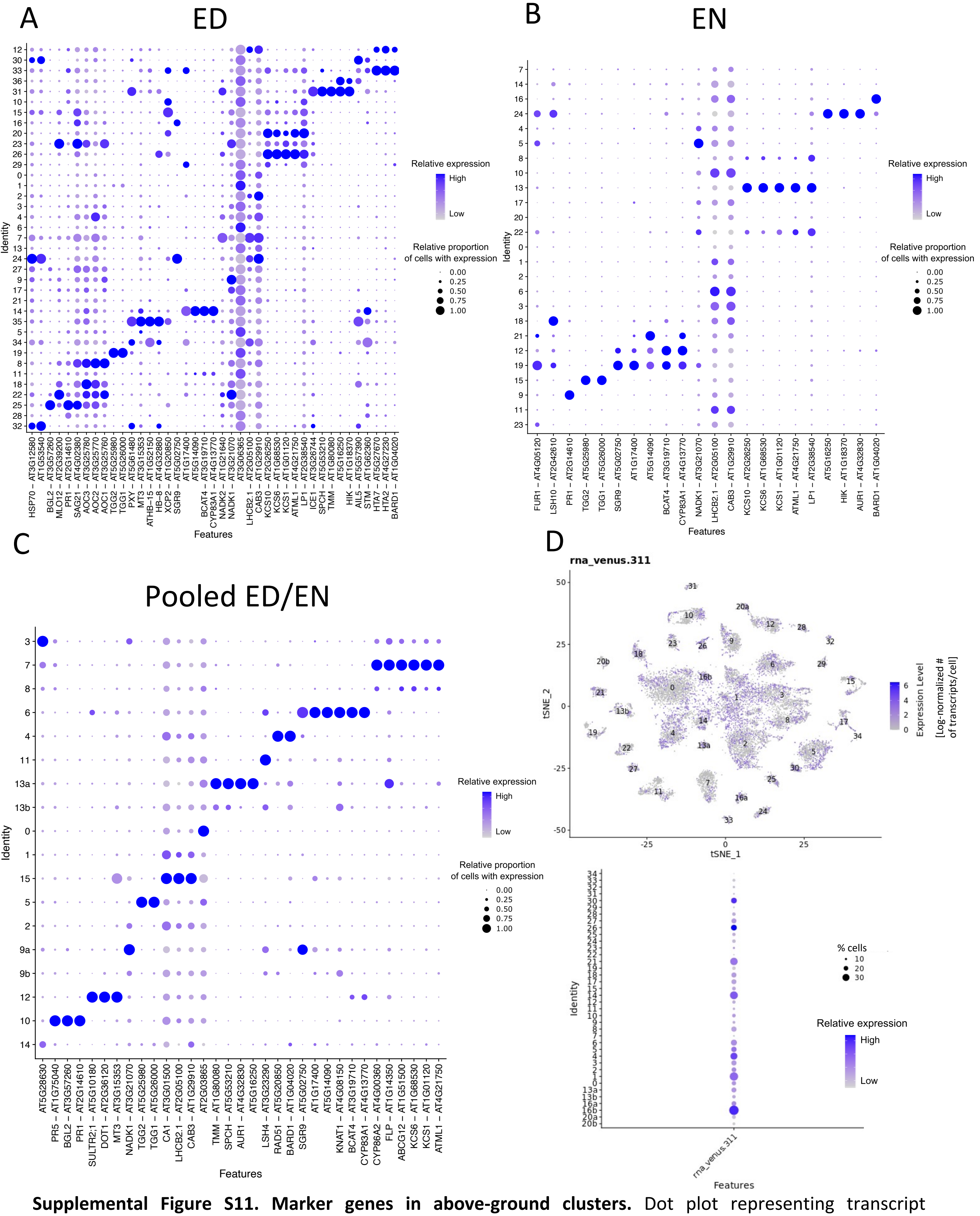

Supplemental Figure S11. Marker genes in above-ground clusters. Dot plot representing transcript accumulation of known and novel marker genes for each cluster in **A**, above-ground tissue at the end of the day (ED), **B**, above-ground tissue at the end of the night (EN) and, **C**, pooled ED/EN. The tissue specificity of the marker genes is extensively discussed the results chapter and in Supplemental Texts S2-4. The color scale denotes the relative expression (average per cell). The dot size denotes the relative proportion of cells with expression. Color scale and dot size are scaled to the minimum/maximum expression level and proportion of cells with expression, respectively, of the transcript within the clusters; for the absolute percentages see Supplemental Table S20. **D**, Dot plot of *H2B-Venus* expression (marker line V311) marking differentiated cells in root clusters. Dot size is scaled to the amount of cells per cluster with *H2B-Venus* expression.

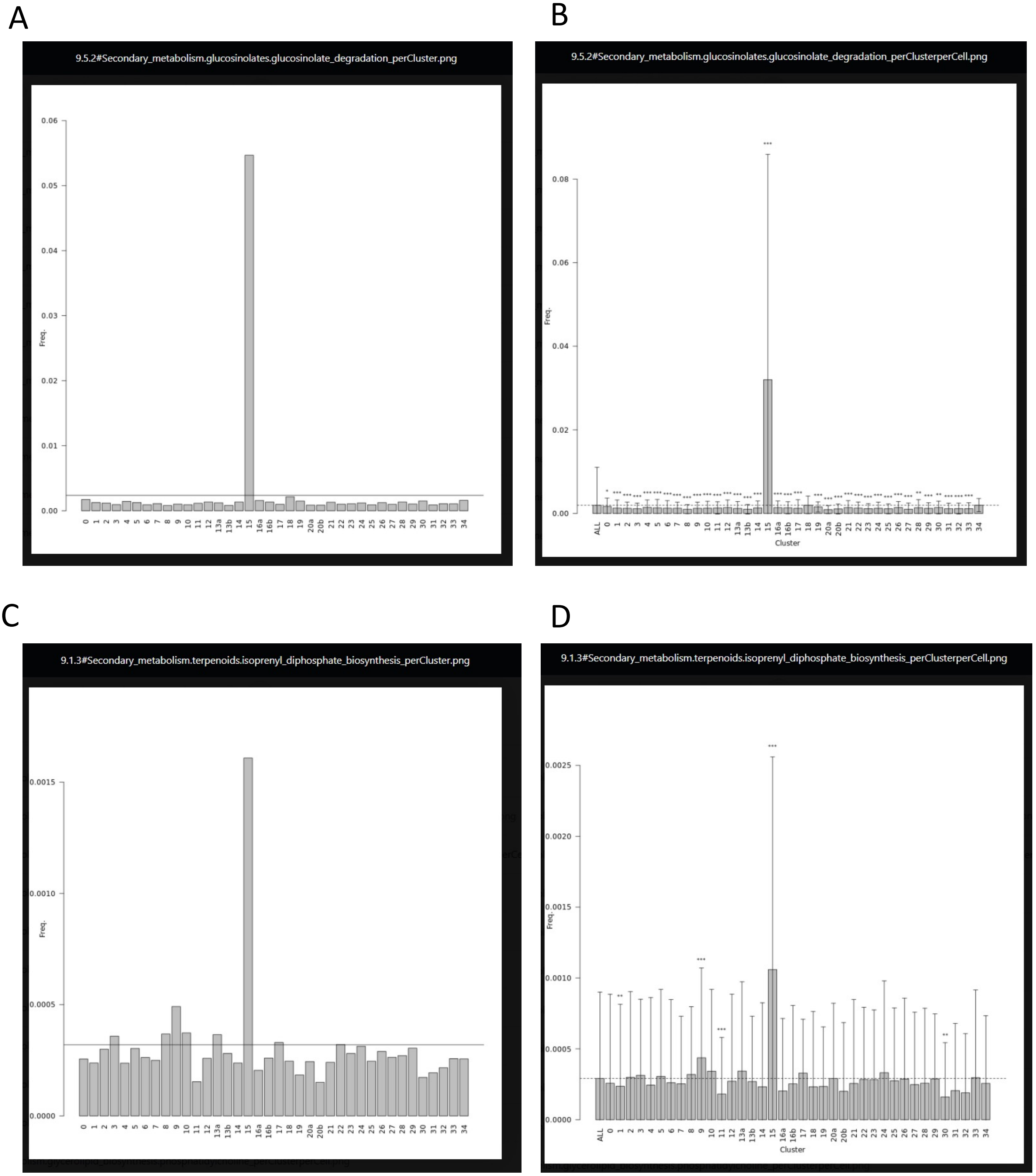

Supplemental Figure S12. Root cap enriched MapMan categories related to secondary metabolism. A-D, Root cap (cluster 15) shows significant enrichment of MapMan categories related to secondary metabolism. **A,C** shows the relative frequency of reads per cluster assigned to the analyzed MapMan category. **B,C** shows the relative frequency of reads per cell per cluster assigned to the analyzed MapMan category. Error bars indicate s.d. Stars indicate significant difference to all cells using Student's *t*-test (*$P\leq 0.05$; **$P\leq 0.01$; ***$P\leq 0.001$).

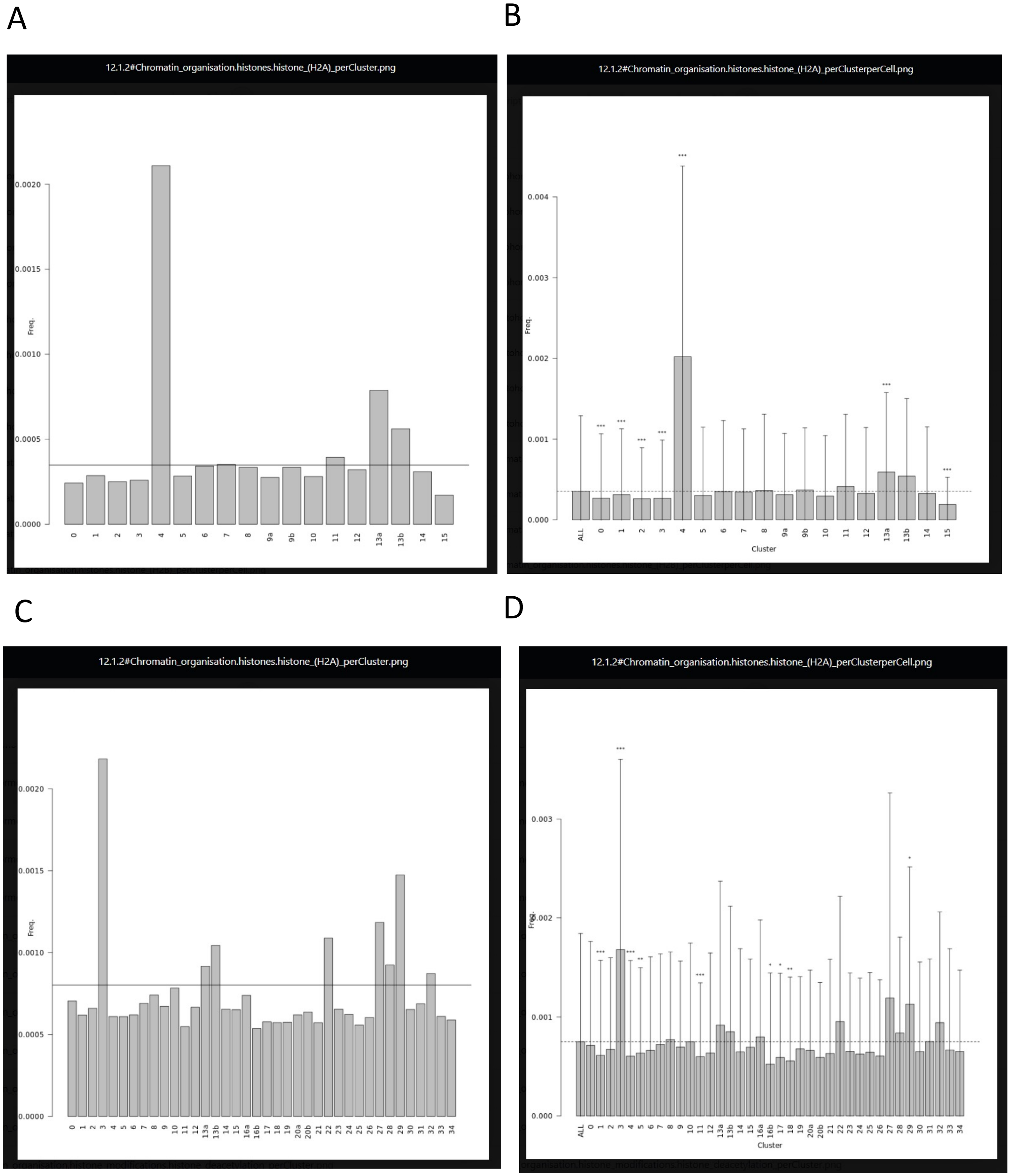

Supplemental Figure S13. MapMan category "chromatin organization" enriched in root and above-ground clusters related to RAM and SAM, respectively. A-B, Enrichment of MapMan category "chromatin organization" in clusters of pooled ED/EN cells. **C-D,** Enrichment of MapMan category "chromatin organization" in clusters of root cells.

A,C shows the relative frequency of reads per cluster assigned to the analysed MapMan category. B,C shows the relative frequency of reads per cell per cluster assigned to the analysed MapMan category. Error bars indicate s.d. Stars indicate significant difference to all cells using Student's t-test (*P≤ 0.05; **P≤ 0.01; ***P≤ 0.001).

A

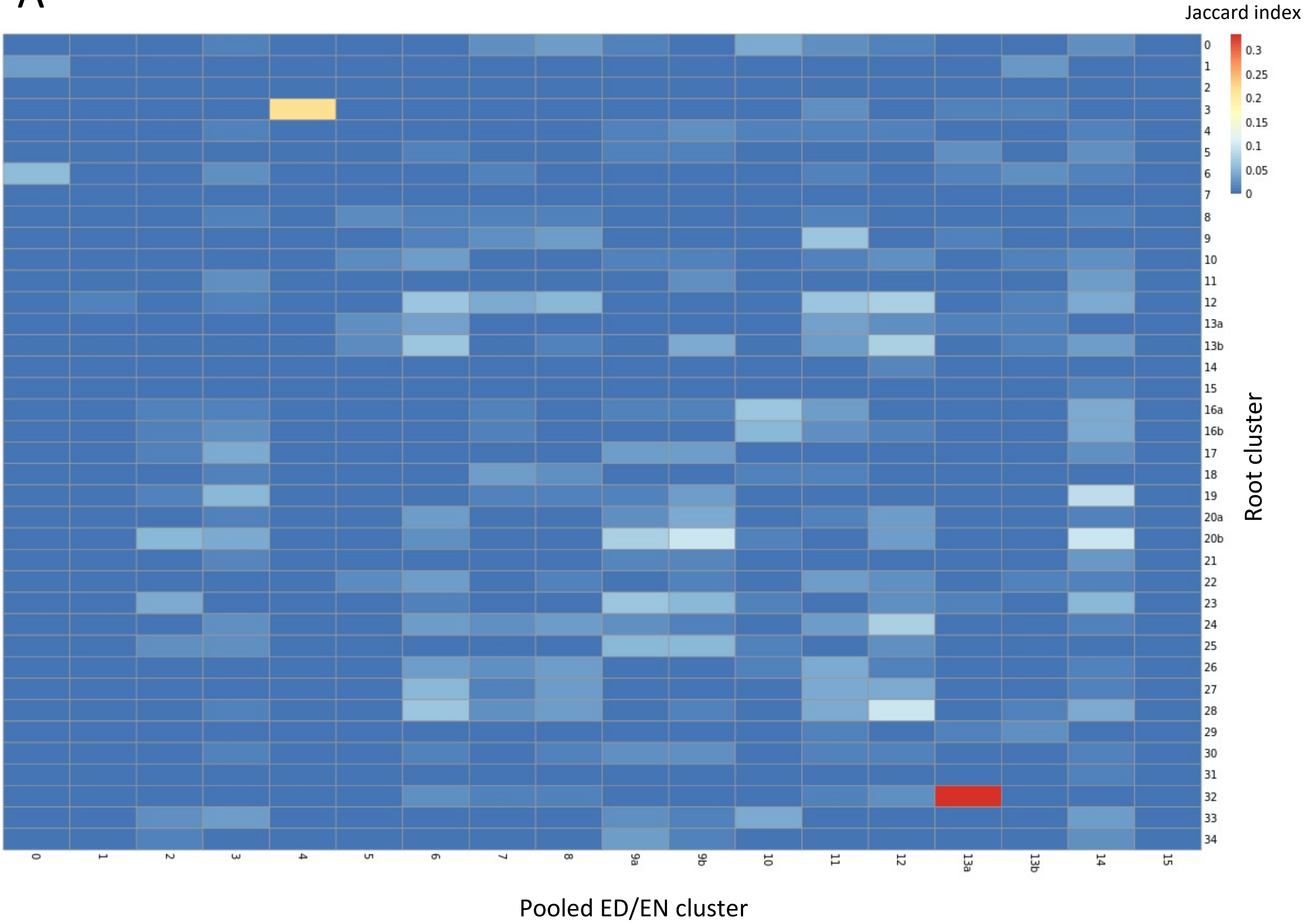

B AT1G02730, AT1G03780, AT1G08560, AT1G14350, AT1G18370, AT1G20930, AT1G30690, AT1G50490, AT1G76310, AT1G76540, AT2G26760, AT2G27970, AT2G28620, AT2G29550, AT2G33560, AT3G14190, AT3G19050, AT3G19590, AT3G23890, AT3G25980, AT3G44050, AT3G51280, AT3G51670, AT4G05190, AT4G32830, AT4G33270, AT4G37450, AT5G06150, AT5G13840, AT5G15510, AT5G51600, AT5G60930

Supplemental Figure S14. Overlap of markers between root and above-ground (pooled ED/EN) clusters. **A**, Heatmap of top 50 (based on logFC) cluster marker overlaps between root and pooled ED/EN clusters. Color scale indicates Jaccard index (ratio of union over intersection). **B**, 32 out of 82 genes of all overlapping marker genes between cluster 32 (root) and cluster 13a (pooled ED/EN) are annotated as cell cycle (GO:0007049). Abbreviations: end of the day (ED); end of the night (EN).

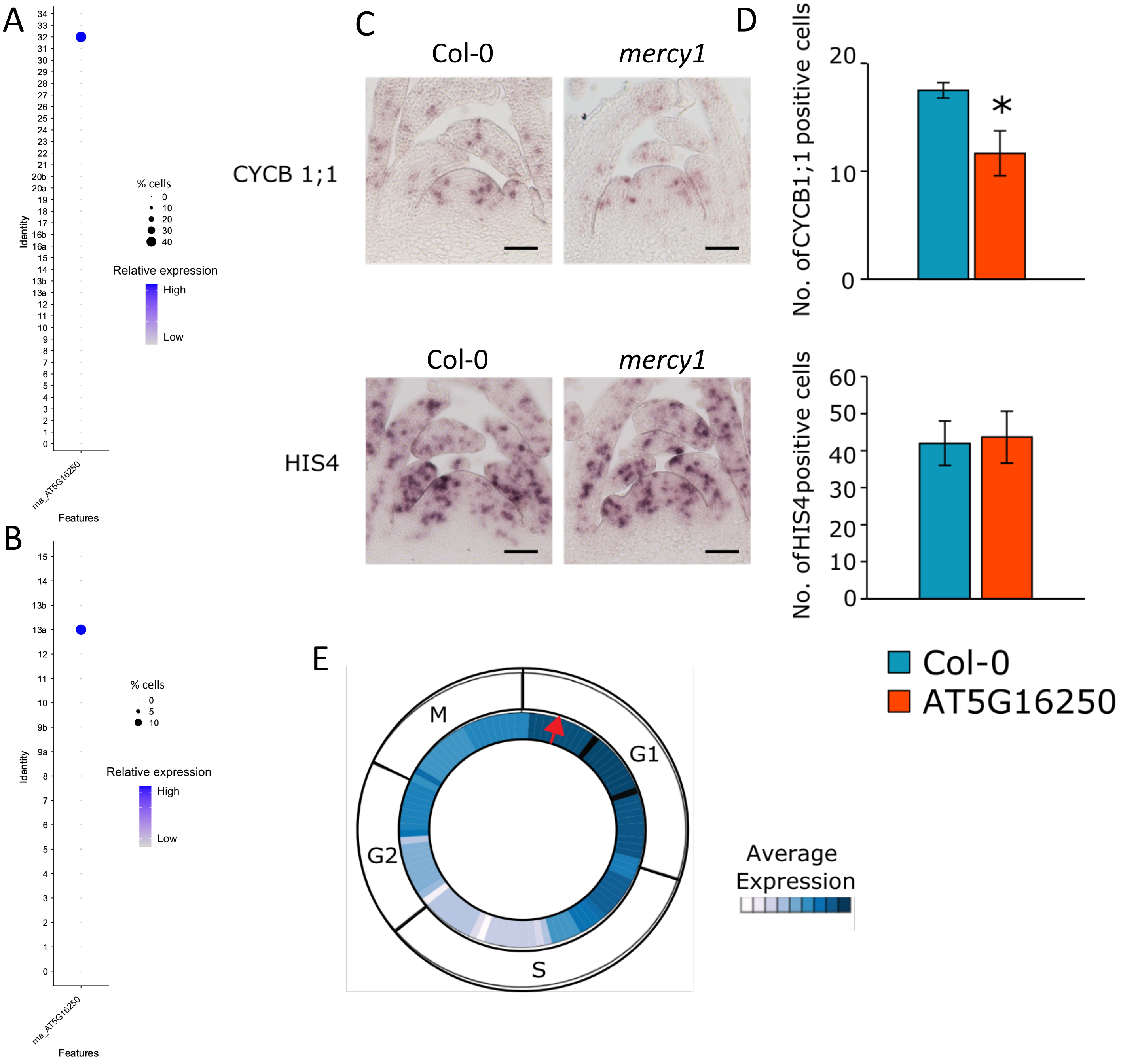

Supplemental Figure S15. Characterization of molecular function of *AT5G16250* (*MERCY1*). A, Dotplot of expression of all *MERCY1* transcripts in root (see Figure 4) and **B**, above-ground (pooled ED/EN) clusters (see Figure 2). Dot size is scaled to the proportion of cells per cluster with *MERCY1* expression. **C**, RNA *in situ* hybridization using *CYCLINB1;1 (CYCB1;1)* and *HISTONE4 (H4)* as probes on longitudinal section through meristems of Col-0 wild-type and *mercy1* mutant plants harvested at the end of the day. Scale bars equal 100 µm. **D**, Number of *CYCB1;1* and *H4* positive cells at the SAM of Col-0 and *mercy1* plants (n>3). Error bars indicate s.d. Statistical significance from control conditions was calculated using Student *t*-test (*$P \leq 0.05$). **E**, Expression level of *MERCY1* during different stages of cell cycle determined using CycleBase 3.0 software. Relative expression is shown as running average from low (white) to high (dark blue), with a red arrow indicating the estimated time of peak expression.

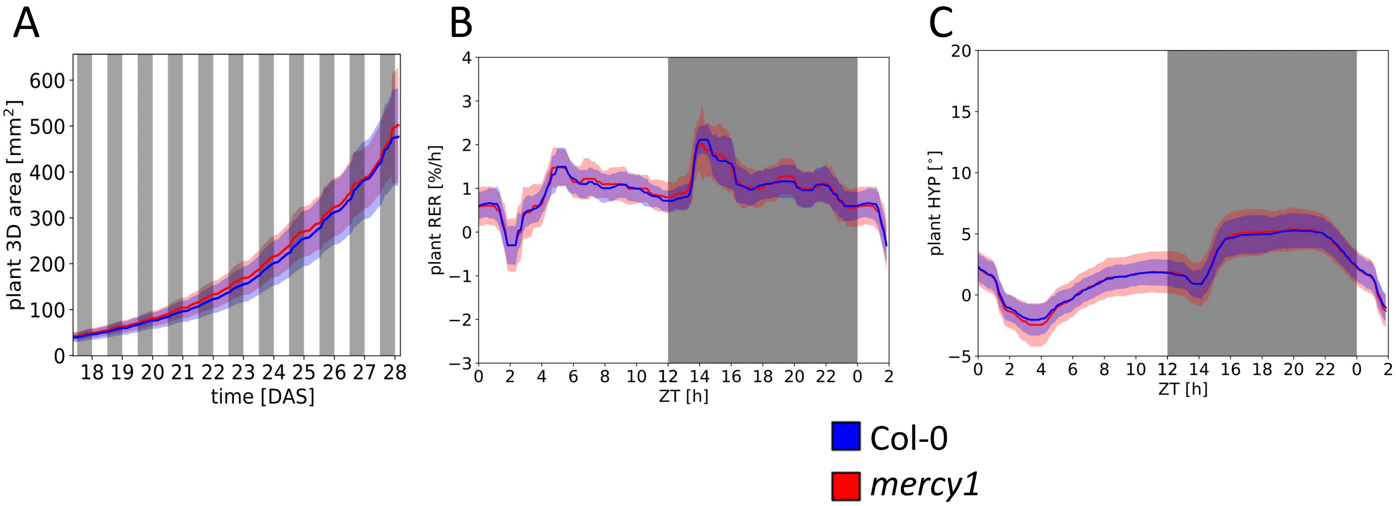

Supplemental Figure S16. Analyzing growth behavior of *mercy1*. A, Rosette area increased over time (17-28 days after sowing, DAS) determined by 3D imaging. **B**, Diurnal relative expansion growth rate (RER) averaged over all sequential 24-hour periods. **C**, Diurnal hyponastic angle (HYP) over all sequential 24-hour periods.

Lines and color-shaded areas represent mean and standard deviation, respectively, of > 20 plants, grown in a 12h photoperiod. We used an established 3D phenotyping system (Apelt *et al.,* 2015) for these analyzes.

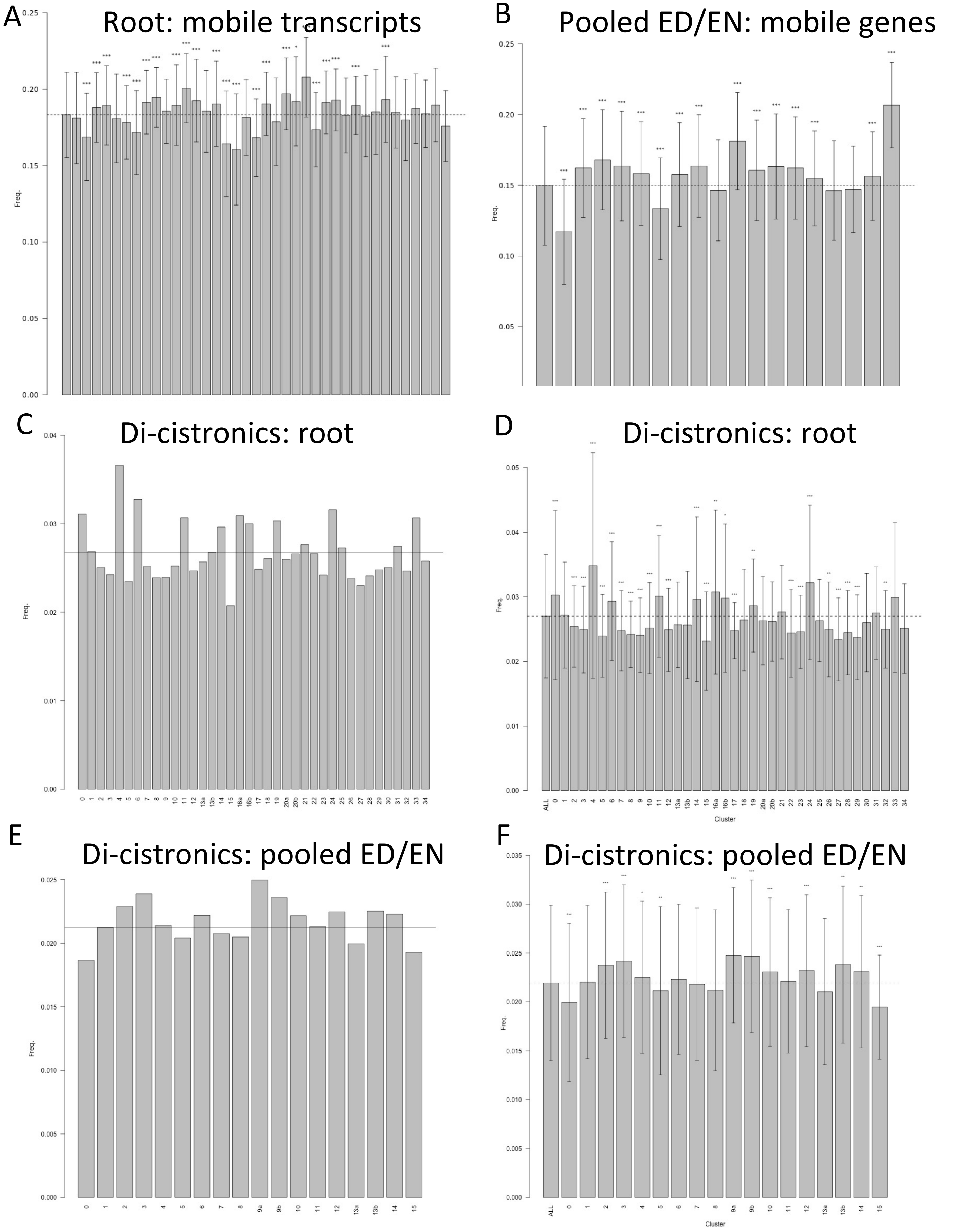

Supplemental Figure S17. Enrichment of graft-mobile annotated transcripts and di-cistronic transcripts in root and shoot (pooled ED/EN) clusters. A-B, Enrichment of mobile transcripts. **C-F**, Enrichment of di-cistronic transcripts.

C,E shows the relative frequency of reads per cluster assigned to the analysed MapMan category. A,B,D,F shows the relative frequency of reads per cell per cluster assigned to the analysed MapMan category. Error bars indicate s.d. Stars indicate significant difference to all cells using Student's t-test (*P≤ 0.05; **P≤ 0.01; ***P≤ 0.001).

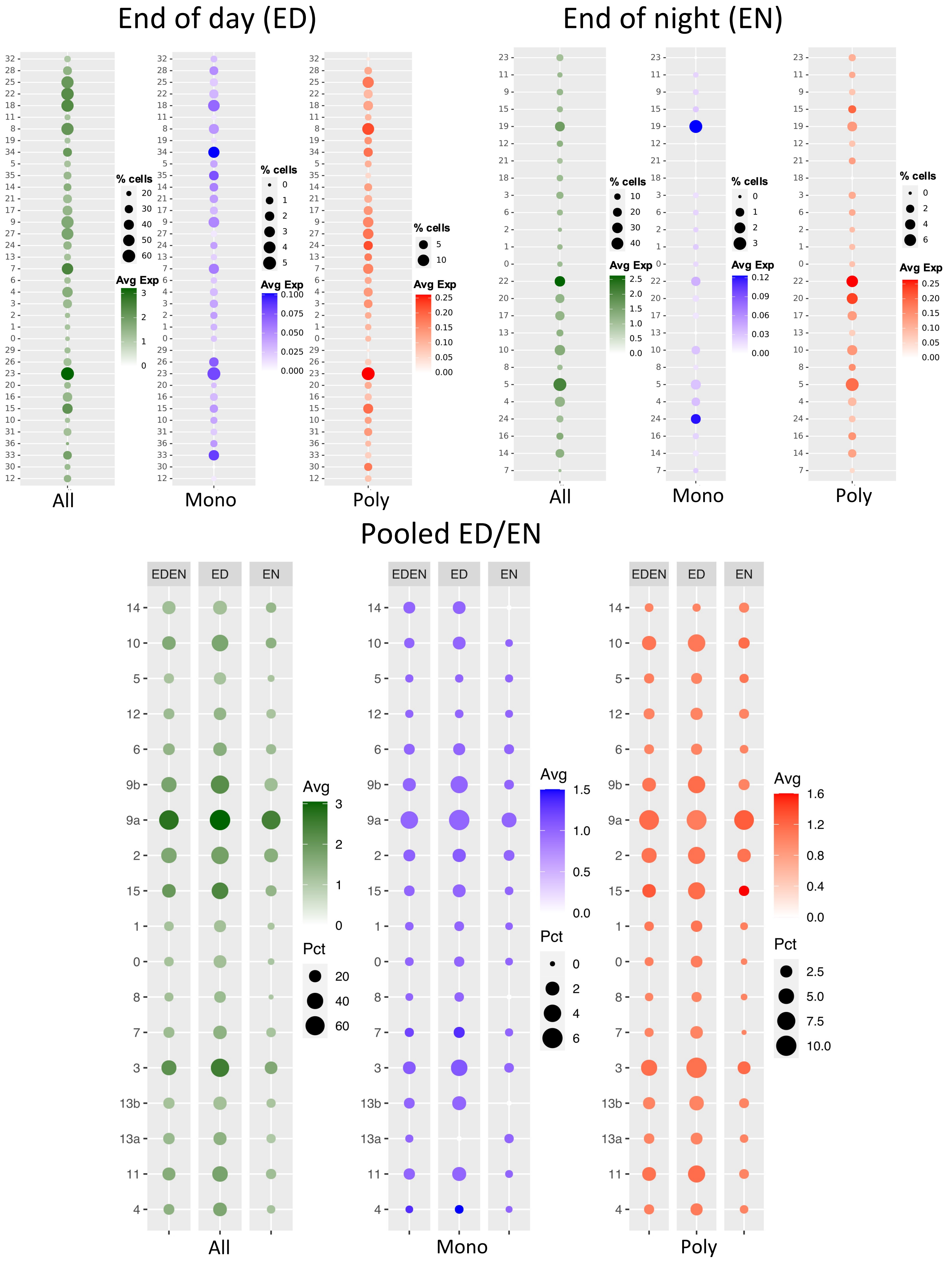

Supplemental Figure S18. Dot plots representing transcript accumulation of *CK1*, mono-cistronic *CK1* and *CK1-TLS* transcripts for each cluster in above-ground clusterings. Cells with *CK1* expression are considered for calculating the average expression.

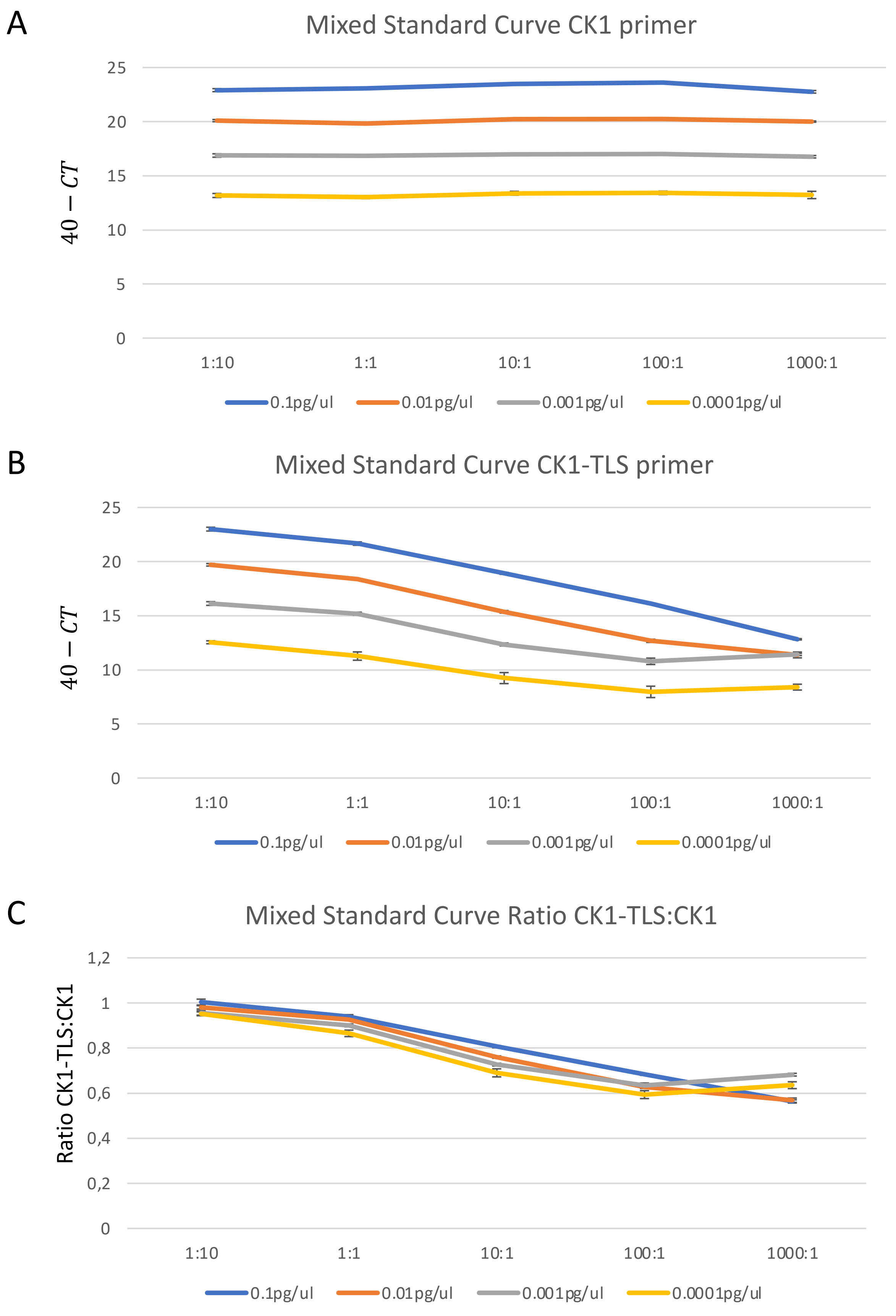

Supplemental Figure S19. RT-qPCR of standard curves created using purified PCR products of *CK1* and *CK1-TLS*. A, RT-qPCR of *CK1*/*CK1-TLS* mixtures using *CK1* FP and *CK1* RP primers. **B**, RT-qPCR of *CK1*/*CK1-TLS* mixtures using *CK1* FP and *CK1-TLS* RP primers. **C**, Ratios *CK1-TLS*:*CK1* of RT-qPCR data shown in A and B.
PCR products of *CK1* and *CK1-TLS* were mixed at different ratios and at different concentrations. Ratios shown on the x-axis labels are *CK1:CK1-TLS*.

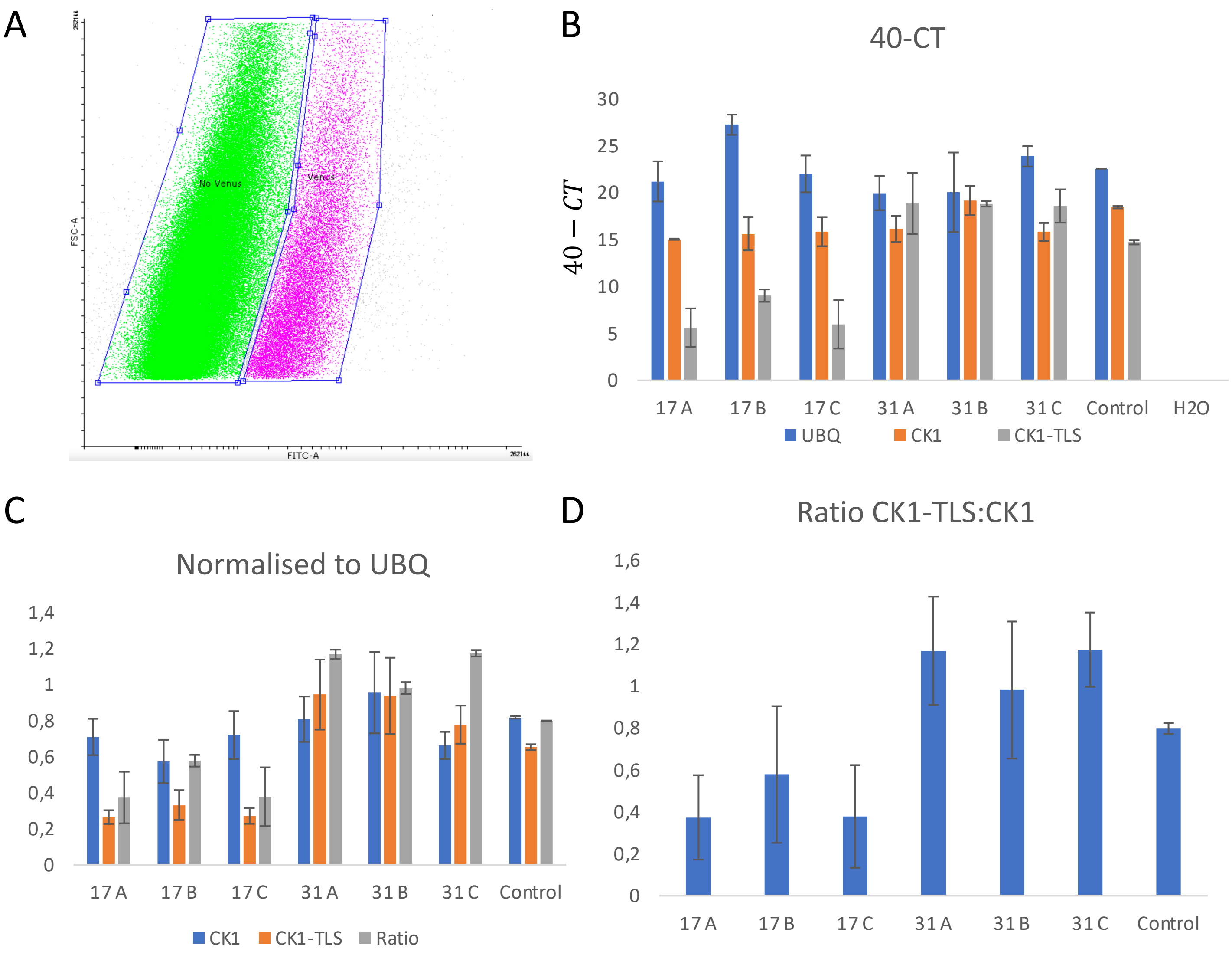

Supplemental Figure S20. FACS of tissue-specific protoplasts and RT-qPCR of isolated cDNA. A, FSC-A v FITC plot of protoplasts isolated from root tissues. 'No Venus' gate labels the population sorted as a control, and 'Venus' gate labels the population containing H2B-Venus and was sorted for tissue-specific RNA isolation. **B**, 40-Ct plot of RT-qPCR of cDNA libraries from FACS sorted protoplasts. 17A,B,C refers to H2B-Venus containing cells marking the epidermis (Cluster17), each of A,B and C is a separate biological repeat. 31A,B,C refers to H2B-Venus containing cells marking the endodermis (Cluster31), each of A,B and C is a separate biological repeat. Control refers to mixed sample of 'No Venus' protoplasts from all samples. *UBQ* is *UBQ10*, *CK1* is *CK1* amplified using *CK1* FP and *CK1* RP primers, *CK1-TLS* is *CK1-TLS* amplified using *CK1* FP and *CK1-TLS* RP primers. Each column is the geometric mean of 3 RT-qPCR repeats. **C**, RT-qPCR data shown in B normalized to values for *UBQ*. **D**, Ratio of *CK1-TLS*:*CK1* from RT-qPCR data shown in C.

Supplemental Methods S1. Protoplast isolation.

Root protoplasts: For the root cultures seeds were surface sterilized by washing in a solution of 10 mg/ml dichloroisocyanurate in 90 % v/v ethanol for 10 min with rotation and then rinsed three times with absolute ethanol. Seeds were dried and subsequently resuspended in 0.15 % w/v microagar (Duchefa). After stratification (4 °C for 24 h in the dark), the seeds were placed in four rows on 0.5 × Murashige and Skoog (MS), 0.1 % w/v microagar plates supplemented with 0.6 % w/v sucrose and 20 mM β-Estradiol (Diluted in ethanol, Sigma). The *β*-Estradiol solution was filter-sterilized and added to the autoclaved MS media before it was poured in the plates. Plates were then transferred vertically to a growth chamber with 12 h light/dark photoperiod at 22 °C.

Seven days after germination (DAG) the root tissue of seedlings was harvested 75 min before the ED time point (15 min is the duration of harvesting and dicing of the tissue, 60 min the duration of protoplasting). For each sample, roots of seedlings grown on five plates (with four rows of vertically grown seedlings) were cut with a single razor stroke (BAYHA Nr.22) approximately 1 cm below the hypocotyl (Supplemental Fig. S1A) and transferred to a petri dish (35 x 10 mm) containing 10 ml Solution A (600 mM Mannitol, 2 mM $MgCl_2$, 0.1 % w/v BSA, 2 mM $CaCl_2$, 2 mM MES pH 5.7, 10 mM KCl), 2 % w/v cellulase (Duchefa RS) and 0.2 % w/v pectinase (Sigma #17389) and cut in small pieces by vertical strokes with a straight stainless steel double edge razor blade and incubated at room temperature (23 °C) for 1 h on a horizontal shaker set at 80 rpm. After enzyme incubation the resulting protoplast suspension was filtered twice through a 40 µm mesh (Falcon Cell Strainer, Fisher Scientific) into a pre-chilled 50 ml Falcon tube. The protoplasts were collected for 5 min at 200 g at 4°C and the pellet resuspended in 1 ml Solution A without enzyme. A small aliquot of the suspension (20 µl) was stained with 0.05 % w/v Evans Blue dye and the number of protoplasts were counted using a Neubauer hemocytometer. The cells were then diluted to a final concentration of ~ 300 cells / µl and transferred to a 1.5 ml eppendorf tube on ice till usage.

Rosette protoplasts: The rosettes of 40 five-weeks-old plants were harvested 75 min before the ED and before the EN time points. All the above-ground plant material (leaves, petioles and shoot apical meristem (SAM)) was harvested by cutting at the level of the hypocotyl with a scalpel. They were then protoplasted by dicing the tissue in a protoplasting solution (20 mM MES pH5.7, 0.4 M

Manitol, 20 mM KCl, 10 mM $CaCl_2$, 0.1 % w/v BSA, 2 % w/v cellulose RS and 1 % w/v Macherozyme Onozuka R10) (Yoo et al., 2007) with straight stainless steel double edge razor blade and digested for one hour in the growth chamber (12 h light/dark; 22 °C/18 °C) without shaking. Note that harvesting and protoplasting of the EN samples was performed in the dark.
The protoplasts were filtered through a 70 µm mesh (Falcon Cell Strainer, Fisher Scientific) and collected in a 50 ml Falcon tube on ice. Equal volume (3-4 ml) W5 solution (2 mM MES pH 5.7, 154 mM NaCl, 125 mM $CaCl_2$, 5 mM KCl) was added to the remaining tissue to release all the residual protoplasts (Yoo et al., 2007). The protoplasts were then pelleted by centrifugation for 5 min at 500 g and resuspended in 700 µl of resuspension buffer (1:1 protoplasting buffer without enzymes:W5 solution; 4 °C). The protoplast suspension was then layered over 300 µl of a 13.3 % w/v Ficoll cushion and centrifuged for 5 min at 700 g (4 °C). Protoplasted cells forming a layer on top of the Ficoll cushion were collected. An aliquot of the cells was stained with 0.05 % w/v Evans Blue dye and counted using a Neubauer hemocytometer. The protoplast suspension was diluted to a final concentration of ~300 cells / µl in resuspension buffer and kept on ice till usage.

Supplemental Methods S2. Single-cell RNA sequencing with Drop-seq.

The barcoded poly-T oligonucleotides bind to poly-A transcripts which in turn facilitates production of a barcoded cDNA library. Based on the unique barcode identifier the sequenced cDNAs can be assigned to a cell of origin.
Barcoded Oligo dT Primer IB beads (Macosko-2011-10(V+) ChemGenes Corp) were prepared according to the method outlined in Macosko et al. (2015) and single-cell co-encapsulation was performed with the protoplast suspension using the first generation Drop-seq microfluidic device and glass chip (Dolomite Bio). Both protoplasts and beads were loaded at a concentration of 300 objects / µL. Droplet formation occurred as the cell suspension and bead suspension streams merge in a junction of the microfluidics chip and are coencapsulated immediately by a stream of QX200 Droplet Generation oil (BioRad) (Supplemental Fig. S1C). The flow rates of the bead and cell suspensions were 30 µL / min, and the oil was 200 µL / min. The collected droplets were incubated on ice for 5 min to improve polyA-RNA binding to the barcoded beads. Following the Macosko et al. (2015) protocol the droplets were broken by adding 1 ml perfluorooctanol (Sigma) and double strand cDNA synthesis was done with the Maxima H- reverse transcriptase (Thermo Scientific), followed by Exonuclease I (Thermo Scientific) treatment. The resulting cDNA libraries were

divided into eight to ten aliquots each resembling ~ 8,000 beads and the cDNA was amplified (15 PCR cycles) using KapaHiFi HotStnextart ReadyMix. The amplified cDNA fragments were pooled back to original number of samples, purified using 0.6 × volumes of Agencourt AMPure XP beads (Beckman Coulter), and eluted in 15 µL water. The quality of the cDNA libraries was assessed using BioAnalyzer High Sensitivity Chip (Agilent). For next generation sequencing 1 ng of each library was tagmented and amplified (12 cycles) with the Nextera XT v2 DNA sample preparation kit (Illumina) using custom primers as described in (Macosko et al., 2015). Tagmented cDNA libraries were purified twice with 0.6 × volumes of AMPure XP Beads and cDNA fragment distribution and quality was assessed using a BioAnalyzer High Sensitivity Chip. For used primer sequences see Supplemental Table S1. Libraries were submitted to PE150 sequencing using Illumina HiSeq 4000 (BGI Genomic Services, Shenzhen, China). For an overview of the sequenced libraries see Supplemental Table S2.

Supplemental Methods S3. Reference tissue RNAseq.

The whole rosettes from 20 five-weeks-old plants (as described in Supplemental Methods S1) were harvested15 min before ED (and EN) time points and was shock frozen in liquid nitrogen. The tissue was grinded in liquid nitrogen and ~200 mg was used for total RNA extraction with Trizol reagent (Invitrogen) (0.5 mL/ 100 mg tissue). To introduce barcode sequences a barcode-like primer was used having the same polyT sequence, a random sequence corresponding to the cell-specific barcode sequence, and the PCR handle sequence as used with the barcoded beads. For each sample 50 ng of total RNA served as template for the cDNA synthesis.

Supplemental Methods S4. Quality control PCRs.

Prior deep sequencing, the quality of the single-cell and reference tissue cDNA libraries was assessed for the presence of transcripts of interest. For this 100 pg of the amplified root cDNA was submitted to PCR assays using *ACTI*N2 (AT3G18780, ubiquitously expressed), *SUC2* (AT1G22710, phloem and stomata expressed), YFP and *WOX5* (AT3G11260, root apical meristem (RAM) cell marker) specific primer pairs (Supplemental Fig.S2A). For all PCR assays sets, except for *WOX5*, 35 PCR cycles were used. For *WOX5* amplicons 40 PCR cycles were used. For the rosette samples primers against *ACTIN2*, *SUC2,* and *KNAT1* (AT4G08150, vascular

cambium/ meristem cell marker) were used for 35 cycles. For ED and EN markers primer pairs against *LUX* (AT3G46640) and *LHY* (AT1G01060) were used in 25 PCR cycles (Supplemental Fig.S2C). All reactions were carried out with DreamTaq polymerase (Thermo Fischer) according to the suggested conditions. All PCR primer oligonucleotide sequences are listed in Supplemental Table S1.

Supplemental Methods S5. Clustering and marker identification for single-cell samples.
For root data, cells with > 500 transcripts, > 200 genes, < 20 % mitochondrial genes, < 20 % chloroplast genes and < 20,000 transcripts were retained. For rosette data (ED or EN), cells with > 300 transcripts, > 150 genes, < 5 % mitochondrial genes, < 25 % chloroplast genes and < 10,000 transcripts were retained. The expression counts were log-normalized and variable genes were identified for each sample using VST method (standardized variance cutoff ≥ 1 for root, ≥ 0.98 for ED, ≥ 0.99 for EN). Next the standard deviation of the variance for the variable transcript was calculated and the genes with less than 1 standard deviation were retained as variable genes. These variable genes were used to identify integration anchors to combine the samples. The plastidial, non-annotated, microRNA, and RepTAS genes were also removed from variable gene set. The number of PCs for dimensionality reduction was evaluated using an ElbowPlot and Jackstraw method. For root and rosette data 40 and 30 PCs were used, respectively. The PC values were batch corrected using Harmony v0.99.9 (Korsunsky et al., 2018) with maximum 20 iterations. The corrected PCs were used for clustering (resolution: 0.7) the cells, which were then visualized using t-SNE (perplexity: 40, theta: 0.5). Conserved marker identification was done based on log fold change ≥ 0.25 in at least two out of three replicates and minimum cell percentage ≥ 5 % and *P*-value <0.05. Next, we filtered cells from clusters, which did not contain any specific markers indicating that they could be rather unspecific or low quality (*i.e.*, cluster 0 and 1 for root, cluster 0 and 1 for rosette at ED, cluster 0 for rosette at EN; for cluster visualization before filtering see Supplemental Figure S5). Hence, the Seurat pipeline was repeated from variable gene identification by retaining the variable genes from the initial clustering and adding the newly identified variable genes. For rosette (EN), the cluster resolution was changed to 1.0. Clusters that formed subgroups in the t-SNE visualization were manually split into "a" and "b" sub-clusters. Markers were calculated using FindAllMarkers function of Seurat (fold change ≥ 0.25, minimum cell percentage ≥ 5 %, and adjusted *P*-value < 0.05). We pooled the cells, which were in the final individual ED and EN clustering for the pooled ED/EN clustering and run the same pipeline as

described above using SelectIntegrationFeatures function of Seurat (30 PCs, cluster resolution 0.4). A second clustering approach for pooled ED/EN was performed without batch normalization, *i.e.*, without using Harmony.

Supplemental Methods S6. RNA *in situ* hybridization.

Immunological detection was carried out using Anti-DIG antibody (Roche, Mannheim, Germany) diluted 1:1250 with blocking reagent and colorimetric detection, using the NBT/BCIP solution diluted in TNM-50 supplemented with 10 % w/v polyvinyl alcohol. Probes for *CYCLINB1*;*1* (*CYCB1;1*), *HISTONE 4* (*H4*), and *MERCY1 (AT5G16250)* were generated using DIG RNA Labeling Kit (Roche, Mannheim, Germany) as described previously. Primers used for generating *MERCY1* probe are provided in Supplemental Table S1. *CYCB1;1* and *H4* probes were previously described (Omidbakhshfard et al., 2018).

Supplemental Methods S7. Growth and morphology analysis.

Eight seedlings were placed on each plate, arranged such that Col-0 and mutant seedlings were in alternating positions. The position of the root tips was indicated by scoring the back of the plate. Plates were then returned to LD growth conditions for 7 days after which the plates were photographed and the root lengths measured using ImageJ (Abràmoff et al., 2004). After the plates were photographed, the roots were mounted on a slide with 0.5 × MS 0.6 % w/v sucrose as the mounting medium. Root meristem lengths were viewed using an Olympus BX61 light microscope and the meristem lengths were measured using the line measure tool in Leica Application Suite. Meristem length was measured as the linear distance between the stem cell niche and the first observable differentiating cortical cell.

Supplemental Methods S8. RT-qPCR of *CK1* / *CK1-TLS* and non-annotated transcript.

For the standard curve, *CK1* (*AT4G26100*) and *CK1-TLS* fragments were produced by PCR from cDNA extracted from whole Col-0 seedlings. The PCR products were isolated from agarose gels and purified using GeneJet Gel Extraction Kit (Thermo Fisher). The concentrations of the purified fragments were measured by NanoDrop and then diluted and mixed to produce the standards for the standard curve in the concentrations and ratios described. RT-qPCR on both the standard curve and the FACS samples was done using SYBR Green and 7900HT Fast RealTime PCR System

(Thermo Fisher) using CK1 forward primer (FP) and CK1 reverse primer (RP) for *CK1* and CK1 FP and CK1-TLS RP primers for *CK1-TLS* (Zhang et al., 2016). *UBQ10* (AT4G05320) was used as the reference control. Primer sequences are listed in Supplemental Table S1.

For the non-annotated transcript RT-PCR, cDNA was made from total RNA isolated from whole 14-day old Col-0 seedlings using Reverse Transcriptase (Promega) in the presence of RT primers specific for *ATNG47* and *ACTIN2* to produce the cDNA for RT-PCR. For the *ATNG47* positive strand samples *ATNG47* FP was used in the RT reaction. For the negative strand samples ATNG47 RP was used in the RT reaction. Genomic DNA was isolated from 14 day old Col-0 seedlings by grinding tissue in Extraction Buffer (200 mM Tris-HCl pH 8, 150 mM NaCl, 25 mM EDTA, 0.5 % w/v SDS), purified using Phenol:Chloroform:Isoamyl, and finally precipitated overnight in 100 % isopropanol.
PCR amplification of *ACTIN2* was done using ACTIN2 FP and RP using 50 cycles, and 35 cycles for the genomic DNA samples. PCR of the 1050 bp region of *ATNG47* was done using *ATNG47* FP and *ATNG47* RP (Supplementary Table 1).

Supplemental Methods S9. Annotation of Arabidopsis reference genome and alignment.
The RNAseq reads were filtered for rRNA using SortMeRNA v2.1 (Kopylova et al., 2012) with default parameters. The filtered reads were mapped to Arabidopsis reference genome (TAIR10) using STAR (version 2.7.1a; Dobin et al. (2013)) with the following parameters: "—alignIntronMax 12000", "—twopassMode Basic", "—outFilterMismatchNmax 999", "—outFilterMismatchNoverLmax 0.04", "—chimSegmentMin 20", "—chimJunctionOverhangMin 20", "—chimSegmentReadGapMax 3", "—alignSJDBoverhangMin 1", "—outFilterScoreMinOverLread 0.3" and "—outFilterMatchNminOverLread 0.3". The alignments were then assembled into potential transcripts using StringTie v2.0 (Pertea et al., 2015). Non-annotated transcripts were assigned a known TAIR code if the coordinates and strand overlapped. Otherwise, non-annotated loci and corresponding transcripts were assigned a new gene name with ATNG as a prefix. The coordinates for all the non-annotated loci and transcripts are provided in Supplemental Table S3. The annotations from Araport11, RepTAS, miRBase, and non-annotated transcripts were combined as a single genome annotation which was used for all subsequent analysis unless stated otherwise.

The scRNAseq and reference RNAseq samples were processed using the Drop-seq computational pipeline (release 2.3.0; https://github.com/broadinstitute/Drop-seq/) with default parameters except for TagReadWithGeneFunction. The reads were mapped to Arabidopsis reference genome (TAIR10) using STAR with the parameters mentioned earlier. A read was assigned to a gene using TagReadWithGeneFunction utility in Drop-seq with a preference for Araport11 annotation followed by RepTAS and then non-annotated genes. The root and rosette samples were analyzed independently, unless specified otherwise. The final alignment files obtained from Drop-seq pipeline were used for quantifying expression of all genes using htseq (version 0.12.4). The raw counts were further normalized using DESeq2 (Love et al., 2014).

References

Abràmoff MD, Magalhães PJ, Ram SJ (2004) Image processing with ImageJ. Biophotonics international **11:** 36-42

Dobin A, Davis CA, Schlesinger F, Drenkow J, Zaleski C, Jha S, Batut P, Chaisson M, Gingeras TR (2013) STAR: ultrafast universal RNA-seq aligner. Bioinformatics **29:** 15-21

Kopylova E, Noé L, Touzet H (2012) SortMeRNA: fast and accurate filtering of ribosomal RNAs in metatranscriptomic data. Bioinformatics **28:** 3211-3217

Korsunsky I, Fan J, Slowikowski K, Zhang F, Wei K, Baglaenko Y, Brenner M, Loh P-R, Raychaudhuri S (2018) Fast, sensitive, and flexible integration of single cell data with Harmony. Biorxiv**:** 461954

Love MI, Huber W, Anders S (2014) Moderated estimation of fold change and dispersion for RNA-seq data with DESeq2. Genome biology **15:** 1-21

Macosko EZ, Basu A, Satija R, Nemesh J, Shekhar K, Goldman M, Tirosh I, Bialas AR, Kamitaki N, Martersteck EM (2015) Highly parallel genome-wide expression profiling of individual cells using nanoliter droplets. Cell **161:** 1202-1214

Omidbakhshfard MA, Fujikura U, Olas JJ, Xue G-P, Balazadeh S, Mueller-Roeber B (2018) GROWTH-REGULATING FACTOR 9 negatively regulates arabidopsis leaf growth by controlling ORG3 and restricting cell proliferation in leaf primordia. PLoS genetics **14:** e1007484

Pertea M, Pertea GM, Antonescu CM, Chang T-C, Mendell JT, Salzberg SL (2015) StringTie enables improved reconstruction of a transcriptome from RNA-seq reads. Nature Biotechnology **33:** 290-295

Yoo S-D, Cho Y-H, Sheen J (2007) Arabidopsis mesophyll protoplasts: a versatile cell system for transient gene expression analysis. Nature protocols **2:** 1565

Zhang W, Thieme CJ, Kollwig G, Apelt F, Yang L, Winter N, Andresen N, Walther D, Kragler F (2016) tRNA-Related Sequences Trigger Systemic mRNA Transport in Plants. Plant Cell **28:** 1237-1249

Supplemental Text S1. MapMan annotation of protoplast-induced genes.

We also looked for an enrichment of those protoplast-induced (up-regulated) genes in the MapMan functional categories (Supplemental Fig. S4B). As expected, the category 26 (external stimuli response) is significantly enriched in all four groups (root, ED, EN, common in ED and EN) mainly due to enrichment in several subcategories such as pathogen (26.8), temperature (26.4), and damage (26.7) responses. However, this affects only a minor proportion (~3-5 %) of the protoplast-induced genes (*e.g.*, 126 from 3,760 genes with MapMan annotation in root; 112 from 2,325 in ED+EN), whereas the majority is distributed among all functional MapMan categories. Other significantly enriched categories among protoplast-induced genes are chromatin organisation (12), protein modification (18), cytoskeleton organisation (20).

Supplemental Text S2. ED cluster annotation.

Clusters 0,1,2,3,4,7,13, 24, 27 were annotated as mesophyll cells because of the abundance of transcripts for mesophyll marker genes *CHLOROPHYLL A/B-BINDING PROTEIN 2* (*CAB2,* AT1G29920) (Millar et al., 1992) and *LIGHT-HARVESTING CHLOROPHYLL B-BINDING 2* (*LHCB2.1*, AT2G05100) (Crepin et al., 2015).

We were first unable to successfully identify Cluster 6 as the only marker gene assigned to the cluster is a non-annotated region (AT3G06365) that was not analyzed in its expression or function in previous reports. However, we assigned it as mesophyll cells based on the cell's occurrence in in the pooled ED/EN clustering (cluster 0; mesophyll cells, see main text, Supplemental Table S14).

Clusters 8 and 11 were annotated as senescing cells of the leaf or petiole because of the presence of senescence associated genes *ALLENE OXIDE CYCLASE 1,2,3* (*AOC1*, AT3G25760; *AOC2*, AT3G25770; *AOC3*, AT3G25780) (Stenzel et al., 2012) and *SENESCENCE-ASSOCIATED GENE 21* (*SAG21*, AT4G02380) (Miller et al., 1999).

Clusters 9, 17, and 21 were also annotated as senescent cells due to the specificity of the marker genes such as *ATWRKY22* (AT4G01250) (Zhou et al., 2011) and based on the cell's occurrence in in the pooled ED/EN clustering (cluster 2; senescent cells, see main text, Supplemental Table S14). Moreover, cluster 9 and 17 might be (senescent) parenchyma cells based on the marker gene *NAD KINASE 1* (*NADK1,* AT3G21070) (Waller et al, 2010).

Cluster 5 was identified as phloem cells due to the presence of transcripts of *METALLOTHIONEIN 1* (*MT1,* AT1G07600) (Guo et al., 2003, Brady et al., 2007) as in the pooled ED and EN clustering (see main text).

Clusters 10, 15, 16, 20, 23, 26 and 29 are epidermal cells as they are marked by known epidermal genes *LIPID TRANSFER PROTEIN 1* (*LP1,* AT2G38540) (Clark and Bohnert, 1999), *ATML1* (AT4G21750) (Lu et al., 1996, Sessions et al., 1999) and genes associated with the production of the cuticle layer, *3-KETOACYL-COA SYNTHASE 1* (*KCS1,* AT1G01120), *3-KETOACYL-COA SYNTHASE 6* (*KCS6*, AT1G68530) and *3-KETOACYL-COA SYNTHASE 10* (*KCS10*, AT2G26250) (Suh et al., 2005).

We identified clusters 12 and 33 as the shoot apical meristem due to the specific expression of *REPRESSOR OF WUSCHEL1* (*ROW1*, AT1G04020) (Zhang et al., 2015) and histone genes (including: *HTA2*, *AT4G27230*; *HTA7*, AT5G27670) which are highly expressed in meristems due to their requirement for chromosome formation after DNA replication during cell division.

Cluster 30 is likely the SAM containing at least in part the stem cell niche due to the co-detection of genes *SHOOT MERISTEMLESS* (*STM,* AT1G62360) (Clark et al., 1996) and *PLETHORA 5* (*PLT5,* AT5G57390) (Prasad et al., 2011). We were unable to detect transcripts for *CLAVATA3* (*CLV3*, AT2G27250) (Brand et al., 2000) and *WUSCHEL* (*WUS*, AT2G17950) (Laux et al., 1996) which led us to believe that there are no organizing center (OC) cells contained within our dataset.

Cluster 14, as in cluster 6 of the pooled dataset (see main text), represents the endodermal cells close to the bundle sheath cells. This is marked by both markers for glucosinolate biosynthesis *CYTOCHROME P450* (*CYP83A1,* AT4G13770) (Nintemann et al., 2018) and *BRANCHED-CHAIN AMINOTRANSFERASE4* (*BCAT4,* AT3G19710) (Meena et al., 2019) and statocyte specific marker genes *LAZY1* (AT5G14090) (Yoshihara et al., 2013), *LAZY2* (AT1G17400) (Taniguchi et al., 2017).

Cluster 19 we identified as myrosine cells due to the strong and specific abundance of *THIOGLUCOSIDE GLUCOHYDROLASE 1* (*TGG1,* AT5G26000) and *GLUCOSIDE GLUCOHYDROLASE 2* (*TGG2,* AT5G25980) (Shirakawa et al., 2016).

Clusters 18, 22, 25, and 28 appear to be cells that are responding to pathogens by the expression of known defense genes *MILDEW RESISTANCE LOCUS O 12* (*MLO12*, AT2G39200), *BETA-1,3-GLUCANASE 2* (*BGL2,* AT3G57260), and *PATHOGENESIS-RELATED GENE 1* (*PR1,* AT2G14610) (van Verk et al., 2008). These clusters, it should be noted, also bear similarity to other clusters suggesting that it is possible to identify the tissue type of the clusters. Cluster 18 can

be identified as vasculature associated cells, clusters 22 and 25 appear to be mesophyll cells and cluster 28 is the shoot apical meristem by similarity to other clusters.

Clusters 31 and 36 are annotated as 'proliferating cells' due to the specific incidence of cell-cycle genes including *HINKEL* (*HIK*, AT1G18370) (Strompen et al., 2002), and our previously unknown marker *AT5G16250*. Included in cluster 31 are transcripts of *TOO MANY MOUTHS* (*TMM,* AT1G80080) (Bhave et al., 2009), *SPEECHLESS* (*SPCH,* AT5G53210) (Lampard et al., 2008) and *SCREAM* (*SCRM,* AT3G26744) (Kanaoka et al., 2008), which suggest that this cluster also contains proliferating stomata initials. Cluster 36, in contrast, features elevated numbers of *PLT5* transcripts suggesting that these are proliferating cells in the SAM alone.

Stomata marker genes including *TMM* (AT1G80080), *SPCH* (AT5G53210), *SCRM* (AT3G26744) and *FAMA* (AT3G24140) (Shirakawa et al. 2014) are distributed throughout the clusters and so our interpretation is that we do not find a clear single cluster for stomata cells.

Cluster 32 is marked by an abundance of heat shock genes (including *HSP17.6C-CI* (AT1G53540) and *HEAT SHOCK PROTEIN 70* (*HSP70*, AT3G12580) (Wu et al., 2020)) which leads us to conclude that these are cells that are experiencing moderate to severe heat stress and thus their transcriptomes are dominated by the response to this external stimulus.

Cluster 34 we identify as procambial cells because of the marker gene *PHLOEM INTERCALATED WITH XYLEM* (*PXY,* AT5G61480) (Shi et al., 2019) being found specifically here. Cluster 35 is the collected tissues of the provascular tissues due to the inclusion of multiple markers for cells of phloem and xylem early developmental stages including *XYLEM CYSTEINE PEPTIDASE 2* (*XCP2*, AT1G20850) (Avci et al., 2008), *HOMEOBOX GENE 8* (*ATHB-8*, AT4G32880) (Yamazaki et al., 2018) and *ATHB-15* (AT1G52150) (Williams et al., 2005).

Mapman functional category "external stimuli response" is significantly enriched among markers of clusters annotated as epidermis (15, 23, 26) and (senescent) parenchyma (9), as well as pathogen response (22, 25), mesophyll (27) and provascular cells (35). However, these only count for less than 5% of all markers in these clusters.

Supplemental Text S3. EN cluster annotation.

The clusters from the end of night experiment were annotated using the same marker genes described above. Thus, clusters 0, 1, 2, and 6 are annotated as mesophyll by the marker genes *CHLOROPHYLL A/B BINDING PROTEIN 3* (*CAB3,* AT1G29910) and (*LHCB2.1,* AT2G05100). Similarly, clusters 4, 5, 8, 10, 13, 17, 20 and 22 are the epidermis using *LP1* (AT2G38540), *ML1* (AT4G21750), *KCS1* (AT1G01120), *KCS6* (AT1G68530) and *KCS10* (AT2G26250). 3, 18, 21 are probably senescent cells, similar as described for ED (see above) and pooled ED/EN (see main text). Clusters 19 is vasculature due to the marker gene *XTH18* (AT4G30280).

Clusters 7, 14 and 16 are the SAM as indicated by the marker gene *ROW1* and cluster 24 is proliferating cells as evidenced by the markers *AURORA1* (AT4G32830), *HINKEL* (AT1G18370) and our previously unknown marker *AT5G16250.* Clusters 9, 11, and 23 are cells responding to external pathogens as indicated by the presence of transcripts of the genes *LSH10* (AT2G42610), *FUR1* (AT4G05120) and *PR1* (AT2G14610).

Cluster 12 is very similar to cluster 14 of the ED dataset and so we annotated that as endodermal cells too. Accordingly, the cluster contains the marker genes *CYP83A1* (AT4G13770), *BCAT4* (AT3G19710), *LAZY1* (AT5G14090), *LAZY2* (AT1G17400) and *SGR9* (AT5G02750). Cluster 15 is annotated as the myrosine cells due to the abundance of the transcripts TGG1 (AT5G26000) and *TGG2* (AT5G25980).

Mapman functional category “external stimuli response” is significantly enriched among markers of clusters annotated as epidermis (5, 20, 22) as well as SAM (14) and vasculature (19). However, these only count for less than 5% of all markers in these clusters except for cluster 20 (21/341 equals approx. 6.2%).

References

Millar, Andrew J., Sharla R. Short, Nam Hai Chua, and Steve A. Kay. "A novel circadian phenotype based on firefly luciferase expression in transgenic plants." *The Plant Cell* 4, no. 9 (1992): 1075-1087

Crepin, Aurelie, and Stefano Caffarri. "The specific localizations of phosphorylated Lhcb1 and Lhcb2 isoforms reveal the role of Lhcb2 in the formation of the PSI-LHCII supercomplex in Arabidopsis during state transitions." *Biochimica et Biophysica Acta (BBA)-Bioenergetics* 1847, no. 12 (2015): 1539-1548

Stenzel, Irene, Markus Otto, Carolin Delker, Nils Kirmse, Diana Schmidt, Otto Miersch, Bettina Hause, and Claus Wasternack. "ALLENE OXIDE CYCLASE (AOC) gene family members of Arabidopsis thaliana: tissue-and organ-specific promoter activities and in vivo heteromerization." *Journal of experimental botany* 63, no. 17 (2012): 6125-6138

Miller, Jennifer D., Richard N. Arteca, and Eva J. Pell. "Senescence-associated gene expression during ozone-induced leaf senescence in Arabidopsis." *Plant Physiology* 120, no. 4 (1999): 1015-1024

Guo, Woei-Jiun, Weenun Bundithya, and Peter B. Goldsbrough. "Characterization of the Arabidopsis metallothionein gene family: tissue-specific expression and induction during senescence and in response to copper." *New Phytologist* 159, no. 2 (2003): 369-381

Brady, Siobhan M., David A. Orlando, Ji-Young Lee, Jean Y. Wang, Jeremy Koch, José R. Dinneny, Daniel Mace, Uwe Ohler, and Philip N. Benfey. "A high-resolution root spatiotemporal map reveals dominant expression patterns." *Science* 318, no. 5851 (2007): 801-806

Waller, Jeffrey C., Preetinder K. Dhanoa, Uwe Schumann, Robert T. Mullen, and Wayne A. Snedden. "Subcellular and tissue localization of NAD kinases from Arabidopsis: compartmentalization of de novo NADP biosynthesis." *Planta* 231, no. 2 (2010): 305-317

Clark, Anna M., and Hans J. Bohnert. "Cell-specific expression of genes of the lipid transfer protein family from Arabidopsis thaliana." *Plant and Cell Physiology* 40, no. 1 (1999): 69-76

Lu, Pengzhe, Ron Porat, Jeanette A. Nadeau, and Sharman D. O'Neill. "Identification of a meristem L1 layer-specific gene in Arabidopsis that is expressed during embryonic pattern formation and defines a new class of homeobox genes." *The Plant Cell* 8, no. 12 (1996): 2155-2168

Sessions, Allen, Detlef Weigel, and Martin F. Yanofsky. "The Arabidopsis thaliana MERISTEM LAYER 1 promoter specifies epidermal expression in meristems and young primordia." *The Plant Journal* 20, no. 2 (1999): 259-263

Suh, Mi Chung, A. Lacey Samuels, Reinhard Jetter, Ljerka Kunst, Mike Pollard, John Ohlrogge, and Fred Beisson. "Cuticular lipid composition, surface structure, and gene expression in Arabidopsis stem epidermis." *Plant Physiology* 139, no. 4 (2005): 1649-1665

Zhang, Yuzhou, Yue Jiao, Zhaohui Liu, and Yu-Xian Zhu. "ROW1 maintains quiescent centre identity by confining WOX5 expression to specific cells." *Nature communications* 6, no. 1 (2015): 1-8

Shirakawa, Makoto, Haruko Ueda, Tomoo Shimada, and Ikuko Hara-Nishimura. "Myrosin cells are differentiated directly from ground meristem cells and are developmentally independent of the vasculature in Arabidopsis leaves." *Plant signaling & behavior* 11, no. 4 (2016): e1150403

van Verk, Marcel C., Dimitri Pappaioannou, Lyda Neeleman, John F. Bol, and Huub JM Linthorst. "A novel WRKY transcription factor is required for induction of PR-1a gene expression by salicylic acid and bacterial elicitors." *Plant Physiology* 146, no. 4 (2008): 1983-1995

Clark, Steven E., Steven E. Jacobsen, Joshua Z. Levin, and Elliot M. Meyerowitz. "The CLAVATA and SHOOT MERISTEMLESS loci competitively regulate meristem activity in Arabidopsis." *Development* 122, no. 5 (1996): 1567-1575

Prasad, Kalika, Stephen P. Grigg, Michalis Barkoulas, Ram Kishor Yadav, Gabino F. Sanchez-Perez, Violaine Pinon, Ikram Blilou et al. "Arabidopsis PLETHORA transcription factors control phyllotaxis." *Current Biology* 21, no. 13 (2011): 1123-1128

Brand, Ulrike, Jennifer C. Fletcher, Martin Hobe, Elliot M. Meyerowitz, and Rüdiger Simon. "Dependence of stem cell fate in Arabidopsis on a feedback loop regulated by CLV3 activity." *Science* 289, no. 5479 (2000): 617-619

Laux, Thomas, K. F. Mayer, Jürgen Berger, and G. Jurgens. "The WUSCHEL gene is required for shoot and floral meristem integrity in Arabidopsis." *Development* 122, no. 1 (1996): 87-96

Strompen, Georg, Farid El Kasmi, Sandra Richter, Wolfgang Lukowitz, Farhah F. Assaad, Gerd Jürgens, and Ulrike Mayer. "The Arabidopsis HINKEL gene encodes a kinesin-related protein involved in cytokinesis and is expressed in a cell cycle-dependent manner." *Current Biology* 12, no. 2 (2002): 153-158

Bhave, Neela S., Kira M. Veley, Jeanette A. Nadeau, Jessica R. Lucas, Sanjay L. Bhave, and Fred D. Sack. "TOO MANY MOUTHS promotes cell fate progression in stomatal development of Arabidopsis stems." *Planta* 229, no. 2 (2009): 357-367

Kanaoka, Masahiro M., Lynn Jo Pillitteri, Hiroaki Fujii, Yuki Yoshida, Naomi L. Bogenschutz, Junji Takabayashi, Jian-Kang Zhu, and Keiko U. Torii. "SCREAM/ICE1 and SCREAM2 specify three cell-state transitional steps leading to Arabidopsis stomatal differentiation." *The Plant Cell* 20, no. 7 (2008): 1775-1785

Lampard, Gregory R., Cora A. MacAlister, and Dominique C. Bergmann. "Arabidopsis stomatal initiation is controlled by MAPK-mediated regulation of the bHLH SPEECHLESS." *Science* 322, no. 5904 (2008): 1113-1116

Nintemann, Sebastian J., Pascal Hunziker, Tonni G. Andersen, Alexander Schulz, Meike Burow, and Barbara A. Halkier. "Localization of the glucosinolate biosynthetic enzymes reveals distinct spatial patterns for the biosynthesis of indole and aliphatic glucosinolates." *Physiologia plantarum* 163, no. 2 (2018): 138-154

Meena, Mukesh Kumar, Ramgopal Prajapati, Deepthi Krishna, Keerthi Divakaran, Yogesh Pandey, Michael Reichelt, M. K. Mathew, Wilhelm Boland, Axel Mithöfer, and Jyothilakshmi Vadassery. "The Ca2+ channel CNGC19 regulates Arabidopsis defense against Spodoptera herbivory." *The Plant Cell* 31, no. 7 (2019): 1539-1562

Yoshihara, Takeshi, Edgar P. Spalding, and Moritoshi Iino. "A t LAZY 1 is a signaling component required for gravitropism of the A rabidopsis thaliana inflorescence." *The Plant Journal* 74, no. 2 (2013): 267-279

Taniguchi, Masatoshi, Masahiko Furutani, Takeshi Nishimura, Moritaka Nakamura, Toyohito Fushita, Kohta Iijima, Kenichiro Baba et al. "The Arabidopsis LAZY1 family plays a key role in gravity signaling within statocytes and in branch angle control of roots and shoots." *The Plant Cell* 29, no. 8 (2017): 1984-1999

Nakamura, Moritaka, Masatsugu Toyota, Masao Tasaka, and Miyo Terao Morita. "An Arabidopsis E3 ligase, SHOOT GRAVITROPISM9, modulates the interaction between statoliths and F-actin in gravity sensing." *The Plant Cell* 23, no. 5 (2011): 1830-1848

Shirakawa, Makoto, Haruko Ueda, Atsushi J. Nagano, Tomoo Shimada, Takayuki Kohchi, and Ikuko Hara-Nishimura. "FAMA is an essential component for the differentiation of two distinct cell types, myrosin cells and guard cells, in Arabidopsis." *The Plant Cell* 26, no. 10 (2014): 4039-4052

Wu, Xuan, Jie Wang, Xiaohui Wu, Yiling Hong, and Qingshun Quinn Li. "Heat Shock Responsive Gene Expression Modulated by mRNA Poly (A) Tail Length." *Frontiers in plant science* 11 (2020): 1255

Shi, Dongbo, Ivan Lebovka, Vadir López-Salmerón, Pablo Sanchez, and Thomas Greb. "Bifacial cambium stem cells generate xylem and phloem during radial plant growth." *Development* 146, no. 1 (2019)

Avci, Utku, H. Earl Petzold, Ihab O. Ismail, Eric P. Beers, and Candace H. Haigler. "Cysteine proteases XCP1 and XCP2 aid micro-autolysis within the intact central vacuole during xylogenesis in Arabidopsis roots." *The Plant Journal* 56, no. 2 (2008): 303-315

Williams, Leor, Stephen P. Grigg, Mingtang Xie, Sioux Christensen, and Jennifer C. Fletcher. "Regulation of Arabidopsis shoot apical meristem and lateral organ formation by microRNA miR166g and its AtHD-ZIP target genes." *Development* 132, no. 16 (2005): 3657-3668

Yamazaki, Kyoko, Yuki Kondo, Mikiko Kojima, Yumiko Takebayashi, Hitoshi Sakakibara, and Hiroo Fukuda. "Suppression of DELLA signaling induces procambial cell formation in culture." *The Plant Journal* 94, no. 1 (2018): 48-59

Zhou X, Jiang Y, Yu D “WRKY22 transcription factor mediates dark-induced leaf senescence in Arabidopsis.” *Molecules and cells*, no. 31 (2011): 303-313

Supplemental Text S4. ED/EN cluster annotation.

Clusters 1 and 15 were annotated as mesophyll cells due to the presence of *CAB3* (AT1G29910), *LHCB2.1* (AT2G05100), and *CARBONIC ANHYDRASE 1* (*CA1,* AT3G01500) (Endo et al., 2014; Uemoto et al., 2018). Cluster 0 represents probably also mesophyll cells, however, the information content (number of genes per cell) is relatively low (Supplemental Table S9).

Cluster 6 contains the transcripts of the genes *CYP83A1* (AT4G13770) and *BCAT4* (AT3G19710) that are involved in the glucosinolate biosynthesis in Brassicaceae-specific S-cells. Cluster 6 also contains *KNAT1* (AT4G08150) transcripts, which are associated with meristem boundaries and vascular differentiation as well as the transcripts of *LAZY1* (AT5G14090), *LAZY2* (AT1G17400), and *SGR9* (AT5G02750), which are expressed in statocytes. Therefore, we concluded that cluster 6 represents the endodermal cells between the vasculature and the bundle sheath of petioles (Sensing et al., 2011; Taniguchi et al., 2017).

Clusters 2 and 9 were annotated as senescent leaves/petioles based on the presence of the marker transcripts *ATWRKY22* (AT4G01250) (Zhou et al., 2011), *SAG14* (AT5G20230), and *ATCPK32* (AT3G57530) expression reported in Klepikova et al. (2016). Furthermore, cluster 9a can be annotated as (senescent) parenchyma cells based on the marker gene *NADK1* (AT3G21070) (Waller et al., 2010).

Cluster 12 was identified as phloem tissue due to the inclusion of the phloem markers *MT3* (AT3G15353), *DOT1* (AT2G36120), and *SULTR2;1* (AT5G10180) (Bonke et al., 2003; Brady et al., 2007; Petricka et al., 2008).

Clusters 3, 7, and 8 were annotated as epidermis cells because of the specific expression of *ATML1* (AT4G21750), *KCS1* (AT1G01120), *KCS6* (AT1G68530), and *CER5* (AT1G51500) genes (Pighin et al., 2004; Takada et al., 2013). Cluster 3 seems to represent abaxial leaf cells based on the presence of the marker transcripts *MAGL6 (*AT2G39400*), MO1* (AT4G15760), and *AT5G28610* expression detected in TRAP assays (Tian et al., 2014). In line, cluster 3 and 7 also contain relative high amounts of the guard cell markers *FLP* (AT1G14350) and *CYP86A2* (AT4G00360) (Lai et al., 2005; Francia et al., 2008). Cluster 7 is also enriched in transcripts falling into the MapMan categories lignin (21.6), cutin/suberin (21.9), light stimulus (26.1) and FAE (5.1.6) (Supplemental Fig. S8). The cutin genes correspond to the annotation as epidermal cells, and the light stimulus gene seems to suggest that this cluster represents the adaxial epidermis of leaves.

Clusters 4 and 11 were assigned to be different regions of the SAM. Cluster 4 is enriched in meristem marker genes *ROW1* (AT1G04020) and *RAD51* (AT5G20850). Cluster 4 is also enriched in transcripts of the MapMan categories annotated as nucleotide metabolism (6) and chromatin organisation (12, 12.1). Cluster 11 was found to have high *LSH4* (AT3G23290) expression and so we annotated it as the boundary region of the meristem.

Cluster 13 has specific expression of an unknown marker (*AT5G16250*, see below), as well as *AURORA1* (AT4G32830). This cluster also specifically contains stomata-precursor cell markers *SPCH* (AT5G53210) and *TMM* (AT1G80080), which suggests that these are dividing cells in the process of stomata formation (Kanaoka et al., 2008). Cluster 13 is enriched in the MapMan categories cell cycle organisation (13), chromatin remodelling (12.4), DNA methylation (12.5), mRNA silencing (16.10), RNA chaperones (16.7) which supports our cluster annotation as dividing cells (Supplemental Fig. S8).

Cluster 5 was annotated as myrosine cells having the cell type specific *TGG1* (AT5G26000) and *TGG2* (AT5G25980) marker transcripts (Shirakawa et al., 2016). However, this cluster seems also to contain mature guard cells as *TGG1* and *TGG2* are also expressed in mature guard cells.

Cluster 10 has specific expression of *PR1* (AT2G14610), *PR2* (AT3G57260), and *PR5* (AT1G75040) genes. These are genes involved in the acquired resistance induced by salicylic acid (Uknes et al., 1992), and so we predict that these cells are responding to pathogens, which is in line with the growth under non-sterile conditions on soil.

Cluster 14 was assigned to water/cold stress responsive cells based on the marker transcripts belonging to EARLY RESPONSIVE TO DEHYDRATION family and *GRP7/CCR2* known to be responsive to stress.

References

Bonke M, Thitamadee S, Mähönen AP, Hauser M-T, Helariutta Y (2003) APL regulates vascular tissue identity in Arabidopsis. Nature 426: 181-186

Brady SM, Orlando DA, Lee J-Y, Wang JY, Koch J, Dinneny JR, Mace D, Ohler U, Benfey PN (2007) A high-resolution root spatiotemporal map reveals dominant expression patterns. Science 318: 801-806

Francia P, Simoni L, Cominelli E, Tonelli C, Galbiati M (2008) Gene trap-based identification of a guard cell promoter in Arabidopsis. Plant signaling & behavior 3: 684-686

Kanaoka MM, Pillitteri LJ, Fujii H, Yoshida Y, Bogenschutz NL, Takabayashi J, Zhu J-K, Torii KU (2008) SCREAM/ICE1 and SCREAM2 specify three cell-state transitional steps leading to Arabidopsis stomatal differentiation. The Plant Cell 20: 1775-1785

Lai LB, Nadeau JA, Lucas J, Lee E-K, Nakagawa T, Zhao L, Geisler M, Sack FD (2005) The Arabidopsis R2R3 MYB proteins FOUR LIPS and MYB88 restrict divisions late in the stomatal cell lineage. The Plant Cell 17:2754-2767

Petricka JJ, Clay NK, Nelson TM (2008) Vein patterning screens and the defectively organized tributaries mutants in Arabidopsis thaliana. The Plant Journal 56: 251-263

Pighin JA, Zheng H, Balakshin LJ, Goodman IP, Western TL, Jetter R, Kunst L, Samuels AL (2004) Plant cuticular lipid export requires an ABC transporter. Science 306: 702-704

Sensing G, Nakamura M, Toyota M, Tasaka M, Morita MT (2011) An Arabidopsis E3 ligase, SHOOT GRAVITROPISM9, modulates the interaction between statoliths and f-actin in. Plant Cell, 23(5), 1830-1848

Shirakawa M, Ueda H, Shimada T, Hara-Nishimura I (2016) Myrosin cells are differentiated directly from ground meristem cells and are developmentally independent of the vasculature in Arabidopsis leaves. Plant Signaling & Behavior 11: e1150403

Takada S, Takada N, Yoshida A (2013) ATML1 promotes epidermal cell differentiation in Arabidopsis shoots. Development 140:1919-1923

Taniguchi M, Furutani M, Nishimura T, Nakamura M, Fushita T, Iijima K, Baba K, Tanaka H, Toyota M, Tasaka M, Morita MT (2017) The arabidopsis LAZY1 family plays a key role in gravity signaling within statocytes and in branch angle control of roots and shoots. Plant Cell, 29(8), 1984–1999

Uknes S, Mauch-Mani B, Moyer M, Potter S, Williams S, Dincher S, Chandler D, Slusarenko A, Ward E, Ryals J (1992) Acquired resistance in Arabidopsis. The Plant Cell 4: 645-656

Supplemental Text S5. Root cluster annotation.

Clusters 2, 7, 8 and 25 are annotated as root hairs because of the specific expression of the marker genes *SHAVEN2* (*SHV2*, AT5G49270) (Ringli et al., 2005), *ROOT HAIR SPECIFIC 9* (*RHS9*, AT1G69240), *GLYCOSYL HYDROLASE 9C1* (*GH9C1,* AT1G48930), *AT3G09330* (Bruex et al., 2012, Brady et al., 2007).

Clusters 5, 17 and 30 are the non-hair epidermis cells. Cluster 5 is identified by the presence of *XTH17* (AT1G65310) (Tominaga-Wada et al., 2009) and *GLABRA 2* (*GL2,* AT1G79840) (Wada et al., 2002). Cluster 17 is marked by *GL2* (*AT1G79840*), *LTPG2* (AT3G43720), *LTPG1* (AT1G27950) (Kim et al., 2012, Brady et al., 2007), *TTG2* (AT2G37260), *AT1G65310* (Bruex et al., 2012, Brady et al., 2007). Cluster 30 is marked by *AT1G65310* (Bruex et al., 2012, Brady et al., 2007).

Clusters 9, 23, and 26 are the cortex layer based on the presence of marker genes *AT1G62510* (Denyer et al., 2019), *E49* (AT3G05150) (Lee et al., 2006, Machin et al., 2019), *IAMT1* (AT5G55250), *AT5G53370* (Che et al., 2006, Brady et al., 2007).

Clusters 10 and 31 are the endodermis cell layer. Cluster 10 is annotated by the marker genes *SCR* (AT3G54220) (Heidstra et al., 2004, Machin et al., 2019), *AT5G45210* and *AT2G14900* which are markers previously identified by the ATH1 dataset as specific to the meristem region of the endodermal layer (Brady et al., 2007). Cluster 31 contains the genes *CASP2* (AT3G11550), *AT4G17215*, and *CASP3* (AT2G27370) which are known markers for the differentiated endodermis, particularly *CASP2* and *CASP3* as they are involved in the production of the Casparian Strip (Roppolo et al., 2011).

Cluster 13 is the xylem pole pericycle, due to the presence of the markers *XTH21* (AT2G18800) (Brady et al., 2007) and *GATA23* (AT5G26930) (Rybel et al., 2010). Clusters 12, 20 and 28 are likely to be the phloem pole pericycle (PPP) as they all have *DOT1* (AT2G36120) (Petricka et al., 2008; Brady et al., 2007) as a marker.

Cluster 15 contains the root cap markers *BEARSKIN1* (AT1G33280), *BEARSKIN2* (AT4G10350) and *SOMBRERO* (AT1G79580) (Bennett et al., 2010) suggesting that these are root cap cells. Programmed cell death marker associated with the lateral root cap *METACASPASE9* (AT5G04200) (Tsiatsiani et al., 2013) is also present.

Cluster 24 was assigned as the companion cells of the phloem, as the companion cell markers *SUC2* (AT1G22710) (Imlau et al., 1999, Machin et al., 2019), *MT3* (AT3G15353) and *APL* (AT1G79430) (Bonke et al., 2003) are specifically located here.

Clusters 21, 22 and 27 represents differentiating xylem as they cells have higher expression of the immature xylem marker *XYLEM CYSTEINE PEPTIDASE 1* (*XCP1*, AT4G35350) (Funk et al., 2002; Zhao et al., 2005). The presence of *VND7* (AT1G71930) in Cluster 22 and *VND6* (AT5G62380) in cluster 27 indicate that these two clusters represent the protoxylem and metaxylem respectively (Kubo et al., 2005; Yamaguchi et al., 2008). Cluster 27 contains also the metaxylem marker *ACL5* (AT5G19530) (Muniz et al., 2008) differentiating xylem vessel markers *TED6* (AT1G43790), TED7 (AT5G48920) vessel elements and *WAT1* (AT1G75500) (Endo et al., 2009; Ranocha et al. 2010, Muñiz et al., 2008). Further, cluster 21 can be identified as xylem associated cells due to the presence of XCP*2* (AT1G20850) (Funk et al., 2002; Zhao et al., 2005).

The procambium is represented by clusters 11, 14, 16, 18 and 19. These clusters were identified initially as cells from the same tissue type due to the overlap of marker genes between these clusters. These clusters were found to be marked by the genes *GST6* (AT1G02930), *GST11* (AT1G02920), *CIMT1* (AT5G37990) and *SABATH FAMILY METHYLTRANSFERASE* (AT5G37970) (Brady et al., 2007).

The remaining clusters were annotated according to developmental stage and cell cycle stage. Cluster 3 is annotated as transit amplifying cells of the root apical meristem marked by *REPRESSOR OF WUSCHEL 1* (*ROW1*, AT1G04020) (Zhang et al., 2015) and *DEL1* (AT3G48160) (Vlieghe et al., 2005, Sozzani et al., 2010). Cluster 4 is annotated as cells of the mature root as there is no specific marker that can identify the cells (*e.g.*, *AT1G07600*, *AT4G17340*) as any one cell type but the cluster contains a high number of transcripts of our V311 marker that marks post-meristem cells. Cluster 29 contains the markers *PLT1* (AT3G20840), *PLT2* (AT1G51190), *PLT3* (AT5G10510) (Shimotohno et al., 2018) and *PIP2;8* (AT2G16850) (Péret et al., 2012) which suggests that these are primary root cells close to or including stem cells or lateral root initials.

Cluster 32 represents cells undergoing division. This is indicated by the specific presence of transcripts for cyclin genes including *CYCLIN B1;3* (AT3G11520), *CYCLIN B2;4* (AT1G76310), and *CYCLIN B1;1* (AT4G37490) (Vandepoele et al., 2002). Additionally, we find other transcripts related to cell division such as *AURORA1* (AT4G32830) (Demidov et al., 2005), and a previously unknown marker *MERCY1* (AT5G16250), which our *in situ* hybridization experiments demonstrate is expressed in dividing cells in the root apical meristem (Figure 5).

Clusters 0,1,6,33,34 were not able to be identified as any particular tissue types. Clusters 0 and 1 did not feature any specific markers and so are unclassified. Cluster 6 is high in rRNAs which suggests that this cluster is formed unspecifically from beads rich in rRNA sequences. Clusters 33 and 34 have very low reads per cell and so could not be assigned a tissue type.

The transcripts for V161 H2B-Venus, which is known to be expressed specifically in the phloem pole pericycle cells, are found specifically in clusters 12 and 28 which we previously annotated as the phloem pole pericycle.

V311 H2B-Venus is expressed in differentiating cells and is found in almost all clusters except for the meristem and proliferating cells. It is found most significantly in the differentiating cortex cluster.

V171 H2B-Venus transcripts are not found highly in our dataset, likely because of an overall low transcript number in this cluster. However, a small number of transcripts were found in the cluster annotated as xylem, the cells adjacent to the xylem pole pericycle.

References

Ringli, Christoph, Nicolas Baumberger, and Beat Keller. "The Arabidopsis root hair mutants der2–der9 are affected at different stages of root hair development." *Plant and cell physiology* 46, no. 7 (2005): 1046-1053

Bruex, Angela, Raghunandan M. Kainkaryam, Yana Wieckowski, Yeon Hee Kang, Christine Bernhardt, Yang Xia, Xiaohua Zheng et al. "A gene regulatory network for root epidermis cell differentiation in Arabidopsis." *PLoS Genet* 8, no. 1 (2012): e1002446

Tominaga-Wada, Rumi, Mineko Iwata, Junji Sugiyama, Toshihisa Kotake, Tetsuya Ishida, Ryusuke Yokoyama, Kazuhiko Nishitani, Kiyotaka Okada, and Takuji Wada. "The GLABRA2 homeodomain protein directly regulates CESA5 and XTH17 gene expression in Arabidopsis roots." *The Plant Journal* 60, no. 3 (2009): 564-574

Wada, Takuji, Tetsuya Kurata, Rumi Tominaga, Yoshihiro Koshino-Kimura, Tatsuhiko Tachibana, Koji Goto, M. David Marks, Yoshiro Shimura, and Kiyotaka Okada. "Role of a positive regulator of root hair development, CAPRICE, in Arabidopsis root epidermal cell differentiation." *Development* 129, no. 23 (2002): 5409-5419

Kim, Hyojin, Saet Buyl Lee, Hae Jin Kim, Myung Ki Min, Inhwan Hwang, and Mi Chung Suh. "Characterization of glycosylphosphatidylinositol-anchored lipid transfer protein 2 (LTPG2) and overlapping function between LTPG/LTPG1 and LTPG2 in cuticular wax export or accumulation in Arabidopsis thaliana." *Plant and Cell Physiology* 53, no. 8 (2012): 1391-1403

Brady, Siobhan M., David A. Orlando, Ji-Young Lee, Jean Y. Wang, Jeremy Koch, José R. Dinneny, Daniel Mace, Uwe Ohler, and Philip N. Benfey. "A high-resolution root spatiotemporal map reveals dominant expression patterns." *Science* 318, no. 5851 (2007): 801-806

Heidstra, Renze, David Welch, and Ben Scheres. "Mosaic analyses using marked activation and deletion clones dissect Arabidopsis SCARECROW action in asymmetric cell division." *Genes & Development* 18, no. 16 (2004): 1964-1969

Machin, Frank Qasim, Malin Beckers, Xin Tian, Alice Fairnie, Teri Cheng, Wolf-Rüdiger Scheible, and Peter Doerner. "Inducible reporter/driver lines for the Arabidopsis root with intrinsic reporting of activity state." *The Plant Journal* 98, no. 1 (2019): 153-164

Lee, Ji-Young, Juliette Colinas, Jean Y. Wang, Daniel Mace, Uwe Ohler, and Philip N. Benfey. "Transcriptional and posttranscriptional regulation of transcription factor expression in Arabidopsis roots." *Proceedings of the National Academy of Sciences* 103, no. 15 (2006): 6055-6060

Che, Ping, Sonia Lall, Dan Nettleton, and Stephen H. Howell. "Gene expression programs during shoot, root, and callus development in Arabidopsis tissue culture." *Plant physiology* 141, no. 2 (2006): 620-637

Heidstra, Renze, David Welch, and Ben Scheres. "Mosaic analyses using marked activation and deletion clones dissect Arabidopsis SCARECROW action in asymmetric cell division." *Genes & Development* 18, no. 16 (2004): 1964-1969

Roppolo, Daniele, Bert De Rybel, Valérie Dénervaud Tendon, Alexandre Pfister, Julien Alassimone, Joop EM Vermeer, Misako Yamazaki, York-Dieter Stierhof, Tom Beeckman, and Niko Geldner. "A novel protein family mediates Casparian strip formation in the endodermis." *Nature* 473, no. 7347 (2011): 380-383

De Rybel, Bert, Valya Vassileva, Boris Parizot, Marlies Demeulenaere, Wim Grunewald, Dominique Audenaert, Jelle Van Campenhout et al. "A novel aux/IAA28 signaling cascade activates GATA23-dependent specification of lateral root founder cell identity." *Current Biology* 20, no. 19 (2010): 1697-1706

Petricka, Jalean Joyanne, Nicole Kho Clay, and Timothy Mark Nelson. "Vein patterning screens and the defectively organized tributaries mutants in Arabidopsis thaliana." *The Plant Journal* 56, no. 2 (2008): 251-263

Bennett, Tom, Albert van den Toorn, Gabino F. Sanchez-Perez, Ana Campilho, Viola Willemsen, Berend Snel, and Ben Scheres. "SOMBRERO, BEARSKIN1, and BEARSKIN2 regulate root cap maturation in Arabidopsis." *The Plant Cell* 22, no. 3 (2010): 640-654

Tsiatsiani, Liana, Evy Timmerman, Pieter-Jan De Bock, Dominique Vercammen, Simon Stael, Brigitte Van De Cotte, An Staes et al. "The Arabidopsis metacaspase9 degradome." *The Plant Cell* 25, no. 8 (2013): 2831-2847

Imlau, Astrid, Elisabeth Truernit, and Norbert Sauer. "Cell-to-cell and long-distance trafficking of the green fluorescent protein in the phloem and symplastic unloading of the protein into sink tissues." *The Plant Cell* 11, no. 3 (1999): 309-322

Bonke, Martin, Siripong Thitamadee, Ari Pekka Mähönen, Marie-Theres Hauser, and Ykä Helariutta. "APL regulates vascular tissue identity in Arabidopsis." *Nature* 426, no. 6963 (2003): 181-186

Funk, Vanessa, Boonthida Kositsup, Chengsong Zhao, and Eric P. Beers. "The Arabidopsis xylem peptidase XCP1 is a tracheary element vacuolar protein that may be a papain ortholog." *Plant Physiology* 128, no. 1 (2002): 84-94

Zhao, Chengsong, Johanna C. Craig, H. Earl Petzold, Allan W. Dickerman, and Eric P. Beers. "The xylem and phloem transcriptomes from secondary tissues of the Arabidopsis root-hypocotyl." *Plant Physiology* 138, no. 2 (2005): 803-818

Kubo, Minoru, Makiko Udagawa, Nobuyuki Nishikubo, Gorou Horiguchi, Masatoshi Yamaguchi, Jun Ito, Tetsuro Mimura, Hiroo Fukuda, and Taku Demura. "Transcription switches for protoxylem and metaxylem vessel formation." *Genes & development* 19, no. 16 (2005): 1855-1860

Yamaguchi, Masatoshi, Minoru Kubo, Hiroo Fukuda, and Taku Demura. "VASCULAR-RELATED NAC-DOMAIN7 is involved in the differentiation of all types of xylem vessels in Arabidopsis roots and shoots." *The Plant Journal* 55, no. 4 (2008): 652-664

Muñiz, Luis, Eugenio G. Minguet, Sunil Kumar Singh, Edouard Pesquet, Francisco Vera-Sirera, Charleen L. Moreau-Courtois, Juan Carbonell, Miguel A. Blázquez, and Hannele Tuominen. "ACAULIS5 controls Arabidopsis xylem specification through the prevention of premature cell death." *Development* 135, no. 15 (2008): 2573-2582

Endo, Satoshi, Edouard Pesquet, Masatoshi Yamaguchi, Gen Tashiro, Mayuko Sato, Kiminori Toyooka, Nobuyuki Nishikubo et al. "Identifying new components participating in the secondary cell wall formation of vessel elements in Zinnia and Arabidopsis." *The Plant Cell* 21, no. 4 (2009): 1155-1165

Ranocha, Philippe, Nicolas Denancé, Ruben Vanholme, Amandine Freydier, Yves Martinez, Laurent Hoffmann, Lothar Köhler et al. "Walls are thin 1 (WAT1), an Arabidopsis homolog of Medicago truncatula NODULIN21, is a tonoplast-localized protein required for secondary wall formation in fibers." *The Plant Journal* 63, no. 3 (2010): 469-483

Zhang, Yuzhou, Yue Jiao, Zhaohui Liu, and Yu-Xian Zhu. "ROW1 maintains quiescent centre identity by confining WOX5 expression to specific cells." *Nature communications* 6, no. 1 (2015): 1-8

Vlieghe, Kobe, Véronique Boudolf, Gerrit TS Beemster, Sara Maes, Zoltan Magyar, Ana Atanassova, Janice de Almeida Engler, Ruth De Groodt, Dirk Inzé, and Lieven De Veylder. "The DP-E2F-like gene DEL1 controls the endocycle in Arabidopsis thaliana." *Current Biology* 15, no. 1 (2005): 59-63

Sozzani, Rosangela, Caterina Maggio, Roberta Giordo, Elisabetta Umana, Jose Trinidad Ascencio-Ibañez, Linda Hanley-Bowdoin, Catherine Bergounioux, Rino Cella, and Diego Albani. "The E2FD/DEL2 factor is a component of a regulatory network controlling cell proliferation and development in Arabidopsis." *Plant molecular biology* 72, no. 4 (2010): 381-395

Shimotohno, Akie, Renze Heidstra, Ikram Blilou, and Ben Scheres. "Root stem cell niche organizer specification by molecular convergence of PLETHORA and SCARECROW transcription factor modules." *Genes & development* 32, no. 15-16 (2018): 1085-1100

Péret, Benjamin, Guowei Li, Jin Zhao, Leah R. Band, Ute Voß, Olivier Postaire, Doan-Trung Luu et al. "Auxin regulates aquaporin function to facilitate lateral root emergence." *Nature cell biology* 14, no. 10 (2012): 991-998

Demidov, Dmitri, Daniël Van Damme, Danny Geelen, Frank R. Blattner, and Andreas Houben. "Identification and dynamics of two classes of aurora-like kinases in Arabidopsis and other plants." *The Plant Cell* 17, no. 3 (2005): 836-848

Vandepoele, Klaas, Jeroen Raes, Lieven De Veylder, Pierre Rouzé, Stephane Rombauts, and Dirk Inzé. "Genome-wide analysis of core cell cycle genes in Arabidopsis." *The Plant Cell* 14, no. 4 (2002): 903-916

Supplemental Text S6. Root Cap and secondary metabolism.

Root cap has a really important role in root meristem protection, gravity sensing, and metabolite secretion to the rhizosphere (Kamiya et al., 2016). In line with these functions the most highly enriched MapMan category for root cap was "glucosinolate degradation" (9.5.2). Glucosinolates (GSLs) are a group of secondary metabolites characteristic of the Brassicaceae and implicated in plant's defense. Pathways and roles of GSLs have been mainly investigated in shoots and in a few studies GSLs have been investigated in separated portions of individual roots. A study performed in field-grown canola roots (Brassica napus; McCully et al., (2008)) attributed a protective role against pests to the continuous release of GSLs, happening together with root expansion, and to their hydrolysis into the rhizosphere. Furthermore McCully et al. (2008) proposed that GSLs released into soil influenced microbial populations associated with long-lived components of the root system. A similar role was proposed for Arabidopsis GSLs secreted from roots to the soil (Poveda, 2021). We can hypothesize that GSLs degradation occurs already in root cap before their release to the rhizosphere and this could justify the high enrichment of the genes classified in that MapMan category.

In line with the demonstrated role of the root cap in metabolite secretion to the soil is the finding of a second MapMan category also related to "Secondary metabolism" that is "terpenoids_Isoprenyl diphosphate biosynthesis" (9.1.3). Prenyl diphosphates are the linear central precursors of all terpenoids. Plants produce a large number of structurally diverse terpenoids which are employed for a variety of basic functions in growth and development but the majority of them are used for more specialized chemical interactions and protection in the abiotic and biotic environment (Tholl, 2015). Volatile, semivolatile and nonvolatile terpenoids are implicated in the protection of plants against abiotic stress and in various biotic interactions above- and below-ground (Loreto et al., 2014). An increased interest in the role of specialized metabolites below ground has shown that terpenoids serve functions similar to those aboveground. Recent studies in Arabidopsis roots discovered semivolatile diterpene hydrocarbons produced in the root stele, from where they diffuse through the surrounding cell layers to function as local antifeedants by reducing root herbivore damage on these cell layers (Vaughan et al., 2013). Nonvolatile terpenoids can be exuded from roots into the rhizosphere and the surrounding soil environment where they are involved in different defense responses (Tholl, 2015). Thus, we suggest a particular involvement of the root cap in the interaction with and protection against the surrounding environment.

References

Kamiya M, Higashio S-Y, Isomoto A, Kim J-M, Seki M, Miyashima S, Nakajima K (2016) Control of root cap maturation and cell detachment by BEARSKIN transcription factors in Arabidopsis. Development 143: 4063-4072

Loreto F, Dicke M, SCHNITZLER JP, Turlings TC (2014) Plant volatiles and the environment. *Plant, cell & environment* 37: 1905-1908

McCully ME, Miller C, Sprague SJ, Huang CX, Kirkegaard JA (2008) Distribution of glucosinolates and sulphur-rich cells in roots of field-grown canola (Brassica napus). *New Phytologist* 180: 193-205

Poveda J (2021) Glucosinolates profile of Arabidopsis thaliana modified root colonization of Trichoderma species. *Biological Control* 155: 104522

Tholl D (2015) Biosynthesis and biological functions of terpenoids in plants. *Biotechnology of isoprenoids*: 63-106

Vaughan MM, Wang Q, Webster FX, Kiemle D, Hong YJ, Tantillo DJ, Coates RM, Wray AT, Askew W, O'Donnell C (2013) Formation of the unusual semivolatile diterpene rhizathalene by the Arabidopsis class I terpene synthase TPS08 in the root stele is involved in defense against belowground herbivory. *The Plant Cell* 25: 1108-1125
